# Methane Beryllation Catalyzed by a Base Metal Complex

**DOI:** 10.1021/jacs.5c02179

**Published:** 2025-03-11

**Authors:** Josef T. Boronski, Agamemnon E. Crumpton, Job J. C. Struijs, Simon Aldridge

**Affiliations:** † Molecular Sciences Research Hub, Department of Chemistry, Imperial College London, 82 Wood Lane, White City, London W12 0BZ, U.K.; ‡ Chemistry Research Laboratory, Department of Chemistry, University of Oxford, 12 Mansfield Road, Oxford OX1 3TA, U.K.

## Abstract

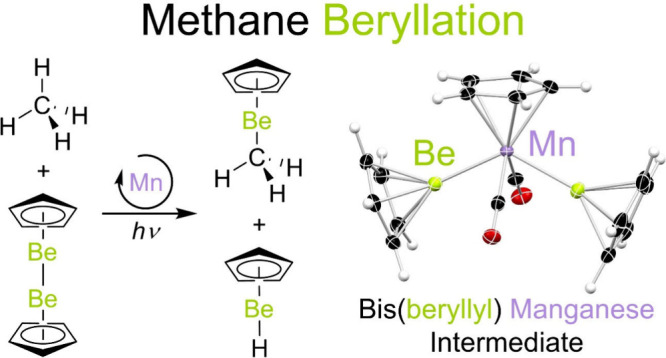

The homogeneous catalytic functionalization of methane is extremely challenging due to the relative nonpolarity and high C–H bond strength of this hydrocarbon. Here, using catalytic quantities (10 mol %) of CpMn(CO)_3_ or Cp*Re(CO)_3_, the conversion of methane and benzene C–H bonds to C–Be and H–Be bonds by CpBeBeCp has been achieved under photochemical conditions. Possible intermediates in the beryllation reactions—*trans*-bis(beryllyl)-manganese and -rhenium complexes—were also isolated. Quantum chemical calculations indicate that the inherent properties of the beryllyl ligands—which are powerfully σ-donating and feature highly Lewis acidic beryllium centers—are decisive in enabling methane functionalization by these systems.

The borylation of hydrocarbon C–H bonds has been a topic of intensive investigation for several decades,^1−6^ in part driven by the synthetic utility of the boryl moiety (BX_2_), which can be straightforwardly converted to a range of alternate functional groups.^7−9^ The first report of catalytic regiospecific alkane borylation detailed the use of cyclopentadienyl manganese- and rhenium-tricarbonyl derivatives, Cp^x^M(CO)_3_ (**1′**: Cp^X^ = C_5_H_4_Me, M = Mn; **2**: Cp^X^ = C_5_Me_5_, M = Re) under photochemical conditions, harnessing B_2_Pin_2_ (Pin = pinacolate) as the boron source.^10^ The corresponding metal–(bis)boryl complexes are key intermediates in these catalytic reactions.^11−13^ While subsequent progress in the field of C–H borylation has been rapid, homogeneous catalysts—particularly those featuring base metals—for the functionalization of the simplest alkane, methane, are limited.^14−20^ Indeed, although methane borylation using a diborane(4) reagent as the boron source is calculated to be thermodynamically downhill, the hydrocarbon’s insolubility, nonpolarity, and high C–H bond strength (104 kcal mol^–1^) make this reaction very challenging in practice.^3^

Beryllium and boron are abutting elements within the second period.^21,22^ However, while the reactivity of diborane(4) derivatives has been thoroughly developed, that of “diberyllanes”—molecules with a Be–Be bond—is almost entirely unknown.^23−28^ We have recently shown that the Be–Be bond of CpBeBeCp (**3**) adds to low-oxidation state metal centers similarly to B–B bonds.^29^ As such, and with the aforementioned reactivity of **1′** and **2** in mind, we were interested to examine the competency of manganese- and rhenium-beryllyl complexes in C–H functionalization chemistry.^10^

Here, we report hydrocarbon metalation catalyzed by manganese—a base metal—and rhenium under photochemical conditions and at atmospheric pressures. Specifically, using catalytic quantities of either CpMn(CO)_3_ (**1**) or **2**, and employing **3** as the beryllium source, the conversion of the unactivated C–H bonds of alkanes (methane) and arenes (benzene) to C–Be and H–Be bonds occurs rapidly. Proposed reaction intermediates, *trans*-CpMn(CO)_2_(BeCp)_2_ (**4**) and *trans*-Cp*Re(CO)_2_(BeCp)_2_ (**5**), were both isolated. Quantum chemical methods have been used to compare the mechanisms of methane beryllation and borylation by **1**. These calculations indicate that the properties of the beryllyl ligands—which are highly electron releasing and Lewis acidic—are key to methane functionalization. Hence, our work provides insights that may inform the future design of functional homogeneous systems for C–H elementation.

Photolysis of an equimolar quantity of either CpMn(CO)_3_ (**1**) or Cp*Re(CO)_3_ (**2**) with CpBeBeCp (**3**) in cyclohexane led to the quantitative formation of *trans*-CpMn(CO)_2_(BeCp)_2_ (**4**) or *trans*-Cp*Re(CO)_2_(BeCp)_2_ (**5**), respectively (Scheme 1).^10,28,30^ In the case of complex **1**, the reaction to form **4** takes only 3 h, while the conversion of **2** to **5** takes longer (ca. 16 h).^30^ Both of the bis(beryllyl) complexes are trivalent, 18-electron, diamagnetic species, and are colorless.

**Scheme 1:**
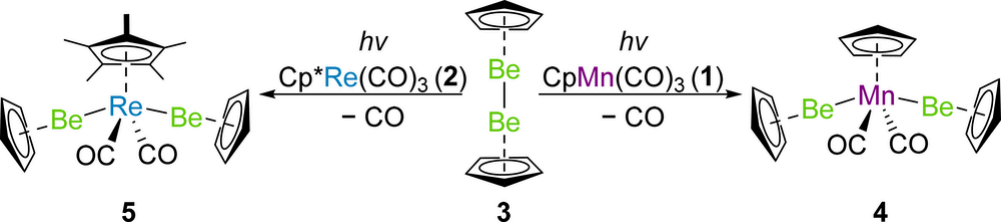
Synthesis of Bis(beryllyl) Complexes 4 and 5

Single crystals of both **4** and **5** could be obtained from concentrated cyclohexane solutions (Figures 1 and S9), allowing for the unambiguous determination of the solid-state structure of each. Both complexes exhibit a four-legged piano-stool geometry, with *trans*-BeCp ligands. The Mn–Be distances in **4** are 2.169(3) and 2.172(3) Å and the Re–Be distances in **5** are 2.298(17) and 2.316(18) Å. Both sets of bond lengths are comparable to the sum of the covalent radii of the respective elements (Mn–Be, 2.21 Å; Re–Be 2.33 Å).^31^

**Figure 1:**
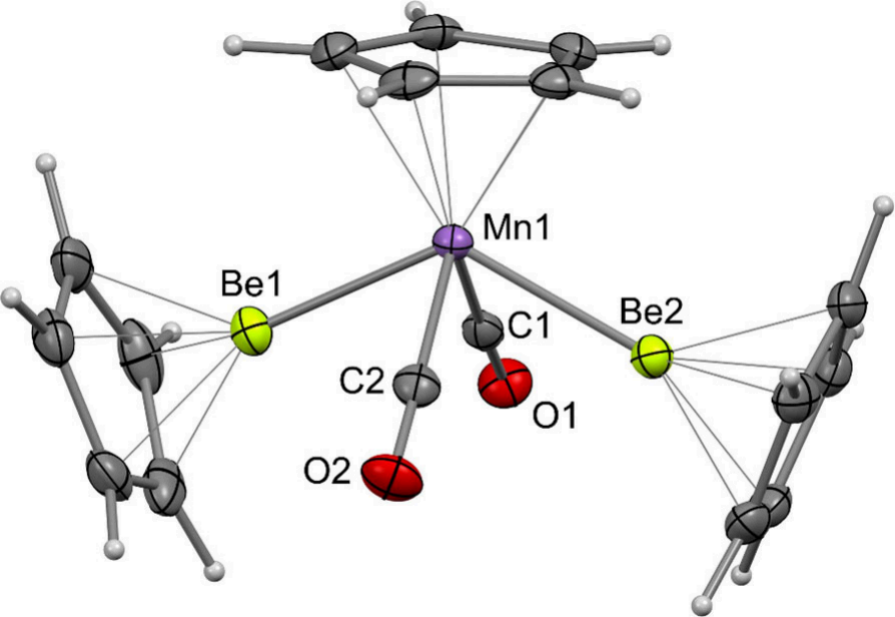
Molecular structure of **4** in the solid state, as determined by X-ray crystallography. Thermal ellipsoids were set at 50% probability.

Cyclohexane-D_12_ solutions of **4** and **5** were also studied by multinuclear NMR spectroscopy. Of greatest interest are the ^9^Be NMR spectra of the complexes, which feature resonances at −12.5 ppm and −10.5 ppm, respectively.^32,33^ These signals are downfield shifted compared to previously disclosed transition metal–beryllyl complexes (e.g., *cis*-Fe(BeCp)_2_(CO)_4_ (−18.0 ppm) and Ni(BeCp)_6_ (−16.7 ppm)).^28,29,34^

In order to better understand the electronic structure of **4** and **5**, we performed quantum chemical calculations on both, as well as their bis(boryl)-analogues, *trans*-CpMn(CO)_2_(BPin)_2_ (**6**) and *trans*-Cp*Re(CO)_2_(BPin)_2_ (**7**) (ωB97X-D4/ZORA-Def2-TZVPP).^10^ The HOMO of both **4** and **5** corresponds to M–CO π-backdonation, combined with a minor M–Be π-bonding component (Figures S10 and S13). The HOMO–1 of both complexes involves a M–CO π-bonding/M–Be σ-bonding combination (Figures S11 and S14).

Natural Bond Orbital (NBO) and Natural Population Analysis (NPA) calculations were performed on complexes **4**–**7**. First-order NBO calculations do not find covalent Mn– or Re–Be interactions. This contrasts with **6** and **7**, for which the M–B bonds are calculated to be composed of approximately 80:20 Re:B and 64:36 Mn:B character, respectively. Second-order NBO calculations indicate that there is substantial π-back-donation from filled Re d-orbitals to Be 2p-orbitals and B–O σ*-antibonding orbitals in **5** and **7**, respectively. However, analogous Mn–Be/B π-back-donation is insignificant in complexes **4** and **6**. According to the Pauling scale of electronegativity (χ: Be, 1.57; Mn, 1.55; Re, 1.90), addition of the Be–Be bond of **3** at manganese is formally an oxidative process, whereas the same reaction should be termed reductive at rhenium. On this basis, complex **4** could be formulated as a manganese(III) species and **5** as a rhenium(−I) complex. However, NPA charges are negative for the d-block metal centers of **4**–**7** (−1.08, −0.68, −0.81, and −0.79, respectively). Thus, the physical oxidation state of manganese in complex **4** is not well represented by the Mn^III^ formalism derived from Pauling electronegativities.^29^ NPA charges at beryllium are similar in both **5** (av. +1.47) and **6** (av. +1.42), and markedly higher than the charges at the boron centers of **6** (av. +1.07) and **7** (av. +1.16).

Quantum Theory of Atoms in Molecules (QTAIM) and Electron Localization Function (ELF) calculations were also performed on complexes **4**–**7**. The calculated Bader charge distributions within these complexes contrast somewhat with those indicated by NPA. The metal centers in **4** and **5** bare charges close to zero (Mn, −0.059; Re, −0.091), while the Mn and Re centers of boryl complexes **6** and **7** are positively charged (Mn, +0.72; Re, +0.92). Bader charges of the Be centers of **4** (av. 1.60) and **5** (av. 1.58), and the B centers of **6** (av. 1.64) and **7** (av. 1.58), are all similar. The parentage of the ELF basins associated with the Mn–Be (75:20, Mn:Be) and Mn–B (30:66, Mn:B) interactions in **4** and **6**, respectively, suggests that these bonds are of opposite polarity to one another. Thus, the computational data indicate that the metal centers in **4** and **5** are more electron rich than those in **6** and **7**, and that the M–Be interactions are polarized to a much greater extent than the analogous M–B bonds (Table S3).

The intermediacy of complex **7** in alkane borylation has been established.^10^ Hence, we were interested to examine whether metal–beryllyl complexes might prove competent in C–H beryllation reactions (Scheme 2).^35^ Accordingly, photolysis of solutions of **3** in C_6_D_6_ with 10 mol % of **1**, **2**, **4**, or **5** forms CpBeD and CpBe(C_6_D_5_), as indicated by multinuclear NMR monitoring (Table S1).^36,37^ Reactions occur at a much faster rate for manganese complexes **1** and **4** (and with lower energy irradiation), presumably due to the greater lability of the carbonyl ligands of these complexes.^30^ The novel complex CpBePh (along with CpBeI) was independently prepared by reaction of **3** with PhI. Notably, the [Pd(PCy_3_)_2_]-catalyzed magnesiation of benzene with [Mg(NacNac^Mes^)]_2_—a complex that features a Mg–Mg bond—has been previously described.^38−40^

**Scheme 2:**
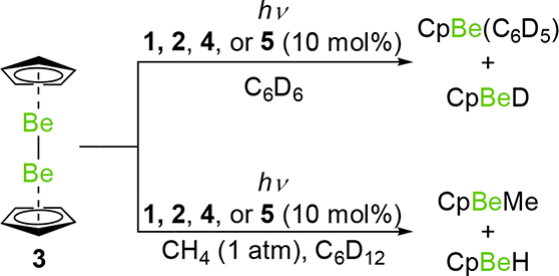
Beryllation of Hydrocarbons with 3 Catalyzed by Group 7 Complexes

Complexes **4** and **5** do not react with the secondary C–H bonds of cyclohexane, similarly to **7**.^10^ Thus, we turned our attention to an alkane comprising only primary C–H bonds: methane. The [Pd(PCy_3_)_2_]-catalyzed C–H magnesiation of methane with [Mg(NacNac^Mes^)]_2_ has not been demonstrated experimentally; the Gibbs free energy of this reaction is calculated to be very close to zero.^38^ However, photolysis of **3** under CH_4_ (1 atm.) in a cyclohexane-D_12_ solution containing 10 mol % of **1** or **2** led to the formation of CpBeMe and CpBeH.^36,37,41,42^ Moreover, **4** and **5** (10 mol %) also conduct this transformation in >75% yield under analogous conditions (Table S1).

Methane borylation catalyzed by complexes **1** and **2** has not been reported.^10,18,20,43^ Moreover, the mechanisms of alkane borylation mediated by these complexes have not been explored in-depth computationally. Thus, we employed quantum chemical calculations to probe the mechanisms of methane borylation (by B_2_Pin_2_) and beryllation (by **3**) catalyzed by **1**, with the aim of discerning and rationalizing the differences between the two reactions. A simplified catalytic cycle for these processes is displayed in Figure 2, with a full reaction profile illustrated in Figure S22.

**Figure 2:**
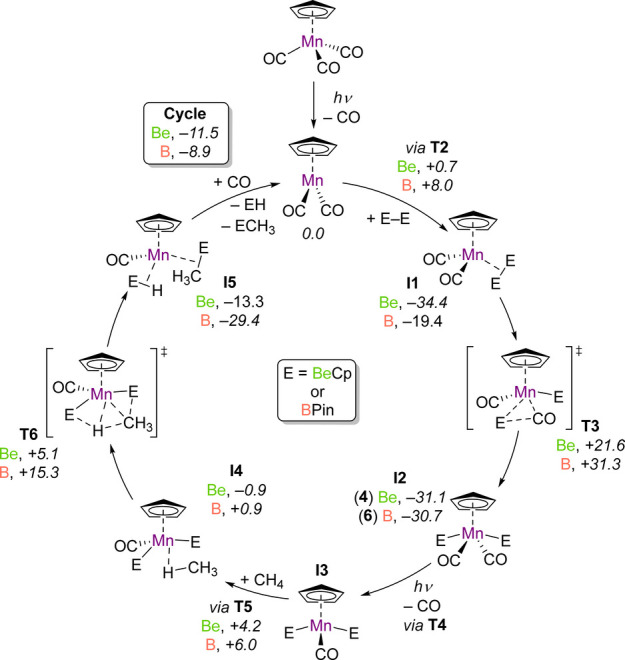
Simplified catalytic cycle for methane functionalization by CpMn(CO)_3_ with either B_2_Pin_2_ or CpBeBeCp. Values in italics correspond to the free energy changes in kcal mol^–1^.

Both mechanisms follow a similar path, beginning with the photoexcitation of **1**, followed by the loss of CO, forming CpMn(CO)_2_. This particular reaction step has been extensively studied by experimental and theoretical methods.^30,44−47^ Subsequent binding of **3** to CpMn(CO)_2_ is essentially barrierless, generating σ-complex **I1Be**, but an activation energy of +8.0 kcal mol^–1^ is calculated for formation of the analogous B_2_Pin_2_ complex, **I1B**. The addition of the Be–Be bond of **3** and B–B bond of B_2_Pin_2_ to Mn occurs in an unusual manner; in both cases a carbonyl ligand assists the process in a fashion that resembles a migratory insertion (**T3**).^48^ The activation energy barrier for this step is much smaller for the generation of **I2Be** (complex **4**) than that for **I2B** (complex **6**) (+21.6 and 31.3 kcal mol^–1^, respectively). Furthermore, while the formation of **I2Be** is exergonic with respect to that of **I1** (−9.5 kcal mol^–1^), that of **I2B** is marginally uphill (+0.6 kcal mol^–1^). We ascribe this to the nature of beryllyl ligands, which are more reducing and Lewis acidic than their boryl analogues, as indicated by QTAIM, NPA, and ELF calculations (*vide supra*).^29^ This is also consistent with experimental work; in our hands complex **6** could not be isolated via photochemical reaction of **1** with B_2_Pin_2_ in cyclohexane.^10^

The next stage of the reaction involves photoinduced CO ejection from **I2**, generating a three-legged piano-stool complex, **I3**, which subsequently binds methane as a C–H σ-complex (**I4**). The next transition state, **T6**, is key and resembles a manganese-assisted concerted double σ-bond metathesis; the C–H bond is broken as E–C and E–H bonds are simultaneously formed.^49,50^ Attempts to optimize the products of methane C–H oxidative addition at Mn were unsuccessful at all relevant points along the reaction coordinates of the borylation and beryllation mechanisms. This is consistent with previous theoretical studies of C–H borylation with iron, rhodium, and tungsten complexes, which suggest that reaction pathways involving C–H oxidative can be much higher in energy than those involving σ-bond metathesis.^11−13^ The activation energy barrier for the formation of **I5Be** from **I4Be** (+5.1 kcal mol^–1^) is notably smaller than that for the analogous formation of **I5B** from **I4B** (+15.3 kcal mol^–1^). Furthermore, **T6B** is located 22.2 kcal mol^–1^ above **I3B**, compared with the +8.4 kcal mol^–1^ energy difference between **I3Be** and **T6Be**. The magnitudes of these energetic barriers provide further evidence for the important role of the X-type boryl or beryllyl ligands in the respective C–H elementation reactions. Indeed, **T6Be** is stabilized by the electron-rich Mn center—a result of the powerful σ-donor properties of the beryllyl ligands—as well as the highly Lewis acidic nature of the beryllium atoms themselves, which polarize the methane C–H bond.^11^ The higher energy of **T6B** is a result of the more covalent Mn–B bonding (Table S3), which renders the boron centers of this transition state less Lewis acidic than the corresponding beryllium sites in **T6Be**, and the Mn atom less electron rich than in the analogous beryllyl transition state.

Finally, the newly formed element-hydride dissociates (**T7**) and CO recoordinates to Mn (**T8**), before dissociation of the E–CH_3_ species from the metal center, regenerating CpMn(CO)_2_ (**T9**).^51^ Overall, the catalytic cycle for methane borylation by **1** is slightly less exergonic than beryllation (−8.9 versus −11.6 kcal mol^–1^).

In conclusion, under photochemical conditions, the catalytic beryllation of methane and benzene by CpBeBeCp (**3**) has been achieved using CpMn(CO)_3_ (**1**) or Cp*Re(CO)_3_ (**2**). Viable intermediates in hydrocarbon beryllation—*trans*-CpMn(CO)_2_(BeCp)_2_ (**4**) and *trans*-Cp*Re(CO)_2_(BeCp)_2_ (**5**)—have been isolated. Inspection of the mechanisms of methane borylation and berylation by **1** reveals that the properties of the beryllyl ligands facilitate this novel C–H elementation reaction to an extent that is not possible with boron analogues.

## Supporting Information Available

Materials and methods, experimental procedures, spectroscopic data, crystallographic details, and quantum chemical data (PDF)
