# Supplementary material for: Methane Beryllation Catalyzed by a Base Metal Complex
Source: J Am Chem Soc. 2025 Mar 11;147(12):10073–7. doi: 10.1021/jacs.5c02179 (PMC11951138; doi:10.1021/jacs.5c02179)
Supplement: Supplementary file 1 — ja5c02179_si_001.pdf [file ja5c02179_si_001.pdf]

**Supplementary Materials**

# Methane Beryllation Catalysed by a Base Metal Complex

Josef T. Boronski*[1], Agamemnon E. Crumpton[2], Job J. C. Struijs[2], Simon Aldridge*[2]

1) Department of Chemistry, Molecular Sciences Research Hub, Imperial College London, White City, London, W12 7TA, U.K.

2) Department of Chemistry, Chemistry Research Laboratory, University of Oxford, Oxford, OX1 3TA, U.K.

**Contents**

**Experimental Considerations** – S2

**Synthesis of Novel Compound and Reactivity Studies** – S2

**Spectroscopic Data** – S5

**Crystallographic Data** – S9

**Computational Details** – S11

Molecular Orbitals – S12

Quantum Theory of Atoms in Molecules Topological Analysis – S17

Mechanistic Studies – S18

Coordinates of Optimised Structures – S19

**References** – S31

**Experimental Considerations:**

All manipulations were carried out using Schlenk line or glovebox techniques under an atmosphere of argon or dinitrogen. Solvents were dried by passage through activated alumina towers, dried with $NaK_2$ and degassed before use. Solvents were stored over $NaK_2$. NMR spectra were measured in $C_6D_{12}$ and $C_6D_6$ which were dried over $NaK_2$, with the solvent being distilled under reduced pressure, degassed by three freeze-pump-thaw-cycles and stored under argon in a Teflon valve ampoule. NMR samples were prepared under argon in 5 mm Wilmad 507-PP tubes fitted with J. Young Teflon valves. NMR spectra were measured on a Bruker Avance III HD Nanobay 400 MHz NMR spectrometer equipped with a 9.4 T magnet or a Bruker Avance III NMR 500 MHz NMR spectrometer equipped with a 11.75 T magnet and a $^{13}C$ detect cryoprobe. $^{1}H$ and $^{13}C\{^{1}H\}$ NMR spectra were referenced internally to residual protio-solvent ($^{1}H$) or solvent ($^{13}C$) resonances and are reported relative to tetramethylsilane ($\delta$ = 0 ppm). $^{9}Be$ NMR spectra were referenced to a 0.43 M solution of $BeSO_4 \cdot 4H_2O$ in $D_2O$ ($\delta$ = 0 ppm). Chemical shifts are quoted in $\delta$ (ppm) and coupling constants in Hz. FTIR spectra were recorded on a Bruker Alpha spectrometer with Platinum-ATR module. $CpMn(CO)_3$,[1] $Cp^*Re(CO)_3$,[2] and Diberyllocene was prepared as described previously.[3] Methane was purchased from Sigma Aldrich and stored over molecular sieves for 48 hours before use.

**Health warning:** beryllium and its compounds are extremely toxic and can cause irreversible health effects through inhalation or skin contact. The work with beryllium-containing materials described herein was carried out by trained operator(s), with strict adherence to local and national rules/regulations.[4] The toxicity of the beryllium-containing compounds precludes their characterization by elemental analysis and mass spectrometry.

**Synthesis of Novel Compounds and Reactivity Studies:**

***Synthesis of trans-CpMn(BeCp)$_2$(CO)$_2$ (4):*** To a J Young NMR tube was added a solid mixture of diberyllocene (7.0 mg, 0.047 mmol) and $CpMn(CO)_3$ (9.6 mg, 0.047 mmol). Cyclohexane (0.5 mL) was condensed into the vessel *in vacuo* at –196 °C. The yellow solution was allowed to warm to room temperature and irradiated ($\lambda$ = 405 nm, 50W LED lamp) for 3 hours. Volatiles were removed *in vacuo*, yielding an off-white solid. Soluble material was extracted with cyclohexane (1 mL) and the solution was filtered. The pale-yellow solution was transferred to a λ-crystallization tube (fitted with a J Young PTFE valve), frozen, and placed under a static vacuum. Slow concentration of the solution over the course of 16 hours led to the formation of a crop of colourless crystals of **4**, which were dried *in vacuo*. Yield: 11.0 mg, 72%. Single crystals of **4** suitable for X-ray diffraction experiments were obtained by slow concentration of a cyclohexane solution in a λ-crystallization tube. $^{1}H$ NMR (400 MHz, $C_6D_{12}$,

298 K): δ = 6.14 (s, 10H, Be($C_5$*$H_5$*)), 3.86 (s, 5H, Mn($C_5$*$H_5$*)); $^{9}$Be NMR (42 MHz, $C_6D_{12}$): δ = –12.5 ($w_{1/2}$ = 10.6 Hz); $^{13}$C{$^{1}$H} NMR (101 MHz, $C_6D_{12}$): δ = 105.7 (Be(*$C_5$*$H_5$)), 78.8 (Mn(*$C_5$*$H_5$)). ATR-IR *υ*/cm$^{-1}$: 520 (w), 614 (s), 671 (s), 789 (s), 855 (m), 1006 (s), 1258 (w), 1424 (w), 1790 (s), 1858 (s), 2836 (w), 2905 (w), 2945 (w), 3094 (w).

***Synthesis of trans-Cp*Re(BeCp)$_2$(CO)$_2$ (5):*** To a J Young NMR tube was added a solid mixture of diberyllocene (7.0 mg, 0.047 mmol) and Cp*Re(CO)$_3$ (19.2 mg, 0.047 mmol). Cyclohexane (0.5 mL) was condensed into the vessel *in vacuo* at –196 °C. The colourless solution was allowed to warm to room temperature and irradiated (75W mercury arc lamp) for 16 hours. Volatiles were removed *in vacuo*, yielding a white solid. Soluble material was extracted with cyclohexane (1 mL) and the solution was filtered. The colourless solution was transferred to a λ-crystallization tube (fitted with a J Young PTFE valve), frozen, and placed under a static vacuum. Slow concentration of the solution over the course of 16 hours led to the formation of a crop of colourless crystals of **5**, which were dried *in vacuo*. Yield: 14.9 mg, 60%. Single crystals of **5** suitable for X-ray diffraction experiments were obtained by slow concentration of a cyclohexane solution in a λ-crystallization tube. $^{1}$H NMR (400 MHz, $C_6D_{12}$, 298 K): δ = 6.12 (s, 10H, $C_5$*$H_5$*), 1.91 (s, 15H, $C_5$(C*$H_3$*)$_5$); $^{9}$Be NMR (42 MHz, $C_6D_{12}$): δ = –10.5 ($w_{1/2}$ = 16.2 Hz); $^{13}$C{$^{1}$H} NMR (101 MHz, $C_6D_{12}$): δ = 104.5 (Be(*$C_5$*$H_5$)), 94.2 (*$C_5$*($CH_3$)$_5$), 11.9 ($C_5$(*C*$H_3$)$_5$). ATR-IR *υ*/cm$^{-1}$: 395 (w), 426 (w), 512 (m), 580 (m), 609 (w), 680 (w), 769 (s), 929 (m), 1252 (w), 1370 (m), 1444 (w), 1470 (w), 1796 (s), 1867 (s), 2836 (w), 2905 (w), 2942 (w).

***Procedure for Catalytic Benzene Beryllation with Complexes 1, 2, 4, and 5:*** To a J Young NMR tube was added a solid mixture of diberyllocene (5.0 mg, 0.034 mmol) and **1**, **2**, **4**, or **5** (10 mol%). Benzene (0.5 mL) was condensed into the vessel *in vacuo* at –196 °C. The solution was allowed to warm to room temperature and irradiated (**1**, **4**: λ = 405 nm, 50W LED lamp, 3 hours; **2**, **5**: 75W mercury arc lamp, 20 hours). Yields were determined by $^{1}$H NMR spectroscopy (cyclohexane internal standard capillary). **Spectroscopic Data, CpBeD**: $^{1}$H NMR (400 MHz, $C_6D_6$, 298 K): δ = 5.69 (s, 5H, $C_5$*$H_5$*); $^{9}$Be NMR (42 MHz, $C_6D_6$): δ = –20.7 ($w_{1/2}$ = 9.3 Hz).[5,6]

***Independent Synthesis of CpBePh:*** To a J Young NMR tube was added diberyllocene (5.0 mg, 0.034 mmol). Benzene (0.5 mL), then iodobenzene (0.1 mL) were condensed into the vessel *in vacuo* at –196 °C. The mixture was allowed to warm to room temperature, then heated overnight at 80 °C. **Spectroscopic Data, CpBePh**: $^{1}$H NMR (400 MHz, $C_6D_6$, 298 K): δ = 7.44 (d, 2H, *m*-$C_6$*$H_5$*), 7.29 (m, 3H, *o*- and *p*-$C_6H_5$), 5.77 (s, 5H, $C_5$*$H_5$*); $^{9}$Be NMR (42 MHz, $C_6D_6$): δ = –19.7 ($w_{1/2}$ = 25.0 Hz). **Spectroscopic Data, CpBeI**: $^{1}$H NMR (400 MHz, $C_6D_6$, 298 K): δ = 5.58 (s, 5H, $C_5$*$H_5$*); $^{9}$Be NMR (42 MHz, $C_6D_6$): δ = –20.3 ($w_{1/2}$ = 6.9 Hz).

***Procedure for Catalytic Methane Beryllation with Complexes 1, 2, 4, and 5:*** To a J Young NMR tube was added a solid mixture of diberyllocene (5.0 mg, 0.034 mmol) and **1**, **2**, **4**, or **5** (10 mol%). Cyclohexane (0.5 mL) was condensed into the vessel *in vacuo* at –196 °C. The solution was then degassed (3 x freeze-pump-thaw cycles) and an atmosphere of methane was introduced. The solution was allowed to warm to room temperature and irradiated (**1**, **4**: λ = 405 nm, 50W LED lamp, 3 hours; **2**, **5**: 75W mercury arc lamp, 20 hours). Yields were determined by $^1$H NMR spectroscopy (benzene internal standard capillary). Spectroscopic data are consistent with those previously reported for CpBeMe.[7,8]

***Table S1***: Yields of hydrocarbon beryllation reactions with **1**, **2**, **4**, and **5**.

| **Substrate** | **Complex** (amount / %) | **Conversion of CpBeBeCp** / % | **Yield CpBeH** / % | **Yield CpBeR** / % (R = Ph or Me) |
|---|---|---|---|---|
| $C_6D_6$ | **1** (10) | 82 | 53 | 59 |
| $C_6D_6$ | **2** (10) | 71 | 55 | 60 |
| $C_6D_6$ | **4** (10) | 82 | 72 | 73 |
| $C_6D_6$ | **5** (10) | 76 | 63 | 64 |
| $CH_4$ | **1** (10) | 100 | 84 | 86 |
| $CH_4$ | **2** (10) | 70 | 65 | 65 |
| $CH_4$ | **4** (10) | 98 | 86 | 89 |
| $CH_4$ | **5** (10) | 82 | 75 | 79 |

As outlined in greater detail above, all $CH_4$ beryllations were conducted at 1 atm. gas pressure. In the case of complexes **1** and **4**, reaction times were three hours and a 456 nm, 50W LED lamp was used. In the case of complexes **2** and **5**, reactions were 20 hours and a 75W mercury arc lamp was used.

**Spectroscopic Data:**

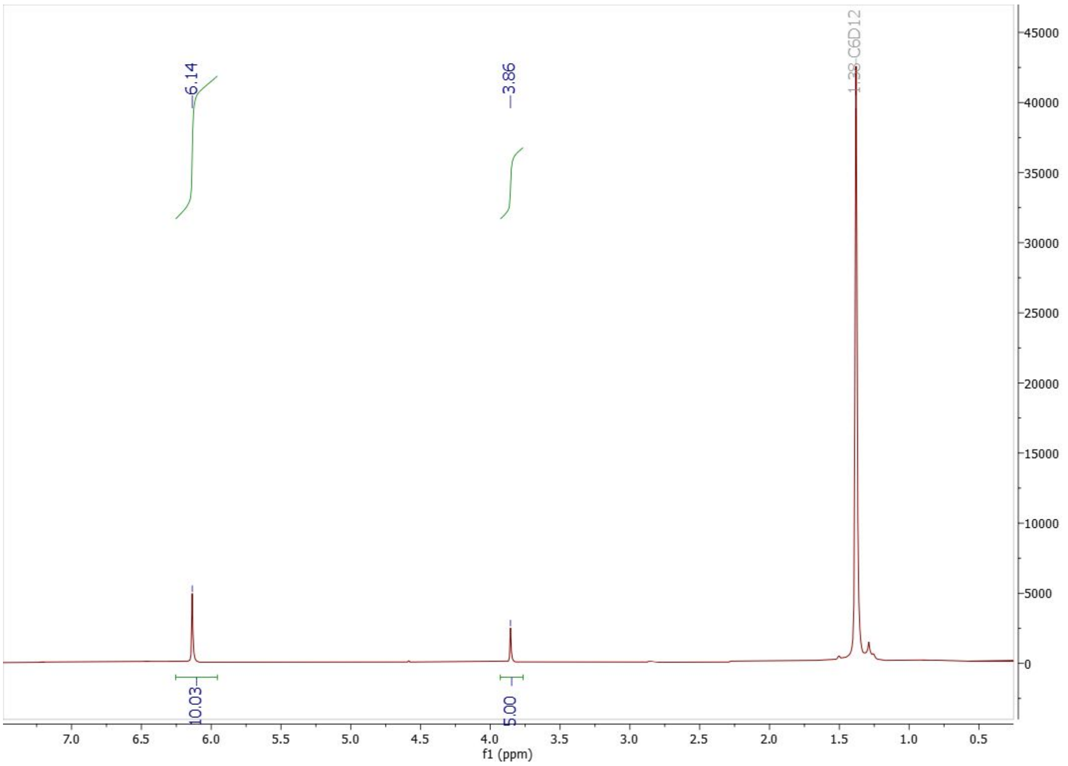


Figure S1: $^{1}H$ NMR spectrum of **4** in $C_6D_{12}$.

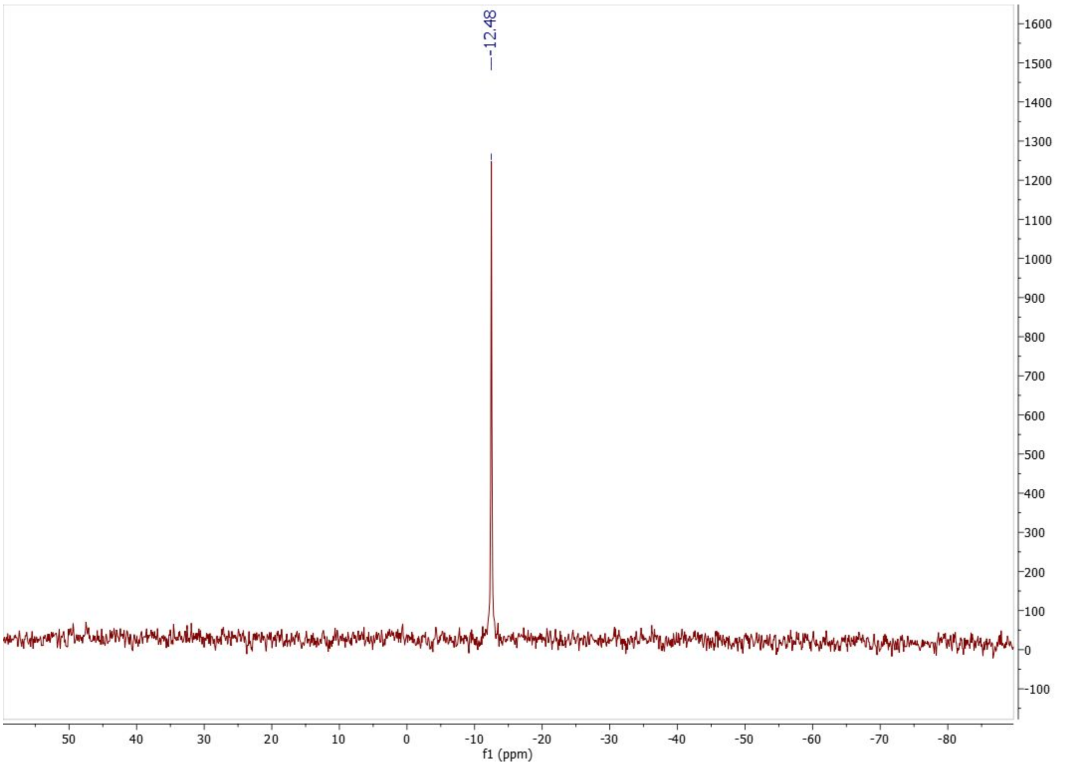


Figure S2: $^{9}Be$ NMR spectrum of **4** in $C_6D_{12}$.

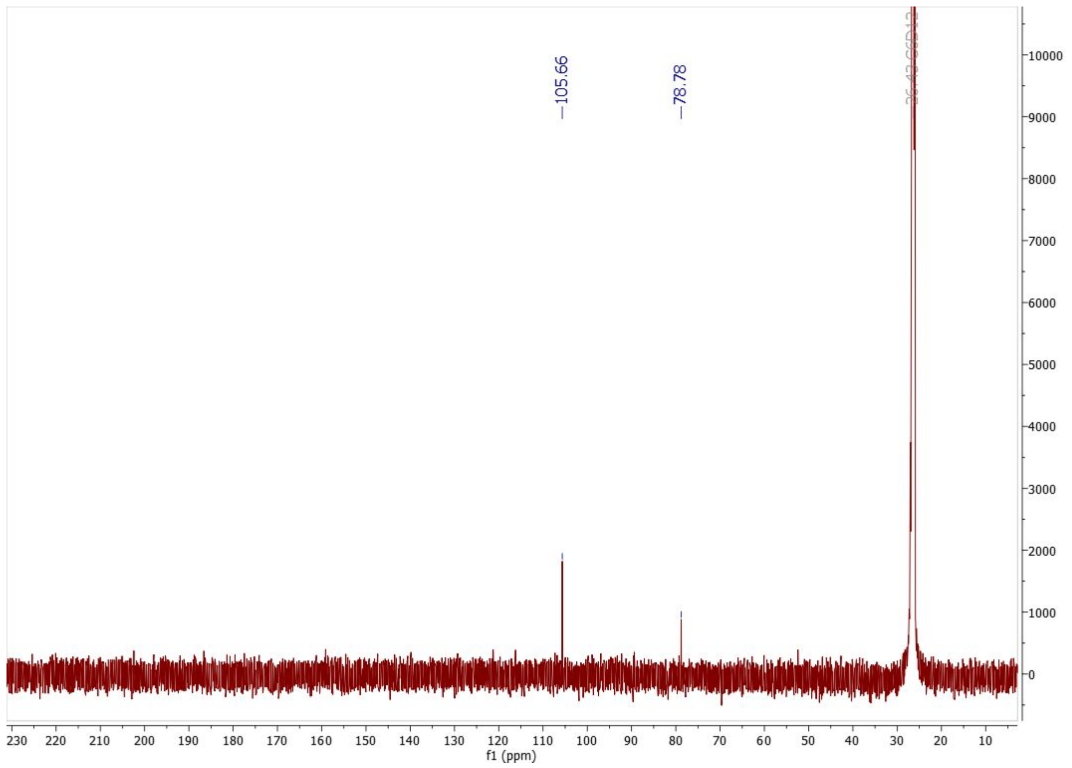


Figure S3: $^{13}C\{^1H\}$ NMR spectrum of **4** in $C_6D_{12}$.

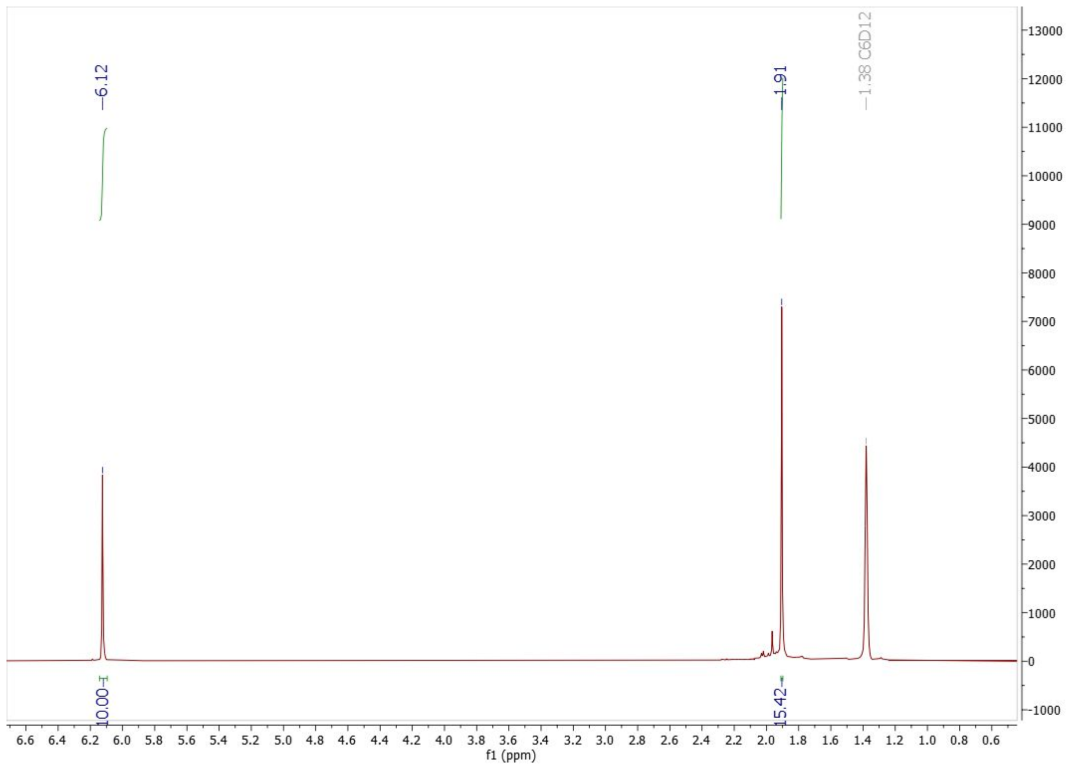


Figure S4: $^1H$ NMR spectrum of **5** in $C_6D_{12}$.

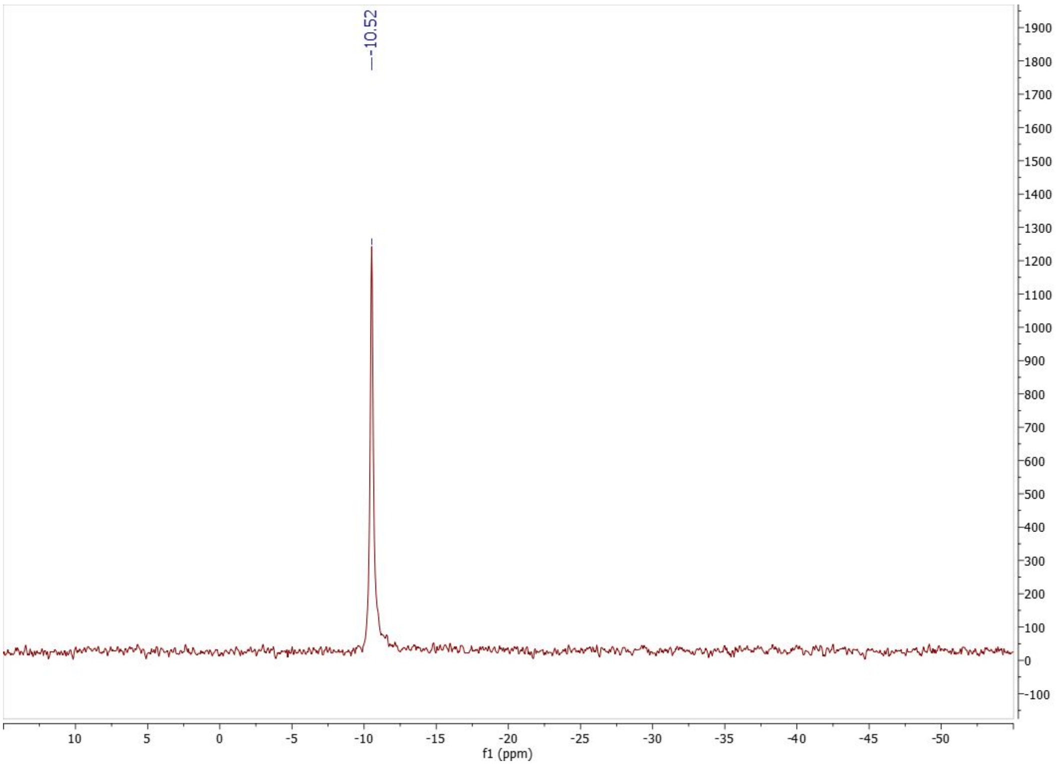


Figure S5: $^{9}Be$ NMR spectrum of **5** in $C_6D_{12}$.

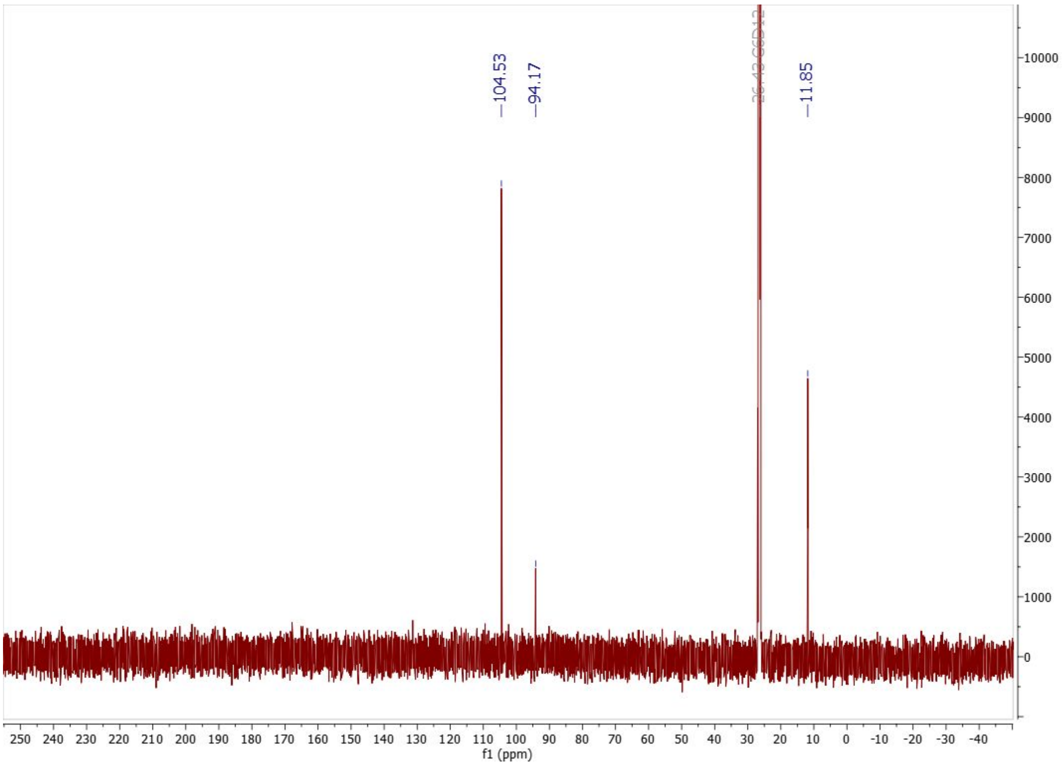


Figure S6: $^{13}C\{^{1}H\}$ NMR spectrum of **5** in $C_6D_{12}$.

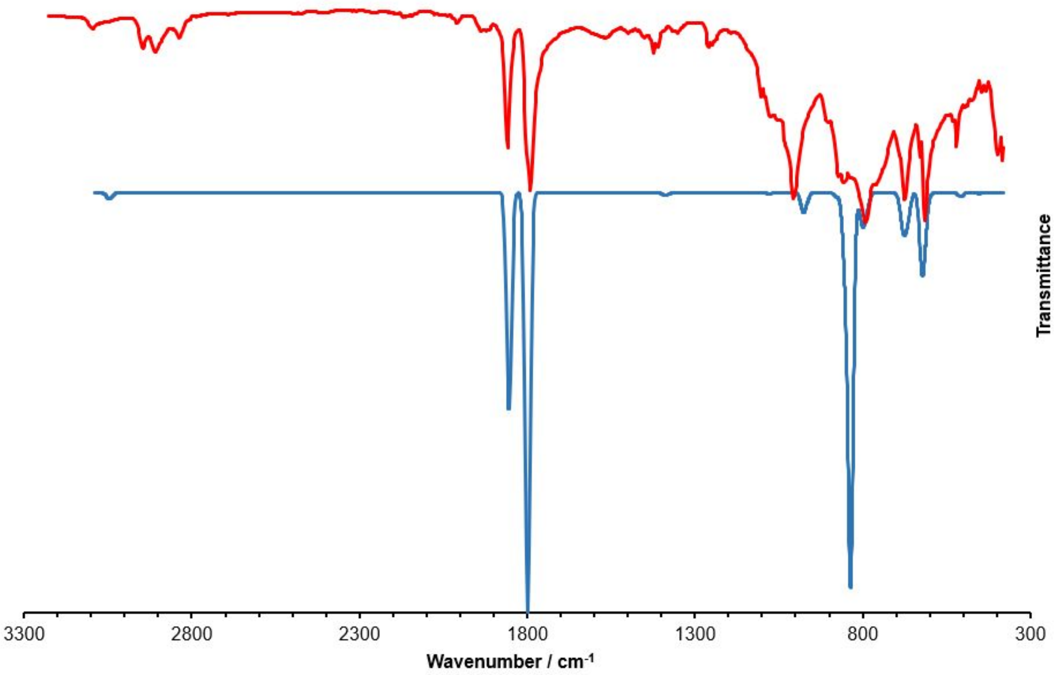


Figure S7: ATR IR spectrum of **4** (red = experimental; blue = computationally simulated).

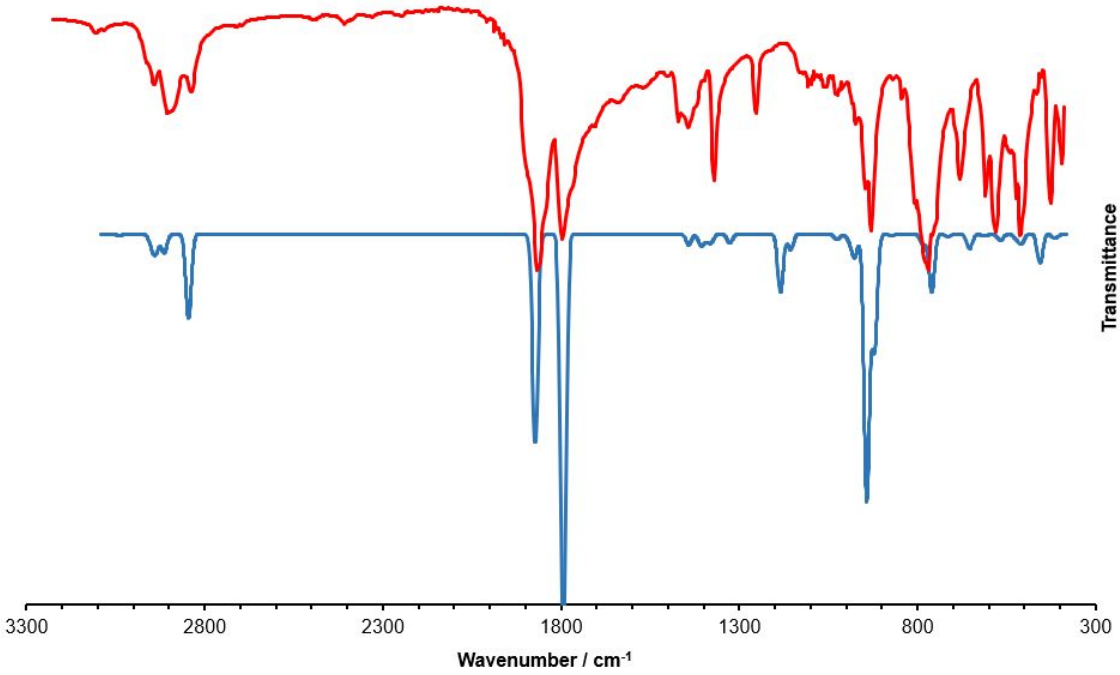


Figure S8: ATR IR spectrum of **5** (red = experimental; blue = computationally simulated).

**Crystallographic Data:**

Data for **4** and **5** were collected using an Oxford Diffraction/Agilent SuperNova or Rigaku XtaLAB Synergy-R. Crystals were selected under Paratone-N or perfluorinated oil, mounted on MiTeGen Micromount loops and quench-cooled using an Oxford Cryosystems open flow $N_2$ cooling device.[9] Selected details of data collection are given in Table S2. Data collected were processed using the CrysAlisPro package, including unit cell parameter refinement and inter-frame scaling (which was carried out using SCALE3 ABSPACK within CrysAlisPro).[10] Equivalent reflections were merged and diffraction patterns processed with the CrysAlisPro suite. Structures were solved ab initio from the integrated intensities using SHELXT and refined on F2 using SHELXL with the graphical interface OLEX2.[11–13] Crystallographic data is given in the supplementary deposited CIF files (CCDC 2415852, 2415853) and can be obtained free of charge from the Cambridge Crystallographic Data Centre via http://www.ccdc.cam.ac.uk/data_request/cif.

Table S2: Crystallographic details for complexes **4** and **5**.

| | **4** | **5** |
|---|---|---|
| Formula | C17 H15 Be2 Mn O2 | C22 H25 Be2 O2 Re |
| Fw (g mol$^{-1}$) | 324.25 | 525.64 |
| Cell setting | orthorhombic | orthorhombic |
| Space group | $P2_12_12_1$ | $Pnma$ |
| *a* (Å) | 8.49260(10) | 16.8166(3) |
| *b* (Å) | 10.78400(10) | 10.5473(2) |
| *c* (Å) | 16.27450(10) | 11.5625(3) |
| *α* (°) | 90 | 90 |
| *β* (°) | 90 | 90 |
| *γ* (°) | 90 | 90 |
| *V* (Å$^{-3}$) | 1490.49(2) | 2050.83(8) |
| *Z* | 4 | 4 |
| $\rho_{calc}$ (g cm$^{-3}$) | 1.445 | 1.702 |
| Radiation, λ (Å) | 1.54184 | 1.54184 |
| μ (mm$^{-1}$) | 7.187 | 11.670 |
| $R_{(int)}$ | 0.0359 | 0.0907 |
| Parameters | 199 | 213 |
| $R_1$ | 0.0243 | 0.0499 |
| $\omega R_2$ | 0.0600 | 0.1297 |
| GooF | 1.068 | 1.076 |
| *T* (K) | 100.00(10) | 100.0(2) |
| CCDC Deposition No. | 2415852 | 2415853 |

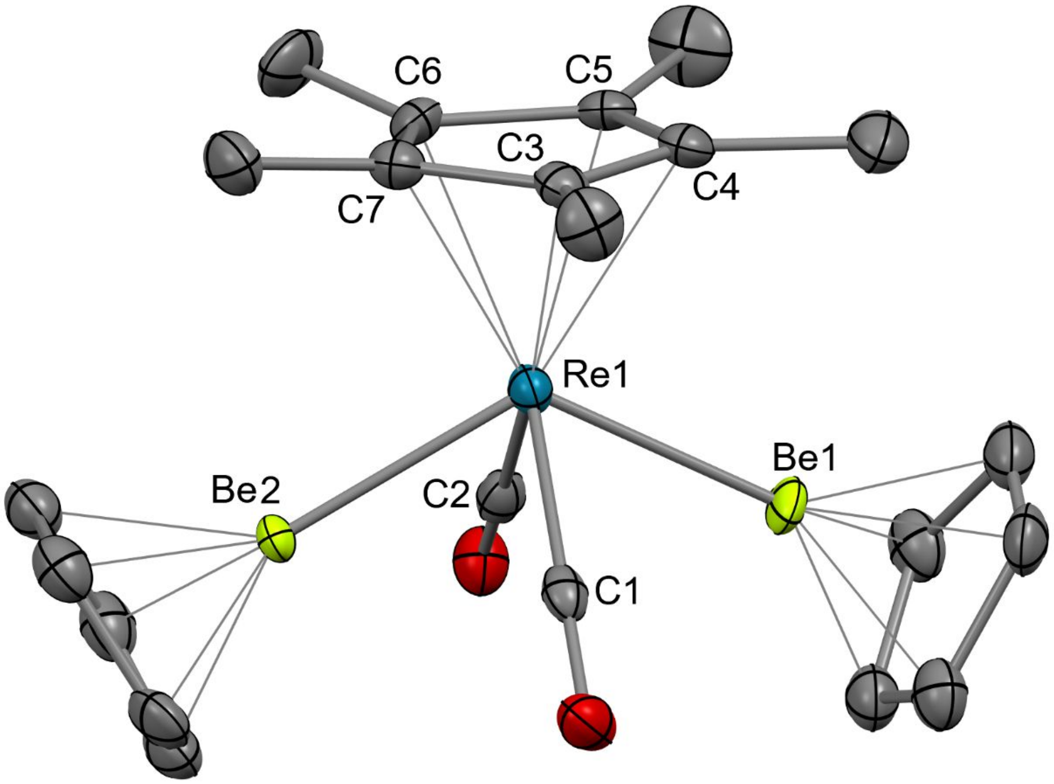


Figure S9: Molecular structure of **5** in the solid state as determined by X-ray crystallography. Thermal ellipsoids set at 50% probability and hydrogen atoms omitted for clarity.

**Computational Details:**

The structures of complexes **4** – **7**, and all reaction intermediates/transition states (**1**, **S1**, **ST1**, **CpMn(CO)$_2$**, **T2Be** – **T9Be**, **I1Be** – **I7Be**, **T2B** – **T9B**, and **I1B** – **I7B**) were optimised using ORCA (Revision 6.0.0).[14,15] Specifically, structures were optimised with the ωB97X range-separated hybrid functional and zeroth order relativistic approximation (ZORA), in conjunction with the all-electron ZORA-Def2-TZVPP basis set, reparametrized D4 dispersion correction, and CPCM solvent (benzene) modelling.[16–19] The nature of the stationary points (minima) was confirmed by full frequency calculations, and are characterized by zero imaginary frequencies. The nature of transition states was confirmed by full frequency calculations, and are characterized by the presence of a single imaginary frequency corresponding to the reaction coordinate. Natural Bond Orbital and Natural Population Analysis calculations were performed on the ORCA wavefunction using NBO 7.0.[20] Quantum Theory of Atoms in Molecules calculations were performed using the ORCA wavefunctions for the respective complexes, and were generated using Multiwfn 3.8.[21] Electron Localisation Function calculations were performed using the ORCA wavefunctions for the respective complexes, generated using Multiwfn 3.8.

Triplet species have been postulated for the conversion of **1** to CpMn(CO)$_2$.[22,23] Thus, the possibility of both singlet and triplet intermediates/transition states was examined during mechanistic study. However, it was found that the singlet-triplet gaps of all relevant species are >25 kcal mol$^{-1}$. This effectively rules out the role of triplet species in this mechanism.

Selected Molecular Orbitals of Complexes **4** – **7**:

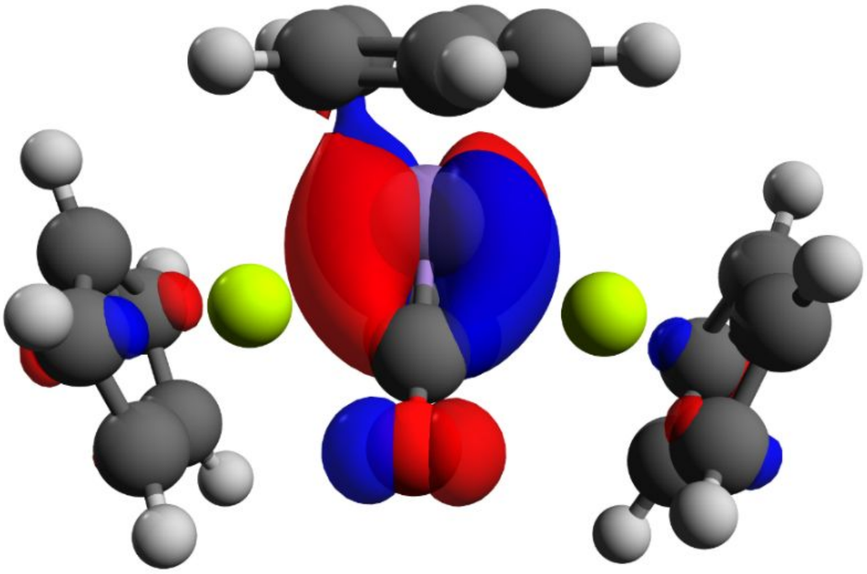

Figure S10: HOMO of complex **4**.

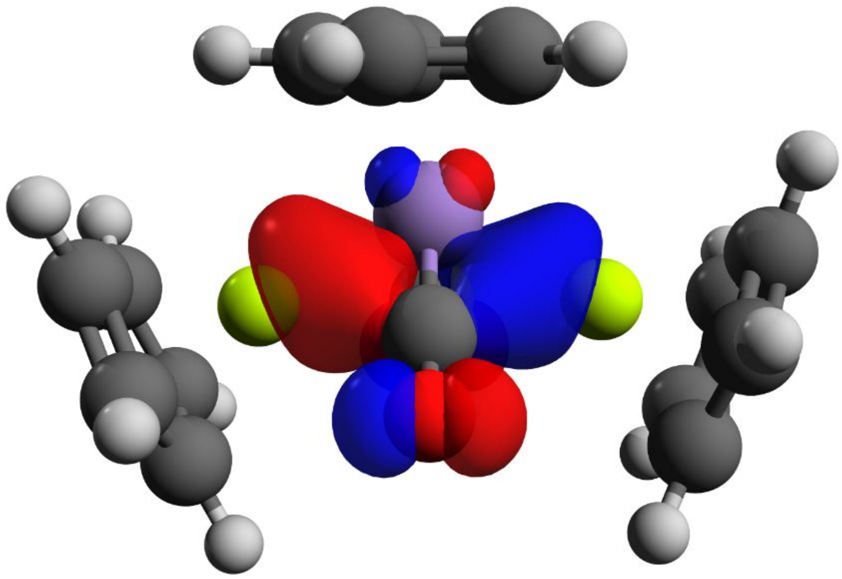

Figure S11: HOMO–1 of complex **4**.

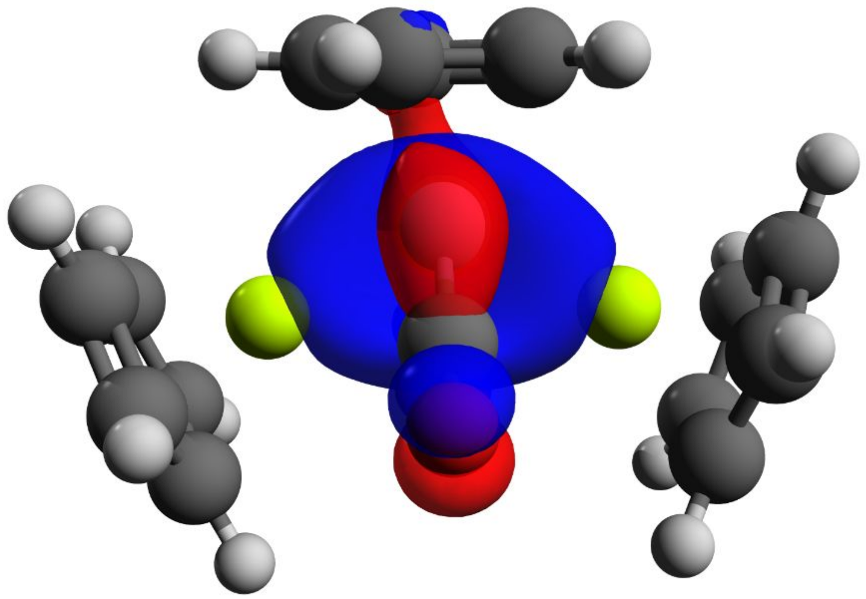

Figure S12: HOMO–3 of complex **4**.

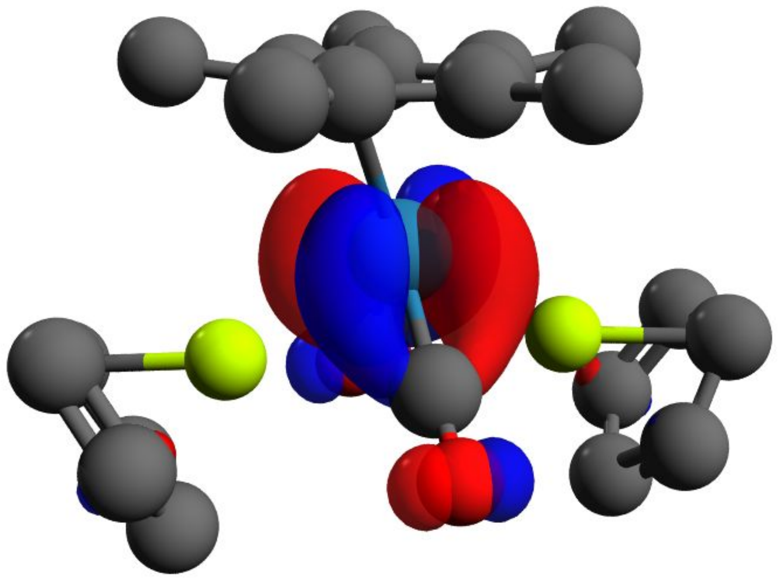

Figure S13: HOMO of complex **5**.

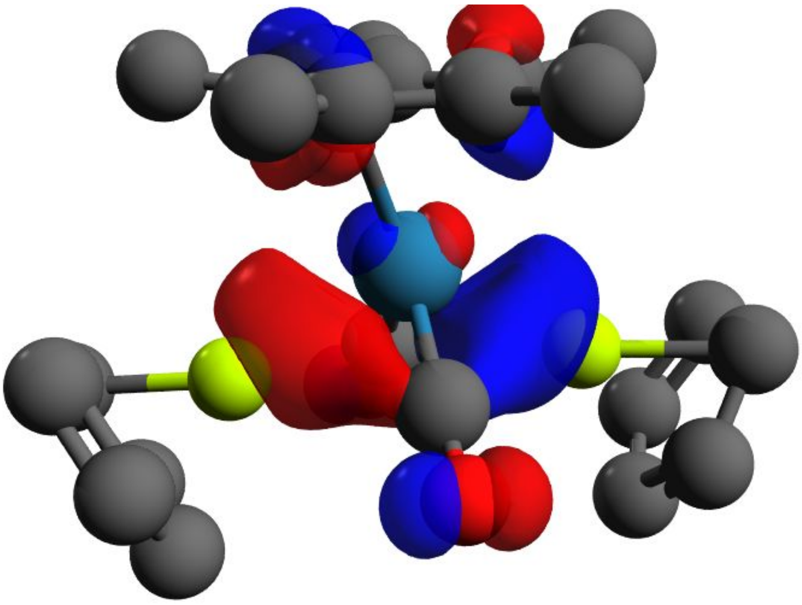

Figures S14: HOMO–1 of complex **5**.

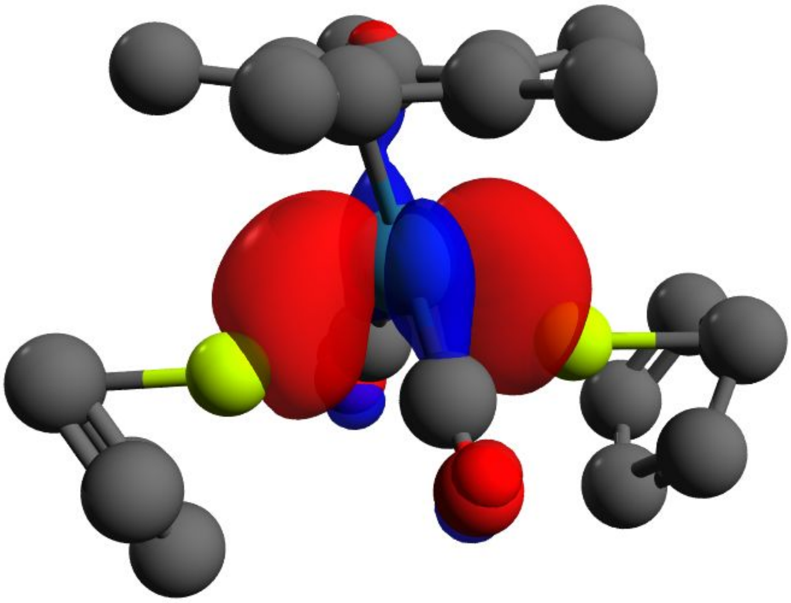

Figure S15: HOMO–3 of complex **5**.

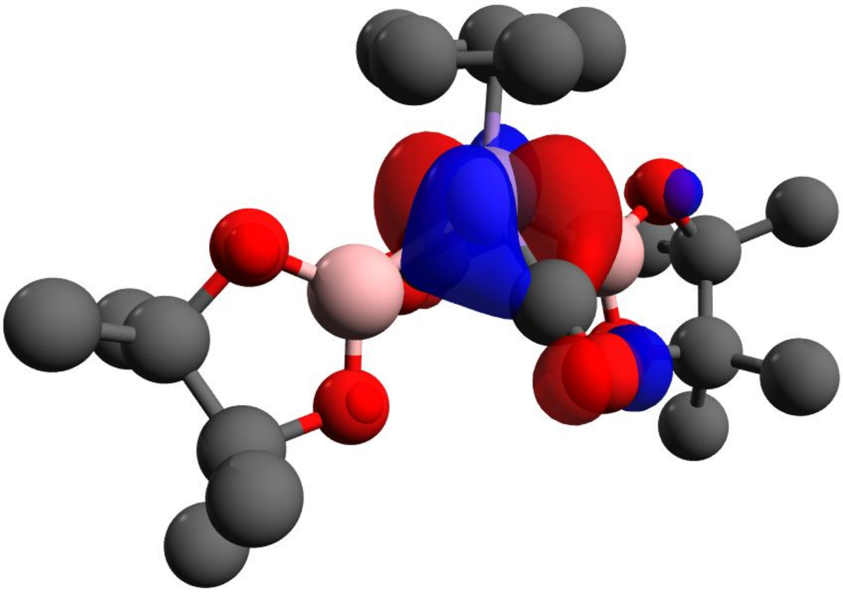

Figure S16: HOMO of complex **6**.

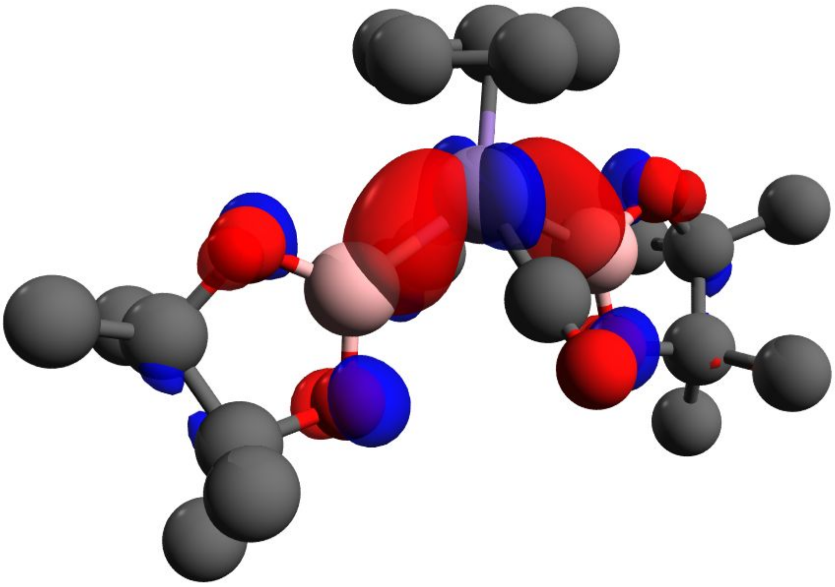

Figure S18: HOMO–3 of complex **6**.

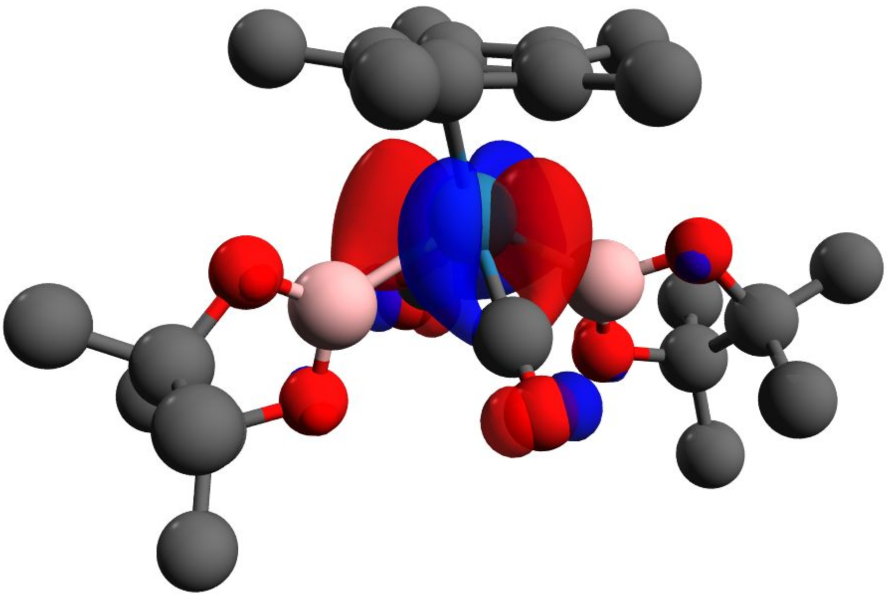

Figure S19: HOMO of complex **7**.

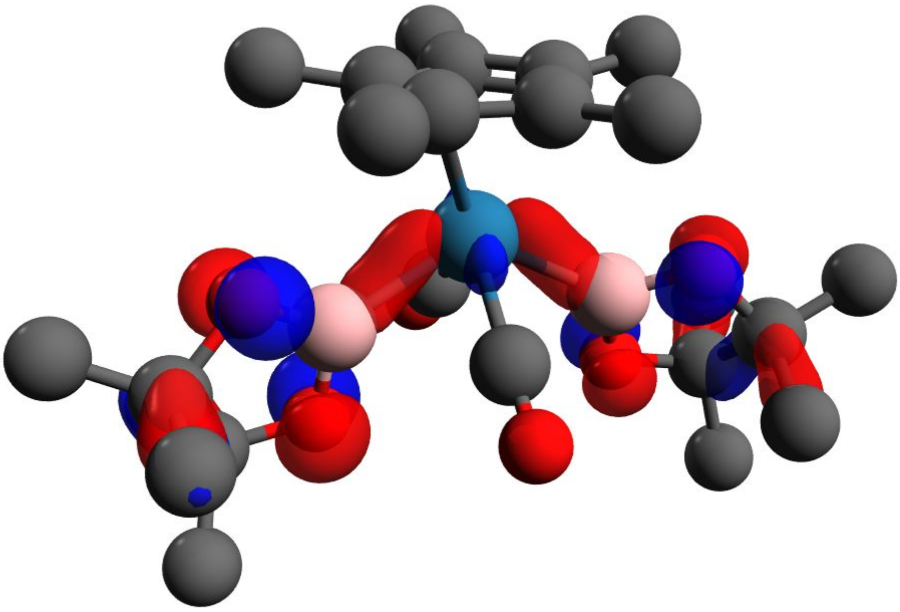

Figure S20: HOMO–4 of complex **7**.

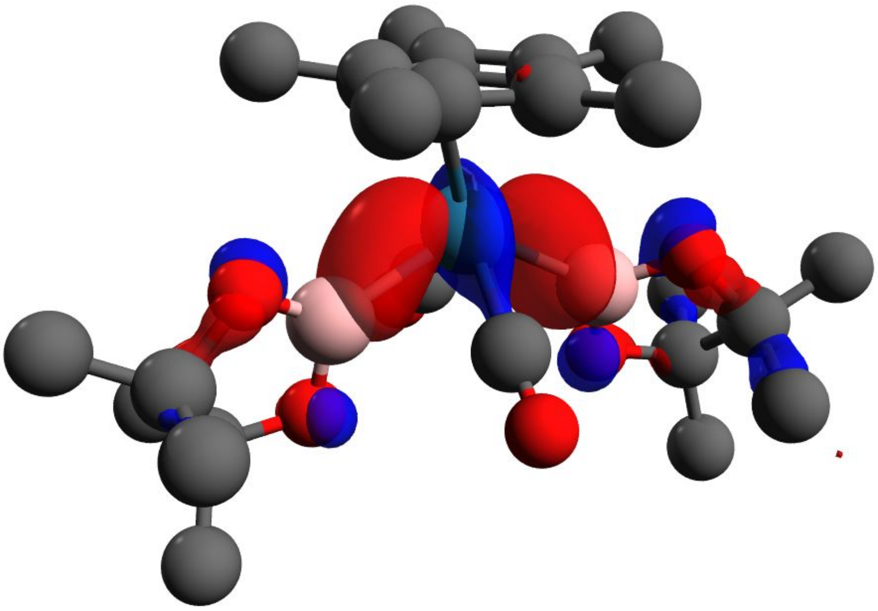

Figure S21: HOMO–6 of complex **7**.

Quantum Theory of Atoms in Molecules Calculations:

Table S3: Summary of QTAIM topological data generated for **4 – 7**. All values in a.u., unless otherwise stated.

| | **4** | | **5** | | **6** | | **7** | |
|---|---|---|---|---|---|---|---|---|
| **Critical Point** | Be1-Mn | Be2-Mn | Be1-Re | Be2-Re | B1-Mn | B2-Mn | B1-Re | B2-Re |
| **$\rho_{bcp}$ / e⁻ Bohr⁻³** | **0.058** | **0.058** | **0.061** | **0.061** | **0.104** | **0.104** | **0.111** | **0.112** |
| **KE (L)** | 0.038 | 0.037 | 0.038 | 0.039 | 0.046 | 0.046 | 0.041 | 0.040 |
| **KE (H)** | 0.028 | 0.028 | 0.030 | 0.030 | 0.054 | 0.054 | 0.058 | 0.059 |
| **V** | -0.066 | -0.065 | -0.069 | -0.069 | -0.100 | -0.100 | -0.100 | -0.099 |
| **E** | -0.028 | -0.028 | -0.030 | -0.030 | -0.054 | -0.054 | -0.058 | -0.059 |
| **$\nabla^2\rho_{bcp}$ / e⁻ Bohr⁻⁵** | **0.037** | **0.033** | **0.033** | **0.033** | **-0.032** | **-0.031** | **-0.063** | **-0.069** |
| **ELF** | 0.307 | 0.315 | 0.334 | 0.332 | 0.672 | 0.671 | 0.760 | 0.771 |
| **LOL** | 0.399 | 0.404 | 0.414 | 0.413 | 0.589 | 0.588 | 0.641 | 0.647 |

Mechanistic Studies:

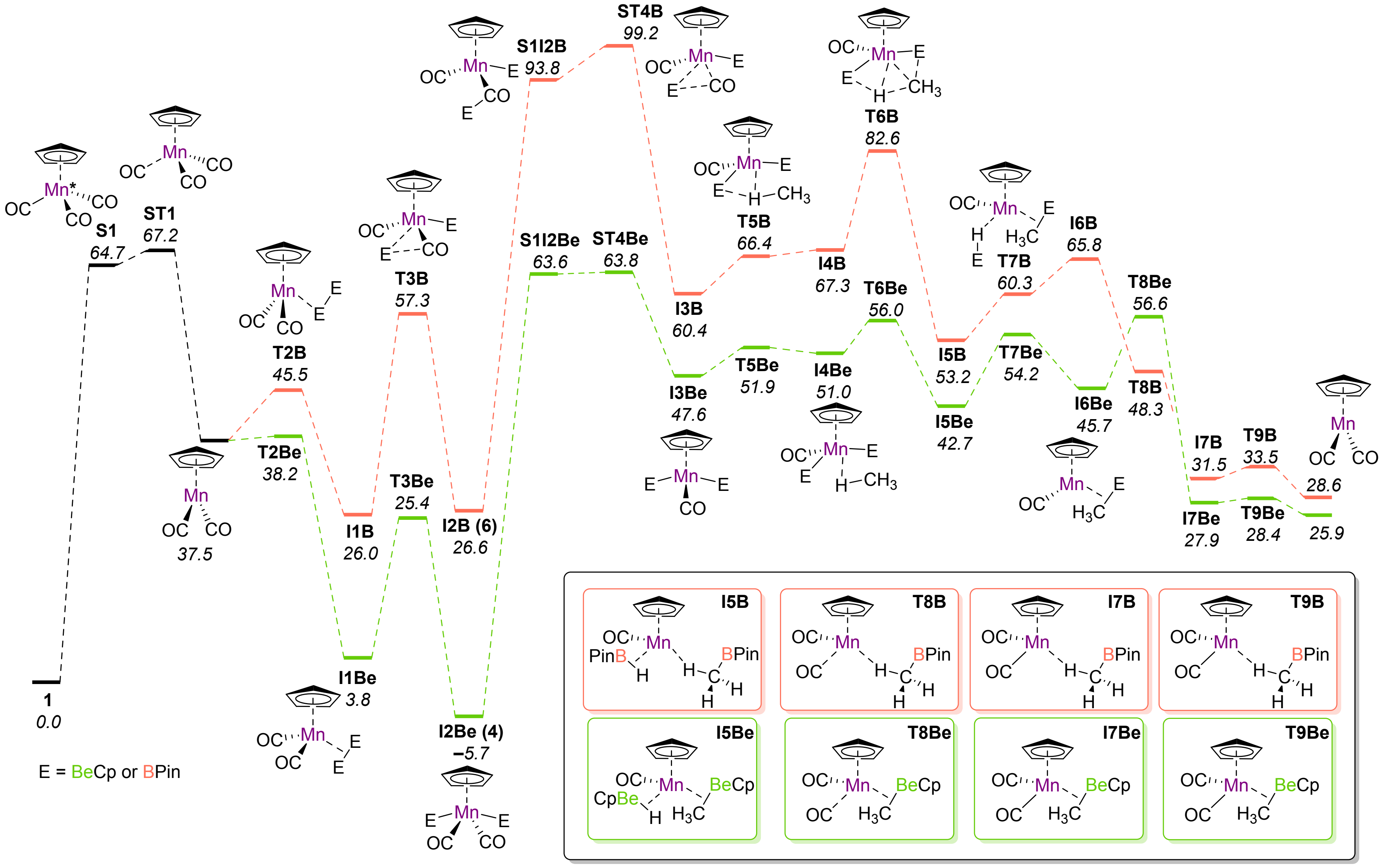


Figure S22: Proposed mechanisms for methane borylation and beryllation (ωB97X/ZORA-Def2-TZVPP). Peach-coloured pathway represents borylation reaction; lime-green pathway represents beryllation reaction.

xyz-Coordinates of Optimised Complexes **4** – **7**:

**4**

| | | | |
|---|---|---|---|
| Mn | 1.75519761970893 | 3.26514893290055 | 4.93485393508083 |
| O | 2.05390945592728 | 2.43868662935519 | 7.70961398601362 |
| O | 3.70338301963407 | 5.40903975338928 | 4.66105286587014 |
| C | 0.34203905183593 | 3.77976136352459 | 3.43339925241370 |
| H | 0.06579887696200 | 4.78970772532953 | 3.16925771999903 |
| C | 1.42538883849757 | 1.74975295727720 | 3.48239612673622 |
| H | 2.08213143437780 | 0.93166944789495 | 3.23083042133870 |
| C | -0.28536634782512 | 2.97100710623259 | 4.42160463792182 |
| H | -1.13416095024610 | 3.24914147331896 | 5.02769643474645 |
| C | 0.37941742641103 | 1.71639642796844 | 4.44427074102675 |
| H | 0.14218483360071 | 0.88608130827820 | 5.09312735648102 |
| C | 1.39389470217855 | 3.02409483330703 | 2.85189853684554 |
| H | 2.06429461997537 | 3.36407010397334 | 2.07625975633877 |
| C | 1.96197386785513 | 2.79341127600059 | 6.60004919360337 |
| C | 2.93388137644620 | 4.54151084980743 | 4.80651466730343 |
| C | 4.95704707811243 | 1.42418201406307 | 3.92827673772117 |
| H | 4.82696777477466 | 1.24491124470733 | 2.87095561996749 |
| C | 5.52333718317800 | 2.58332450326180 | 4.50960664035822 |
| H | 5.88587209059754 | 3.44886240506818 | 3.97532619284147 |
| C | 5.41455621640885 | 2.46480245342129 | 5.91717385452368 |
| H | 5.68887482256320 | 3.22018237442346 | 6.63882288983544 |
| C | 4.50441084344067 | 0.58363250801709 | 4.97837274041490 |
| H | 3.96910873349916 | -0.34720956134301 | 4.86072219690571 |
| C | 4.79197075775763 | 1.22547243611959 | 6.20630507713040 |
| H | 4.50125693191795 | 0.87753152912352 | 7.18627781576789 |
| Be | 3.69632548169361 | 2.32731962411169 | 5.06677058438914 |
| C | -0.42163494267521 | 5.33203361446807 | 7.31737460546670 |
| H | -1.16884674504744 | 4.63329586789920 | 7.66400759749731 |
| C | 1.55270655212535 | 6.47572683622962 | 7.10364774049949 |
| H | 2.57306965466638 | 6.79642484991638 | 7.25296786305070 |
| C | -0.48233281855084 | 6.09331113227502 | 6.12263563605461 |
| H | -1.28678805814045 | 6.07822700854780 | 5.40169694681089 |
| C | 0.73734359807742 | 6.80107800148453 | 5.99185064861161 |
| H | 1.03122747802349 | 7.41096808964094 | 5.15026742738489 |
| C | 0.83545650514617 | 5.57082755469027 | 7.92382665002023 |
| H | 1.21454289842476 | 5.07770674384800 | 8.80647991014614 |
| Be | 1.02748013866642 | 4.90425858146685 | 6.12833899288029 |

**5**

| | | | |
|---|---|---|---|
| Re | 11.42840735468673 | 2.67821234338344 | 3.10346290711728 |
| C | 13.38465331872764 | 6.15767244043813 | 2.49780048664371 |
| O | 13.43250163439005 | 2.13102655782803 | 5.35694481613085 |
| O | 13.14059143625884 | 2.11427814943349 | 0.62839727598464 |
| C | 13.77688586017862 | 5.88526535795854 | 3.83404765421523 |
| C | 9.58169497974132 | 3.14250816466485 | 4.41817348913288 |
| C | 9.75395747782368 | 4.00924129640869 | 2.28149750402040 |
| C | 9.90957045546842 | 4.31486711014315 | 3.67444559912498 |
| C | 14.92812109514153 | 4.46863449153315 | 2.45169513171508 |
| C | 10.16849212443556 | 5.66633968253609 | 4.26609600189532 |
| C | 14.09847301473780 | 5.28241415428453 | 1.64475602913793 |
| C | 14.73432236501371 | 4.84416018537560 | 3.80289319085157 |
| C | 9.87797158319604 | 4.97666628944743 | 1.14429846216796 |
| C | 12.51885799661117 | 2.31857778507512 | 1.59481010745128 |
| C | 12.69895000430982 | 2.33046077319451 | 4.47277019390200 |
| C | 9.21692625224036 | 2.11546415385850 | 3.49781569459591 |
| C | 9.54559398348930 | 3.03461406751486 | 5.91268101806286 |
| C | 8.95254091899104 | 1.94233609018147 | 0.90820193691975 |
| C | 8.60590042803905 | 0.79947006312050 | 3.86643289397654 |
| Be | 13.06419303634213 | 4.30365957211115 | 2.96909650194378 |
| C | 13.46348060252535 | -0.73574726862365 | 2.96299566539234 |
| C | 12.72242750921343 | -0.97784736970031 | 4.14499987986179 |
| C | 12.60567515278074 | -0.93591538423422 | 1.85533937054924 |
| C | 11.40166986612705 | -1.31664909458021 | 3.76928302808172 |
| C | 11.32846731215811 | -1.28853659929193 | 2.35170486020224 |
| C | 9.32735058977628 | 2.65039859095386 | 2.17486681077112 |
| Be | 11.90695324512640 | 0.41731240424659 | 3.06484436488255 |
| H | 13.37165079594838 | 6.34632358347572 | 4.72225158721182 |
| H | 10.70227917739000 | 6.31120414667777 | 3.56939639044076 |
| H | 10.76458230442962 | 5.59388323661157 | 5.17654713083814 |
| H | 9.22269684447546 | 6.15709180667927 | 4.52044038375602 |
| H | 13.96728388031642 | 5.18428707469984 | 0.57757583721563 |
| H | 15.16286809576538 | 4.35431536047039 | 4.66422120035662 |
| H | 10.23461480692852 | 4.48169918545271 | 0.24005190229339 |
| H | 10.58139622696100 | 5.77506187588481 | 1.38063647314711 |
| H | 8.90975855875023 | 5.43575690865509 | 0.91733235660650 |
| H | 9.76905266087987 | 2.01849729850402 | 6.24065225455140 |
| H | 8.55777146945639 | 3.30540696352587 | 6.30023447101793 |
| H | 10.28009430194771 | 3.69651306425711 | 6.37339645074015 |
| H | 9.54109491619616 | 2.30376763382782 | 0.06392338780629 |
| H | 7.89426192975233 | 2.09719895669718 | 0.67222663667646 |
| H | 9.12518820926937 | 0.86860454135527 | 0.99257870780370 |
| H | 7.52323523757805 | 0.91160645139286 | 3.99151594698371 |
| H | 9.01201335438845 | 0.42079503076912 | 4.80459208772646 |
| H | 8.77726524969417 | 0.04664061523650 | 3.09752307193946 |
| H | 14.48113771745050 | -0.37767893844689 | 2.91707328856768 |
| H | 10.57966126667226 | -1.50582830243249 | 4.44234475122431 |
| H | 10.44225365890347 | -1.45525290066972 | 1.75746950290806 |
| H | 12.85545936806608 | -0.74673555484594 | 0.82229970058760 |
| H | 13.07830695444007 | -0.83039161219570 | 5.15358603061155 |
| H | 12.64081048123056 | 6.87641239412632 | 2.18863800188846 |
| H | 15.53337493557858 | 3.64592717302992 | 2.10181157236960 |

**6**

| | | | |
|---|---|---|---|
| Mn | 1.84451179811417 | 3.38961322508076 | 4.89003239797588 |
| O | 1.33553714769804 | 1.85127916683445 | 7.31716093941918 |
| O | 3.92612358986397 | 5.36986070154350 | 5.31409397836198 |
| C | 1.00738460816949 | 4.29707953143860 | 3.17009256121180 |
| H | 1.03864490819217 | 5.36134608392345 | 2.99356840924473 |
| C | 1.57361903440813 | 2.07031792729069 | 3.23073843735250 |
| H | 2.11268519890394 | 1.14613557440070 | 3.10011009201914 |
| C | -0.01725264218262 | 3.60230775078846 | 3.86555405735513 |
| H | -0.89877044511033 | 4.04532594672062 | 4.30018258670194 |
| C | 0.33279417649816 | 2.22880345175528 | 3.89825882513275 |
| H | -0.23818776597893 | 1.44113076481509 | 4.36819415051613 |
| C | 1.99153793354412 | 3.34911062136924 | 2.77751072314281 |
| H | 2.90725782312643 | 3.56146297165423 | 2.24704865400425 |
| C | 1.56391619690660 | 2.48041323681586 | 6.38653612752745 |
| C | 3.10225077869017 | 4.56973164401824 | 5.18357343897505 |
| B | 3.62478342200080 | 2.42017324076220 | 5.17424727036179 |
| O | 4.32422506148425 | 1.82166014015174 | 4.14094201360521 |
| O | 4.30690382974653 | 2.28930799153251 | 6.36978695810743 |
| C | 5.64407701209917 | 1.47598271181837 | 4.63653071018443 |
| C | 5.39587500679453 | 1.35661086250713 | 6.16925618166942 |
| C | 6.10200910839151 | 0.19515572393505 | 3.96163630477886 |
| C | 6.57216871581488 | 2.63048872780638 | 4.27031378852165 |
| C | 4.88523598468066 | -0.01895951164284 | 6.58925771226537 |
| C | 6.56864544927763 | 1.77200618186524 | 7.04003444437865 |
| H | 6.24896963939773 | 0.37625902184464 | 2.89449522277543 |
| H | 7.05340062855129 | -0.13770007999526 | 4.38526237807383 |
| H | 5.36859596693576 | -0.60258312371853 | 4.07818452434088 |
| H | 7.60319488422422 | 2.41545250629322 | 4.55921341492964 |
| H | 6.54033706639724 | 2.77723132673987 | 3.18854533975016 |
| H | 6.25873429085005 | 3.55847749522399 | 4.75324778144289 |
| H | 5.67519375071837 | -0.77078011610294 | 6.52920630964488 |
| H | 4.53740473519985 | 0.03749483207663 | 7.62266708268679 |
| H | 4.04835220264479 | -0.33794773138708 | 5.96375495195473 |
| H | 6.30130998824723 | 1.65351826947051 | 8.09230038102565 |
| H | 7.43666330093977 | 1.13978574187487 | 6.83405573082778 |
| H | 6.84291900130945 | 2.81288994679154 | 6.87003729208528 |
| B | 1.13888673586890 | 4.84556472152325 | 6.14351438013853 |
| O | 1.37670661206922 | 4.95809210376909 | 7.50027862369735 |
| O | 0.34748963636879 | 5.88938122609034 | 5.69470018234910 |
| C | 0.89710466290410 | 6.25289553440218 | 7.93979140708941 |
| C | -0.17224944496550 | 6.58386930339321 | 6.85713495788128 |

**7**

| | | | |
|---|---|---|---|
| Re | 10.67716937324335 | 2.96845346419002 | 3.01780260632972 |
| O | 13.15772485859491 | 2.21814041271460 | 4.64883582657871 |
| O | 11.48250727073851 | 2.58394343703793 | 0.07564201298708 |
| C | 9.47471298093225 | 4.08718497662193 | 4.61535603202483 |
| C | 8.55862109642114 | 3.77915848696054 | 2.51592990446121 |
| C | 9.19399052428978 | 4.72870228741125 | 3.36757906169290 |
| C | 9.35188924169897 | 6.18639208435856 | 3.06239762575407 |
| C | 8.03316719915626 | 4.05607354362506 | 1.14040285750320 |
| C | 11.23728252919419 | 2.71591272400093 | 1.18716474628385 |
| C | 12.24226058139986 | 2.49172989072217 | 4.00085167178419 |
| C | 8.98668670595319 | 2.74142619324167 | 4.53701330997760 |
| C | 10.04367960141146 | 4.75135026965499 | 5.83198145498002 |
| C | 7.62747566963946 | 1.37017076948297 | 2.76876361341351 |
| C | 8.93260822547189 | 1.76022046640627 | 5.66746558090800 |
| C | 8.41247912584300 | 2.55693513390663 | 3.23629471727171 |
| H | 9.61275952408059 | 6.35119156091215 | 2.01589328221821 |
| H | 10.12848004288789 | 6.63787691309475 | 3.67444187155003 |
| H | 8.40541064446533 | 6.70223290278193 | 3.25671484066080 |
| H | 8.03397997637459 | 3.15524266685150 | 0.52572055953700 |
| H | 8.63657292259701 | 4.80801253631694 | 0.63062723244602 |
| H | 7.00463124181610 | 4.42786348810098 | 1.19122938206479 |
| H | 10.52012621182628 | 4.02406267071661 | 6.49048426381976 |
| H | 9.25219068713307 | 5.25078304674807 | 6.40060723095319 |
| H | 10.79400310470665 | 5.48994566802021 | 5.55305577826608 |
| H | 7.75588124952324 | 1.20557874033469 | 1.69789374368259 |
| H | 6.56160153277618 | 1.53645870247814 | 2.95835699475957 |
| H | 7.93811520899157 | 0.46404197461354 | 3.28221262513605 |
| H | 7.99557762895742 | 1.87334639324795 | 6.22280549143376 |
| H | 9.75591712398189 | 1.91805822561895 | 6.36548063275975 |
| H | 8.99844456305453 | 0.73842570304303 | 5.29754995112408 |
| H | 12.20784974339611 | 6.83659128532328 | 1.01011660851653 |
| H | 13.33989979661261 | 6.14864533235242 | -0.16210168075754 |
| H | 13.77063558030208 | 7.62743825631050 | 0.71737085192357 |
| C | 13.26523122354125 | 6.66225604991912 | 0.79815547278271 |
| H | 12.34903610405857 | -0.80532130840285 | 4.79406136299252 |
| H | 12.62225150160529 | 8.15570424488642 | 2.85411431275823 |
| H | 10.88237093494152 | -1.56187493735620 | 5.43488864329729 |
| C | 11.61989389630123 | -1.60189737170200 | 4.63011143942482 |
| H | 12.13290075785757 | -2.56481384640952 | 4.68252525213185 |
| C | 13.42877040454281 | 7.76592948476556 | 3.47477563565101 |
| H | 14.35803233099120 | 8.27316097833980 | 3.20062732426640 |

| | | | |
|---|---|---|---|
| C | 0.35629954055327 | 6.12513755123505 | 9.35307132293762 |
| C | 2.09032957429715 | 7.20332833246749 | 7.91211419086087 |
| C | -1.53893051169905 | 5.97653961932596 | 7.16226786859723 |
| C | -0.31271529515331 | 8.05962227217748 | 6.52873625329118 |
| H | 1.17599416192577 | 5.90061970141064 | 10.03888705926968 |
| H | -0.10582221179383 | 7.06472870186162 | 9.66805539510552 |
| H | -0.38245645546785 | 5.32738743149185 | 9.42793049461951 |
| H | 1.81896550123831 | 8.18788236648255 | 8.29934341952298 |
| H | 2.88202542336962 | 6.78982986914892 | 8.54013168049796 |
| H | 2.48255150941402 | 7.31867700848809 | 6.89993306330320 |
| H | -2.02467691569635 | 6.49016973979986 | 7.99464396879104 |
| H | -2.17326921365509 | 6.07609438893484 | 6.27869419272020 |
| H | -1.45273948083180 | 4.91569134299206 | 7.40831179444676 |
| H | -1.08270504652940 | 8.19381700335280 | 5.76561147613352 |
| H | -0.61347306256957 | 8.61799513545374 | 7.41935284132180 |
| H | 0.62173289380295 | 8.47573388990190 | 6.15296425507281 |

| | | | |
|---|---|---|---|
| C | 13.90026201772987 | 5.78234779250389 | 1.87288365634940 |
| H | 13.75807199457115 | -1.57965615349473 | 3.11413956250894 |
| H | 15.50282105533577 | 5.24141344162333 | 0.55079441523499 |
| O | 13.23050836037741 | 4.50688451044208 | 1.82245945717482 |
| B | 12.20204069761437 | 4.51381870368153 | 2.74759719881516 |
| H | 13.20672845271198 | 8.00646131264180 | 4.51705466242872 |
| C | 15.37716870716786 | 5.57769798989291 | 1.58199528788780 |
| H | 15.92743067456028 | 6.51479692657287 | 1.70614383043168 |
| C | 13.57771003312962 | 6.26228275854313 | 3.31797974170682 |
| O | 12.29936428290377 | 5.63347864259464 | 3.56402773190298 |
| B | 11.01954096544161 | 0.81877517987150 | 2.80649703180393 |
| C | 10.90161625850011 | -1.42151550700937 | 3.29540220788166 |
| C | 13.21250752782968 | -1.90763803916403 | 2.22982615809362 |
| O | 10.20342523035067 | -0.15755770384486 | 3.36174384465443 |
| O | 12.10042474748069 | 0.23072960333296 | 2.17055428105417 |
| H | 13.06446949312396 | -2.98995780233236 | 2.28591036369539 |
| C | 11.87859648042798 | -1.19107004280016 | 2.10540647136582 |
| H | 9.24843280845351 | -2.61795037556354 | 3.96775023755998 |
| H | 13.82586099486419 | -1.69165704415659 | 1.35272748508422 |
| C | 9.88653160880549 | -2.53202671638682 | 3.08523576575981 |
| H | 15.80995314962916 | 4.82458171878528 | 2.23966378773688 |
| H | 10.39060977062912 | -3.49087971626216 | 2.93603459605650 |
| C | 14.54935666120288 | 5.71297190390152 | 4.35910171111091 |
| H | 15.53114645885925 | 6.18524509532875 | 4.27886373238814 |
| H | 14.14874356755423 | 5.91678651187264 | 5.35452890194148 |
| H | 9.25219674478382 | -2.33245290811581 | 2.22202033724183 |
| H | 14.66737628197316 | 4.63269243070008 | 4.25675782383165 |
| C | 11.24397306015611 | -1.48151589664288 | 0.74719934093383 |
| H | 11.10241355824635 | -2.55318751780850 | 0.58913185484554 |
| H | 11.90208054308083 | -1.09690771969632 | -0.03413033426741 |
| H | 10.27768889119171 | -0.98117488025301 | 0.65163873143587 |

## xyz-Coordinates of Optimised Species from Mechanistic Studies:

**1**

| | | | |
|---|---|---|---|
| Mn | 2.51574510404997 | 0.79861277173511 | -0.32254457905561 |
| C | 2.89831247580110 | -0.90779144516986 | 0.05701916861339 |
| C | 2.89294118504640 | 1.32537240897170 | 1.34560134947354 |
| C | 0.77768290537827 | 0.58572798387303 | 0.04149908067250 |
| C | 4.24660809468933 | 1.41482061130674 | -1.42240414201204 |
| C | 3.46887989686366 | 2.55842636306150 | -1.08749337387084 |
| C | 3.43976930315069 | 0.57620440360970 | -2.24248449707738 |
| H | 5.26822896654899 | 1.22857309465500 | -1.12801524613467 |
| C | 2.19222319347516 | 2.42254557523841 | -1.68117192303300 |
| H | 3.79237213237023 | 3.38311471697601 | -0.46905120426635 |
| C | 2.17474493270863 | 1.18800517528463 | -2.40076627211626 |
| H | 3.73726071144238 | -0.37489350073496 | -2.65972390957417 |
| H | 1.37869633437931 | 3.12877816121479 | -1.60880850872193 |
| H | 1.34480458543043 | 0.79409784883798 | -2.96819810516167 |
| O | 3.15769834849716 | 1.68869357166898 | 2.39859456137814 |
| O | -0.34440109704611 | 0.46992632040733 | 0.23917676610226 |
| O | 3.16727186721433 | -2.00025453093613 | 0.26951097478412 |

**S1**

| | | | |
|---|---|---|---|
| Mn | 2.55188590048798 | 0.86119270527229 | -0.26415853577176 |
| C | 2.93919296846567 | -0.86898103761251 | 0.20001843368365 |
| C | 2.83703200593516 | 1.57866780476649 | 1.61868821710837 |
| C | 0.80949747737804 | 0.44899223105046 | 0.08315540241071 |
| C | 4.27113249682005 | 1.37289738391180 | -1.57538272459826 |
| C | 3.61315838988324 | 2.58025749785899 | -1.24654799340345 |
| C | 3.37776377270296 | 0.58293691970971 | -2.36785179330794 |
| H | 5.29258762161987 | 1.11494527517041 | -1.33423647675912 |
| C | 2.28651442415476 | 2.49354769859893 | -1.73197693969252 |
| H | 4.02208576004369 | 3.38593513614061 | -0.65489163707343 |
| C | 2.16210304781683 | 1.26892605600469 | -2.46465741387173 |
| H | 3.59476282226075 | -0.39170607165394 | -2.78148012836489 |
| H | 1.52074199119448 | 3.25015278547680 | -1.63796887269818 |
| H | 1.27186638820336 | 0.92142614649835 | -2.96907897651915 |
| O | 2.69398201539294 | 1.48787883986235 | 2.74713349487930 |
| O | -0.27670547401168 | 0.15429382324795 | 0.27841949562678 |
| O | 3.14123733165156 | -1.96140366430358 | 0.46155658835173 |

**ST1**

| | | | |
|---|---|---|---|
| Mn | 2.49809100126292 | 0.79906130464909 | -0.37361887382794 |
| C | 2.96058612327286 | -0.71570521036991 | 0.52545737025162 |
| C | 2.57466947864241 | 1.94982204768141 | 1.81674882321437 |
| C | 0.77191763313877 | 0.31631458410558 | -0.02575498395507 |
| C | 4.30405617445232 | 1.32813144283804 | -1.46965927502411 |
| C | 3.49206519322928 | 2.48328330461440 | -1.34896303442079 |
| C | 3.59748532613864 | 0.37344208192116 | -2.26639309767684 |
| H | 5.30194771519281 | 1.20411033534283 | -1.07444609527869 |
| C | 2.26323367483464 | 2.21945765241876 | -2.00487723046796 |
| H | 3.74318851348690 | 3.38413584798051 | -0.80817718056599 |
| C | 2.34836693210596 | 0.92016506032771 | -2.59674975276854 |
| H | 3.95355273196865 | -0.61025247567736 | -2.53666148402950 |
| H | 1.42669493098153 | 2.89788215569351 | -2.09104764787372 |
| H | 1.57014105618774 | 0.43253382013726 | -3.16602323113799 |
| O | 2.28181664429593 | 1.69178814069249 | 2.87860605126676 |
| O | -0.29405490463864 | -0.05154951477153 | 0.16602596700910 |
| O | 3.20269275550843 | -1.70061649126627 | 1.05566827411792 |

**$CpMn(CO)_2$**

| | | | |
|---|---|---|---|
| Mn | 1.42668646093752 | 3.49543414808956 | 5.06860610520085 |
| O | 2.60183473068719 | 1.98705160197826 | 7.29519496396068 |
| O | 3.75936902741698 | 5.22924583917425 | 4.66044597404212 |
| C | 0.61923963427428 | 3.72720532682315 | 3.09958076849714 |
| H | 0.59665018899331 | 4.64119547274524 | 2.52534829119608 |
| C | 1.29011581335114 | 1.69868541659492 | 3.96627445939527 |
| H | 1.87053855701068 | 0.81258302089701 | 4.17722777031586 |
| C | -0.39983300971372 | 3.25035626089269 | 3.95859622917177 |
| H | -1.31128177461507 | 3.77997276192011 | 4.20388224082421 |
| C | -0.00301270135527 | 2.00553222951891 | 4.50382684554387 |
| H | -0.57887012936980 | 1.38863695541002 | 5.17719929736796 |
| C | 1.67153385848001 | 2.75359629308725 | 3.10596146422363 |
| H | 2.59349126849767 | 2.81262694709267 | 2.54646011803111 |
| C | 2.13035038527971 | 2.62530658063512 | 6.46499546471432 |
| C | 2.83028769012535 | 4.58757114514075 | 4.87010000751499 |

**T2Be**

| | | | |
|---|---|---|---|
| Mn | 1.58832428059681 | 2.74023857732906 | 4.50453497417500 |
| O | 2.51794244662915 | 1.94795601704731 | 7.16185490124389 |
| O | 3.99683681050935 | 4.26869700677311 | 3.83994194916695 |
| C | 0.97235894232653 | 2.41599522827345 | 2.47256062397026 |
| H | 1.06550997064280 | 3.12987418474159 | 1.66786715665311 |
| C | 1.42822745993199 | 0.70834889818888 | 3.94930905330360 |
| H | 1.92845842474015 | -0.09720045513477 | 4.46663852115207 |
| C | -0.14996073658482 | 2.24827169988157 | 3.31669492105245 |
| H | -1.04308288078599 | 2.85606955265518 | 3.29664201777827 |
| C | 0.11479743516664 | 1.20252477743740 | 4.23293706146880 |
| H | -0.55836415110950 | 0.82835866516797 | 4.98941166288454 |
| C | 1.95491526491072 | 1.45242696409800 | 2.86946819746486 |
| H | 2.92742185643314 | 1.31497956293962 | 2.42004853729458 |
| C | 2.14920940327104 | 2.30802319221124 | 6.12994337247699 |
| C | 3.03677040602462 | 3.70384539148791 | 4.13584020935092 |
| Be | 0.71408777524559 | 6.16776003289654 | 4.95108715505087 |
| C | 1.62799010400954 | 6.93411263242785 | 3.45882100035168 |

**T2B**

| | | | |
|---|---|---|---|
| Mn | 1.41732782987695 | 2.74160432525115 | 4.19391913302918 |
| O | 2.61854489341677 | 1.44917096535767 | 6.53188412237967 |
| O | 3.99681985222231 | 3.22523332940003 | 2.88396779142710 |
| C | 0.39434848116336 | 2.60093857445352 | 2.31173894588794 |
| H | 0.65106073071181 | 3.14186139282973 | 1.41362883402654 |
| C | 0.30378945279174 | 0.96524530615322 | 3.92471779204081 |
| H | 0.48963048068238 | 0.05600599989416 | 4.47820977937188 |
| C | -0.52510208202724 | 3.00996518454291 | 3.30280064643066 |
| H | -1.05173658068520 | 3.95378518122833 | 3.32264919602718 |
| C | -0.59202278285129 | 2.00975515632166 | 4.30655331983781 |
| H | -1.22009739681365 | 2.03245177951471 | 5.18058880690049 |
| C | 0.91614187771985 | 1.32553631041629 | 2.70205966687270 |
| H | 1.63898014165743 | 0.73866151799453 | 2.15504041404532 |
| C | 2.15792755875599 | 2.02248352863950 | 5.64844798884464 |
| C | 2.99946801190026 | 3.08507544111936 | 3.43561249241282 |
| B | 0.51833596789757 | 4.56969924660007 | 6.36233696431167 |
| B | 0.99747126623884 | 5.48287745418491 | 4.97276941170713 |

| | | | |
|---|---|---|---|
| Be | -0.20466248060059 | 4.58379747077447 | 6.06588530665758 |
| C | 0.28737305713521 | 7.38544924162764 | 3.54301594303336 |
| C | 2.27160906664035 | 7.24041276706588 | 4.68232571674429 |
| H | 2.06164777294919 | 6.37939527137607 | 2.64033400227179 |
| C | -0.35152903149388 | 4.17392907202239 | 7.95116730579939 |
| C | 0.10233772140802 | 7.97243124358718 | 4.82008223307735 |
| H | -0.47783803750102 | 7.24276702219287 | 2.79440147073983 |
| C | 1.32930239795322 | 7.88236756408772 | 5.52420551655501 |
| H | 3.27831638948275 | 6.95878156415987 | 4.95257058207921 |
| C | -0.82218726833026 | 3.11606331486926 | 7.13647607694397 |
| C | -1.09585986922916 | 5.33314686830555 | 7.63245621927479 |
| H | 0.47861072514346 | 4.11741343381972 | 8.63971060192967 |
| H | -0.82814057573636 | 8.35396011431585 | 5.21395598429558 |
| H | 1.49647971767902 | 8.18385827738047 | 6.54769218384914 |
| C | -1.85879975327207 | 3.62442051425696 | 6.31313447673831 |
| H | -0.43068804322586 | 2.11243893931165 | 7.11661274457528 |
| C | -2.02838294597022 | 4.99319974777534 | 6.62256019724158 |
| H | -0.92886115777789 | 6.32271141665365 | 8.03152279522089 |
| H | -2.38349684854068 | 3.07549044231608 | 5.54534555425128 |
| H | -2.69497807867129 | 5.67846267567731 | 6.11997533388041 |

| | | | |
|---|---|---|---|
| O | -0.78262740038517 | 4.29922512631928 | 6.69694597895163 |
| O | 1.38173006993970 | 4.40954391727794 | 7.41607720720933 |
| O | 0.11838858622113 | 6.27983437608594 | 4.28919946622467 |
| O | 2.30284307105553 | 5.71792367273011 | 4.63417057501035 |
| C | -0.79336177584807 | 3.75985070307134 | 8.04949993545777 |
| C | 0.58269098570723 | 4.24017687999185 | 8.61804207320356 |
| C | 0.87339072548503 | 7.02034160771953 | 3.28703686561844 |
| C | 2.33495629634100 | 6.93629436183007 | 3.83401527390848 |
| C | -2.00821916309627 | 4.31251424971917 | 8.77608562461686 |
| C | -0.90207617232571 | 2.24306045887969 | 7.94011395881917 |
| C | 0.51372080935363 | 5.61229692324738 | 9.28226366338466 |
| C | 1.27770979750605 | 3.24713276117311 | 9.53307362041099 |
| C | 0.68654283102552 | 6.29145421203374 | 1.96212894947232 |
| C | 0.30777060966640 | 8.42700057112616 | 3.19649377231698 |
| C | 3.41028317034225 | 6.80298840096965 | 2.77079704796556 |
| C | 2.68333902505517 | 8.06474675069934 | 4.79957091026258 |
| H | -2.91675178935642 | 3.92736237771195 | 8.30822223011345 |
| H | -2.00057003220943 | 3.99436647083386 | 9.82186425133874 |
| H | -2.03784267692846 | 5.40105155133002 | 8.73990262024112 |
| H | -0.95081426706747 | 1.78743005286748 | 8.93108716816018 |
| H | -1.81985040280923 | 1.99024047746253 | 7.40523969231125 |
| H | -0.05544215213323 | 1.81655575332364 | 7.40152187716611 |
| H | -0.02827792206987 | 5.56664635774350 | 10.22911817945434 |
| H | 1.53073559601507 | 5.95589249146945 | 9.48119896428785 |
| H | 0.02524861850796 | 6.34201163366744 | 8.63274795866567 |
| H | 2.22463041078426 | 3.67093744643009 | 9.87401692173594 |
| H | 0.65828603595431 | 3.04369193380170 | 10.41061111082176 |
| H | 1.48677168791560 | 2.30838960789180 | 9.02173218431450 |
| H | -0.38180341666146 | 6.20670403750409 | 1.75245616266409 |
| H | 1.15749859141600 | 6.84135756104079 | 1.14471351046150 |
| H | 1.11302630084723 | 5.28937612929948 | 2.00287944600265 |
| H | 0.91176107645935 | 9.03376038778138 | 2.51683162568975 |
| H | -0.71018023833709 | 8.38411972703319 | 2.80336676781413 |
| H | 0.28047268494490 | 8.91294531636007 | 4.17136839794358 |
| H | 3.40635114658724 | 7.68300569485459 | 2.12225031641212 |
| H | 4.38868745842381 | 6.73505067208428 | 3.25059266991079 |
| H | 3.26761055769284 | 5.91400790802253 | 2.15888095733241 |
| H | 3.63771051011336 | 7.83581308621473 | 5.27749617121426 |
| H | 2.77824944550840 | 9.01631926934230 | 4.27263089495506 |
| H | 1.92530460549479 | 8.16850312155460 | 5.57900986386799 |

**I1Be**

| | | | |
|---|---|---|---|
| Mn | 1.41955556021897 | 3.22308337774839 | 4.66496489612544 |
| O | 2.20504023558709 | 2.13232944538650 | 7.24206546786388 |
| O | 4.08540003743710 | 4.37067519420973 | 4.52630695592944 |
| C | 0.81858524564679 | 3.23185718032776 | 2.61518417488944 |
| H | 0.91537379607221 | 4.06200943263030 | 1.93305601440337 |
| C | 1.26616539693429 | 1.30094244094085 | 3.77930315653681 |
| H | 1.76558368997922 | 0.42176789312616 | 4.15838981440503 |
| C | -0.30642305187462 | 2.94641885893935 | 3.43520875427509 |
| H | -1.21857107869355 | 3.52179414250711 | 3.48183056408603 |
| C | -0.03206719787962 | 1.75165318804490 | 4.15269052840954 |
| H | -0.69017359086702 | 1.26459719895624 | 4.85567243433540 |
| C | 1.78791229814647 | 2.21242073563776 | 2.83266193153133 |
| H | 2.75853549556164 | 2.15249197229672 | 2.36249981318987 |
| C | 1.85718610595202 | 2.60886514933106 | 6.24237663792355 |
| C | 2.99635590217313 | 3.97659022654833 | 4.60348399682957 |
| Be | 1.17573906874493 | 5.44115630247961 | 4.60948100767043 |
| C | 1.83229411116689 | 6.65312693442383 | 3.22217753301803 |
| Be | -0.01917122055009 | 4.01386667535431 | 6.17118789913287 |
| C | 0.41834708737213 | 6.66761765009649 | 3.29636573935662 |
| C | 2.34053499533898 | 7.00968217055356 | 4.49148310364234 |
| H | 2.42105835009285 | 6.33757450943712 | 2.37338369644125 |
| C | -0.29371241487957 | 4.19822888632516 | 8.08896702414095 |
| C | 0.05431114213871 | 7.04039214349234 | 4.61138257495628 |
| H | -0.26339694356247 | 6.38583301080675 | 2.50702690340339 |
| C | 1.24351497855981 | 7.24236034411375 | 5.35375876646087 |
| H | 3.38187350823731 | 7.01576993501242 | 4.77542819888996 |
| C | -0.90223653637504 | 3.01465179878894 | 7.60839325054898 |
| C | -0.84784687284809 | 5.29211134954152 | 7.38184503739199 |
| H | 0.50256807315522 | 4.24905003442809 | 8.81684043007931 |
| H | -0.95179278400187 | 7.10273751393705 | 4.99455558442397 |
| H | 1.30758716473289 | 7.48114929114456 | 6.40483265357086 |
| C | -1.82195663636176 | 3.37470066477007 | 6.59818249145232 |
| H | -0.63700405952225 | 2.00852681292989 | 7.89688736204882 |
| C | -1.79018733083842 | 4.78218255780187 | 6.45599109272605 |
| H | -0.55950489544917 | 6.32597718465314 | 7.49109274623002 |
| H | -2.40090006812962 | 2.69250234713847 | 5.99313497468856 |
| H | -2.34688199141660 | 5.35408433613904 | 5.72893834899186 |

**I1B**

| | | | |
|---|---|---|---|
| Mn | 1.12047796916943 | 2.73099262876309 | 4.28059661947996 |
| O | 2.54870873501713 | 1.17186885662286 | 6.29304783861633 |
| O | 3.67282568321330 | 3.28199187638366 | 2.97696737930781 |
| C | -0.08521117517783 | 2.84070493648476 | 2.52160905253027 |
| H | 0.01407949123553 | 3.61246536983004 | 1.77471348529750 |
| C | 0.19961756195171 | 0.88768973719889 | 3.69583126795463 |
| H | 0.55761318050750 | -0.08508268395699 | 4.00037686753648 |
| C | -0.91797012677757 | 2.89209355179205 | 3.66608224940349 |
| H | -1.53707735954097 | 3.72453419409083 | 3.95841130652561 |
| C | -0.74245860017983 | 1.69214515608818 | 4.39917519152271 |
| H | -1.23124609164430 | 1.43798648172011 | 5.32624069567822 |
| C | 0.60460419734124 | 1.59383968195589 | 2.54165844850965 |
| H | 1.32464068880444 | 1.25355279880444 | 1.81171387938197 |
| C | 1.99818272697028 | 1.83129255260540 | 5.53368131669162 |
| C | 2.67868982364020 | 3.10859257940679 | 3.52217851067678 |
| B | 0.70105322146436 | 3.76393747357923 | 6.04761508226343 |
| B | 1.27319902064220 | 4.81087263639689 | 4.38621733115596 |
| O | -0.61581060152393 | 3.92293570793710 | 6.45954548319598 |
| O | 1.56358250748767 | 3.95907444248682 | 7.11355369825090 |
| O | 0.32416229903466 | 5.58887197824319 | 3.73470637390945 |
| O | 2.40589644258943 | 5.56145206877386 | 4.65464896517224 |
| C | -0.63617203118043 | 3.83238504312764 | 7.90410054905990 |
| C | 0.79830879664432 | 4.31008572020908 | 8.28901558481719 |
| C | 0.99816420485209 | 6.76223775827221 | 3.22190462072748 |
| C | 2.18509522239813 | 6.92354132105204 | 4.21955434463094 |
| C | -1.76556056110282 | 4.69606818378118 | 8.43847556463800 |
| C | -0.88919799445840 | 2.36679156760106 | 8.25234020951490 |
| C | 0.89616422413060 | 5.81920379857458 | 8.47440251737586 |
| C | 1.40578556693786 | 3.60064800088693 | 9.48879477854156 |
| C | 1.45370410464668 | 6.43037328553395 | 1.80221853554857 |
| C | 0.01929660560871 | 7.92347967905470 | 3.19469410996242 |
| C | 3.47095321302399 | 7.44882819609417 | 3.60239386337571 |
| C | 1.81897626811289 | 7.74873580688048 | 5.44698538981123 |
| H | -2.72460880015727 | 4.27999979363900 | 8.12181157490337 |
| H | -1.74514146082687 | 4.71332872974280 | 9.53144931258563 |
| H | -1.69625839789765 | 5.71919119412230 | 8.07004389428453 |
| H | -0.97025183733771 | 2.22453049758574 | 9.33193408277513 |
| H | -1.82896930996010 | 2.05329147529827 | 7.79244875169403 |
| H | -0.08937761277043 | 1.72514300988899 | 7.87580834215169 |
| H | 0.42357143258743 | 6.12941243898837 | 9.40913809552602 |
| H | 1.94966156787939 | 6.10381740298922 | 8.50543072594936 |
| H | 0.42058406768504 | 6.34945904468543 | 7.65045383052481 |
| H | 2.40009543453819 | 4.00775902723585 | 9.68502215792578 |
| H | 0.78957176541748 | 3.75962659897984 | 10.37792150211371 |
| H | 1.50321863595192 | 2.52943533426372 | 9.31378091722930 |
| H | 0.58133302553241 | 6.14266265311423 | 1.21134334513327 |
| H | 1.92234726735669 | 7.29376770299566 | 1.32526659253698 |
| H | 2.16471967904560 | 5.60143680269769 | 1.79501298823930 |
| H | 0.52969573511091 | 8.84300917111664 | 2.89580376542148 |
| H | -0.76918581128640 | 7.71953419482926 | 2.46669604543688 |
| H | -0.44460599472086 | 8.08013330137595 | 4.16807955136746 |
| H | 3.30711225419642 | 8.43840721514901 | 3.16717704258798 |
| H | 4.23563141384245 | 7.53852353687466 | 4.37709724080648 |
| H | 3.84542535885278 | 6.78037087701030 | 2.82735836193289 |
| H | 2.59504397589972 | 7.62225391635888 | 6.20415558366753 |
| H | 1.74472415615815 | 8.80957593944809 | 5.19726173467670 |
| H | 0.86825136386107 | 7.42279998533376 | 5.86819996946351 |

**T3Be**

| | | | |
|---|---|---|---|
| Mn | -0.17924776770909 | 0.38936923526105 | -1.15780323786842 |

**T3B**

| | | | |
|---|---|---|---|
| Mn | 0.08817280277060 | -1.78752142336113 | -1.11390884521854 |

| | | | |
|---|---|---|---|
| C | -1.96926013389496 | 0.21584495298506 | -2.27182022677592 |
| H | -2.55831407046017 | 1.05352023789532 | -2.61483192603191 |
| C | -0.90315809227897 | -0.40586741260922 | -2.99448985075663 |
| H | -0.54899059752584 | -0.12020146271453 | -3.97321163828183 |
| C | -0.40064695941286 | -1.45556967364465 | -2.20007745432884 |
| H | 0.37849966124739 | -2.14361216511776 | -2.48913819801511 |
| C | -1.15503039790277 | -1.48854678758443 | -0.97536823038622 |
| H | -1.07836303158393 | -2.22366420140436 | -0.19104604837731 |
| C | -2.12630810150659 | -0.45256496604782 | -1.03155491762132 |
| H | -2.89129841343603 | -0.25863934512166 | -0.29663066059910 |
| O | 2.20605156688386 | 1.60627515522218 | -0.02150839652545 |
| O | -0.12340655459518 | 3.12548960341401 | -2.15704377804489 |
| C | 1.29344319976537 | 0.98393458140716 | -0.44488274244372 |
| C | -0.12737880938784 | 2.04209372416987 | -1.75167574014694 |
| Be | -0.81157638728921 | 1.26273545906927 | 0.70852167076473 |
| Be | 1.33138698200240 | -1.01228925434103 | -0.20537328831349 |
| C | -0.20656554977587 | 2.42797821378895 | 2.16053236994861 |
| H | 0.77846553182261 | 2.86739772017036 | 2.21111945005394 |
| C | -1.32630728907682 | 2.97668475678973 | 1.48985539878869 |
| H | -1.34564884359356 | 3.91348210206960 | 0.95223155701827 |
| C | -2.38446592556214 | 2.03634548920953 | 1.56045592961669 |
| H | -3.35323534249113 | 2.13663916388762 | 1.09291991704472 |
| C | -1.91623684510438 | 0.90374862381070 | 2.27287630108206 |
| H | -2.46767748131804 | -0.00886733916062 | 2.44755927101514 |
| C | -0.56924862537006 | 1.14711114027888 | 2.64297929773871 |
| H | 0.08608026033647 | 0.44970169419665 | 3.14410196342810 |
| C | 2.44230502886526 | -1.26912498450621 | 1.34677797608369 |
| H | 2.56685999740632 | -0.53838550576953 | 2.13181865029111 |
| C | 3.20065183474922 | -1.33440548108235 | 0.15343880951173 |
| H | 3.99451826111606 | -0.65829233481818 | -0.12505449053742 |
| C | 2.64548419786152 | -2.34680519522538 | -0.66439851709415 |
| H | 2.96078748914969 | -2.59579991740491 | -1.66706735172612 |
| C | 1.54650849673697 | -2.91385289473271 | 0.02790645961274 |
| H | 0.88082765168053 | -3.67256173904429 | -0.35632870860940 |
| C | 1.41871462899052 | -2.24455022893766 | 1.27045842149320 |
| H | 0.63451426664322 | -2.40001341417654 | 1.99701891141604 |

| | | | |
|---|---|---|---|
| C | -1.58022461116767 | -1.03757462321518 | -2.19632103070051 |
| H | -1.72281539572440 | -0.00046366451947 | -2.45165399682817 |
| C | -2.01135996215830 | -1.66243812936771 | -0.99304164547158 |
| H | -2.65749713844238 | -1.20663602307496 | -0.25910433659293 |
| C | -1.65301387448013 | -3.06031872626470 | -1.06060424840751 |
| H | -1.84187740708132 | -3.79572783634187 | -0.29511826566936 |
| C | -0.98231798163279 | -3.26478542030944 | -2.26738277898037 |
| H | -0.55466446721230 | -4.19766295204205 | -2.60490605106942 |
| C | -0.91853545826127 | -2.01136479840335 | -2.97457115768678 |
| H | -0.46546162869994 | -1.85208109255771 | -3.94134224902761 |
| C | 1.53461474526731 | -2.50878668802257 | -1.87778458799641 |
| O | 2.44327516652626 | -2.98898716608673 | -2.38057546094222 |
| C | 1.24715867223351 | -1.59076108014115 | 0.16927738306074 |
| O | 2.12498759171688 | -1.44014702854319 | 0.92218063379256 |
| B | 0.85254488171349 | 0.05439694228721 | -1.44864949342924 |
| O | 0.14420766284530 | 1.24210851500068 | -1.32280614151565 |
| C | 1.08514617546165 | 2.33774910497967 | -1.40578365884811 |
| C | 2.23393043779792 | 1.71355273327382 | -2.25059264842796 |
| O | 2.14448897711697 | 0.31615188209149 | -1.89004576952675 |
| C | 0.39980710520446 | 3.53416299269886 | -2.04294668582814 |
| H | -0.38630477852008 | 3.90154762695624 | -1.37920632736884 |
| H | -0.05206696466685 | 3.27365644168551 | -3.00012650293317 |
| H | 1.11765837283946 | 4.34349805459796 | -2.20247113691256 |
| C | 1.51016698038359 | 2.67041736830262 | 0.02263747851507 |
| H | 0.61740707114807 | 2.89682928701226 | 0.60924456850537 |
| H | 2.01711674114855 | 1.82301735654969 | 0.48944710675481 |
| H | 2.17425060832841 | 3.53740623124613 | 0.04840065836342 |
| C | 1.98562024853536 | 1.80571496954378 | -3.75423437990591 |
| H | 0.98289162314281 | 1.45413910107085 | -4.00804758780198 |
| H | 2.71245156905817 | 1.17402710574109 | -4.26933458084345 |
| H | 2.10031090743716 | 2.83036715317036 | -4.11489164914391 |
| C | 3.62431283492987 | 2.22514307366963 | -1.91523147536581 |
| H | 4.35938520307423 | 1.73552207367073 | -2.55804440713808 |
| H | 3.68480053475116 | 3.30358624220026 | -2.08649289782220 |
| H | 3.88481524905115 | 2.01889328729007 | -0.87729498814822 |
| B | -0.57625430873842 | -1.35818934239591 | 0.85531340295161 |
| O | -1.00163796924214 | -2.33051875930626 | 1.73066876919358 |
| C | -1.17864593303361 | -1.69788727651684 | 3.02918251533932 |
| C | -1.39158449953663 | -0.19786953537249 | 2.64378206252761 |
| O | -0.68622367664988 | -0.09710940407022 | 1.37845746841276 |
| C | 0.10554414923453 | -1.93951904078024 | 3.81538181221883 |
| H | 0.27634452202643 | -3.01570735375889 | 3.88474742760355 |
| H | 0.02713935150484 | -1.53568666459292 | 4.82693435713756 |
| H | 0.96686703061354 | -1.48777019717102 | 3.32107231381710 |
| C | -2.35609599149466 | -2.34865305832009 | 3.73319716939104 |
| H | -2.11419607046053 | -3.38915521329464 | 3.95989270114951 |
| H | -2.56452459019236 | -1.83390367860872 | 4.67480510055579 |
| H | -3.25503571690447 | -2.33023775012166 | 3.11725434104093 |
| C | -0.78151055864876 | 0.80122873587820 | 3.61181287985227 |
| H | 0.29777087414252 | 0.67319127172322 | 3.68880081471646 |
| H | -1.22344141132679 | 0.68660373217768 | 4.60505836845123 |
| H | -0.98435107682337 | 1.81567711176723 | 3.26158662251778 |
| C | -2.85076222801196 | 0.15870458098434 | 2.37877751100159 |
| H | -2.89122978866616 | 1.14252121715530 | 1.90716920899263 |
| H | -3.41841638569009 | 0.19690508866765 | 3.31086396171418 |
| H | -3.33299752865005 | -0.56246167035637 | 1.71662090478544 |

## I2Be (4)

| | | | |
|---|---|---|---|
| Mn | 1.75519761970893 | 3.26514893290055 | 4.93485393508083 |
| O | 2.05390945592728 | 2.43868662935519 | 7.70961398601362 |
| O | 3.70338301963407 | 5.40903975338928 | 4.66105286587014 |
| C | 0.34203905183593 | 3.77976136352459 | 3.43339925241370 |
| H | 0.06579887696200 | 4.78970772532953 | 3.16925771999903 |
| C | 1.42538883849757 | 1.74975295727720 | 3.48239612673622 |
| H | 2.08213143437780 | 0.93166944789495 | 3.23083042133870 |
| C | -0.28536634782512 | 2.97100710623259 | 4.42160463792182 |
| H | -1.13416095024610 | 3.24914147331896 | 5.02769643474645 |
| C | 0.37941742641103 | 1.71639642796844 | 4.44427074102675 |
| H | 0.14218483360071 | 0.88608130827820 | 5.09312735648102 |
| C | 1.39389470217855 | 3.02409483330703 | 2.85189853684554 |
| H | 2.06429461997537 | 3.36407010397334 | 2.07625975633877 |
| C | 1.96197386785513 | 2.79341127600059 | 6.60004919360337 |
| C | 2.93388137644620 | 4.54151084980743 | 4.80651466730343 |
| C | 4.95704707811243 | 1.42418201406307 | 3.92827673772117 |
| H | 4.82696777477466 | 1.24491124470733 | 2.87095561996749 |
| C | 5.52333718317800 | 2.58332450326180 | 4.50960664035822 |
| H | 5.88587209059754 | 3.44886240506818 | 3.97532619284147 |
| C | 5.41455621640885 | 2.46480245342129 | 5.91717385452368 |
| H | 5.68887482256320 | 3.22018237442346 | 6.63882288983544 |
| C | 4.50441084344067 | 0.58363250801709 | 4.97837274041490 |
| H | 3.96910873349916 | -0.34720956134301 | 4.86072219690571 |
| C | 4.79197075775763 | 1.22547243611959 | 6.20630507713040 |
| H | 4.50125693191795 | 0.87753152912352 | 7.18627781576789 |
| Be | 3.69632548169361 | 2.32731962411169 | 5.06677058438914 |
| C | -0.42163494267521 | 5.33203361446807 | 7.31737460546670 |
| H | -1.16884674504744 | 4.63329586789920 | 7.66400759749731 |
| C | 1.55270655212535 | 6.47572683622962 | 7.10364774049949 |
| H | 2.57306965466638 | 6.79642484991638 | 7.25296786305070 |
| C | -0.48233281855084 | 6.09331113227502 | 6.12263563605461 |
| H | -1.28676805814045 | 6.07822700854780 | 5.40169694681089 |
| C | 0.73734359807742 | 6.80107800148453 | 5.99185064861161 |
| H | 1.03122747802349 | 7.41096808964094 | 5.15026742738489 |
| C | 0.83545650514617 | 5.57082755469027 | 7.92382665002023 |
| H | 1.21454289842476 | 5.07770674384800 | 8.80647991014614 |
| Be | 1.02748013866642 | 4.90425858146685 | 6.12833899288029 |

## I2B (6)

| | | | |
|---|---|---|---|
| Mn | 1.84451179811417 | 3.38961322508076 | 4.89003239797588 |
| O | 1.33553714769804 | 1.85127916683445 | 7.31716093941918 |
| O | 3.92612358986597 | 5.36986070154350 | 5.31409397836198 |
| C | 1.00738460816949 | 4.29707953143860 | 3.17009256121180 |
| H | 1.03864490819217 | 5.36134608392345 | 2.99356840924473 |
| C | 1.57361903440813 | 2.07031792729069 | 3.23073843735250 |
| H | 2.11268519890394 | 1.14613557440070 | 3.10011009201914 |
| C | -0.01725264218262 | 3.60230775078846 | 3.86555405735513 |
| H | -0.89877044511033 | 4.04532594672062 | 4.30018258670194 |
| C | 0.33279417649816 | 2.22880345175528 | 3.89825882513275 |
| H | -0.23818776597893 | 1.44113076481509 | 4.36819415051613 |
| C | 1.99153793354412 | 3.34911062136924 | 2.77751072314281 |
| H | 2.90725782312643 | 3.56146297165423 | 2.24704865400425 |
| C | 1.56391619690660 | 2.48041323681586 | 6.38653612752745 |
| C | 3.10225077869077 | 4.56973164401824 | 5.18357343897505 |
| B | 3.62478342200080 | 2.42017324076220 | 5.17424727036179 |
| O | 4.32422506148425 | 1.82166014015174 | 4.14094201360521 |
| O | 4.30690382974653 | 2.28930799153251 | 6.36978695810743 |
| C | 5.64407701209917 | 1.47598271181837 | 4.63653071018443 |
| C | 5.39587500679453 | 1.35661086250713 | 6.16925618166942 |
| C | 6.10200910839151 | 0.19515572393505 | 3.96163630477886 |
| C | 6.57216871581488 | 2.63048872780638 | 4.27031378852165 |
| C | 4.88523598468066 | -0.01895951164284 | 6.58925771226537 |
| C | 6.56864544927763 | 1.77200618186524 | 7.04003444437865 |
| H | 6.24896963939773 | 0.37625902184464 | 2.89449522277543 |
| H | 7.05340062855129 | -0.13770007999526 | 4.38526237807383 |
| H | 5.36859596693576 | -0.60258312371853 | 4.07818452434088 |
| H | 7.60319488422422 | 2.41545250629322 | 4.55921341492964 |
| H | 6.54033706639724 | 2.77723132673987 | 3.18854533975016 |
| H | 6.25873429085005 | 3.55847749522399 | 4.75324778144289 |
| H | 5.67519375071837 | -0.77078011610294 | 6.52920630964488 |
| H | 4.53740473519985 | 0.03749483207663 | 7.62266708268679 |
| H | 4.04835220264479 | -0.33794773138708 | 5.96375495195473 |
| H | 6.30130998824723 | 1.65351826947051 | 8.09230038102565 |
| H | 7.43666330093977 | 1.13978574187487 | 6.83405573082778 |
| H | 6.84291900130945 | 2.81288994679154 | 6.87003729208528 |
| B | 1.13888673586890 | 4.84556472152325 | 6.14351438013853 |
| O | 1.37670661206922 | 4.95809210376909 | 7.50027862369735 |
| O | 0.34747893636879 | 5.88938122609034 | 5.69470018234910 |
| C | 0.89710466290410 | 6.25289553440218 | 7.93979140708941 |
| C | -0.17224944496550 | 6.58386930339321 | 6.85713495788128 |
| C | 0.35629954055327 | 6.12513755123505 | 9.35307132293762 |
| C | 2.09032957429715 | 7.20332833246749 | 7.91211419086087 |

| | | | |
|---|---|---|---|
| C | -1.53893051169905 | 5.97653961932596 | 7.16226786859723 |
| C | -0.31271529515331 | 8.05962227217748 | 6.52873625329118 |
| H | 1.17599416192577 | 5.90061970141064 | 10.03888705926968 |
| H | -0.10582221179383 | 7.06472870186162 | 9.66805539510552 |
| H | -0.38245645546785 | 5.32738743149185 | 9.42793049461951 |
| H | 1.81896550123831 | 8.18788236648255 | 8.29934341952298 |
| H | 2.88202542336962 | 6.78982986914892 | 8.54013168049796 |
| H | 2.48255150941402 | 7.31867700848809 | 6.89993306330320 |
| H | -2.02467691569635 | 6.49016973979986 | 7.99464396879104 |
| H | -2.17326921365509 | 6.07609438893484 | 6.27869419272020 |
| H | -1.45273948083180 | 4.91569134299206 | 7.40831179444676 |
| H | -1.08270504652940 | 8.19381700335280 | 5.76561147613352 |
| H | -0.61347306256957 | 8.61799513545374 | 7.41935284132180 |
| H | 0.62173289380295 | 8.47573388990190 | 6.15296425507281 |

**S1I2Be**

| | | | |
|---|---|---|---|
| Mn | 1.41231401092059 | 3.13286804512969 | 4.83364208604561 |
| O | 1.36490885207296 | 1.85452040921448 | 7.45548784013641 |
| O | 3.25246890736206 | 5.48495938043244 | 4.55246690006519 |
| C | 0.72932186833518 | 3.54499749670301 | 2.78583247690726 |
| H | 0.90267295768965 | 4.47988576997818 | 2.27295114129330 |
| C | 1.00059135677549 | 1.40300255278434 | 3.55717759690670 |
| H | 1.40246896611514 | 0.41906171059699 | 3.75209333388783 |
| C | -0.42770795695243 | 3.20521033751230 | 3.53895676592745 |
| H | -1.26091122714564 | 3.86043819953523 | 3.75073567786559 |
| C | -0.25376951759272 | 1.90134873625458 | 4.04189362937711 |
| H | -0.94383754398855 | 1.36232202615952 | 4.67456541562294 |
| C | 1.58343547356350 | 2.40443703500326 | 2.74918264600596 |
| H | 2.52920812699659 | 2.34464876997760 | 2.23122721934661 |
| C | 1.40695897587448 | 2.42232580765599 | 6.44095084964390 |
| C | 2.74089156890380 | 4.41871476227290 | 4.78377125282033 |
| C | 4.77677966799465 | 1.46978180852602 | 4.20202415830320 |
| H | 4.46714046770008 | 0.91140639869134 | 3.33120400536108 |
| C | 5.53018399694155 | 2.67215650137241 | 4.19738463778298 |
| H | 5.88331952538431 | 3.19688899361775 | 3.32202265963249 |
| C | 5.63788074098895 | 3.12481008628803 | 5.53441770963990 |
| H | 6.08257334834668 | 4.05672436830789 | 5.85086826095124 |
| C | 4.42434098800107 | 1.17815914227441 | 5.54426782842675 |
| H | 3.78927811576185 | 0.36919600767414 | 5.87271534502418 |
| C | 4.95158954722718 | 2.20457038698901 | 6.36694374931586 |
| H | 4.79096242796442 | 2.30642427776337 | 7.43017272034286 |
| Be | 3.81783275051714 | 2.90028872251526 | 5.00289593262349 |
| C | -0.10478097283604 | 5.05788940323870 | 7.71898870602871 |
| H | -0.82887378266071 | 4.33312668464590 | 8.06051268860689 |
| C | 1.87574751030889 | 6.17087924611300 | 7.40460384573533 |
| H | 2.91712699804734 | 6.44835275268958 | 7.46961849766456 |
| C | -0.26866884048465 | 5.94975253688030 | 6.62823504034775 |
| H | -1.14797562297137 | 6.03649630971078 | 6.00728547636589 |
| C | 0.95337575271470 | 6.64412053202155 | 6.44100150838876 |
| H | 1.17890138562983 | 7.33010198206345 | 5.63866542242457 |
| C | 1.22111825217469 | 5.18955571169271 | 8.19488868248748 |
| H | 1.67676779092331 | 4.59029598673175 | 8.96930429052394 |
| Be | 1.19229513339422 | 4.75662111898915 | 6.33554400216542 |

**S1I2B**

| | | | |
|---|---|---|---|
| Mn | 1.83916491060202 | 3.11752156331530 | 5.59457612830540 |
| O | 2.29766938496549 | 2.42806435234364 | 8.59477891693255 |
| O | 3.91912107773514 | 5.11343073037790 | 5.93998255434241 |
| C | 0.55090737037593 | 3.56405878743331 | 3.74982695071561 |
| H | 0.43368296878824 | 4.56929103630758 | 3.37479063270194 |
| C | 1.38865805604629 | 1.48222419718771 | 4.20084582908118 |
| H | 1.97132419736129 | 0.57674118705509 | 4.11317162415876 |
| C | -0.21525159800277 | 2.98116093865693 | 4.82013171869429 |
| H | -1.07254961435716 | 3.43414241452582 | 5.29660757982666 |
| C | 0.24883688625629 | 1.66146905919991 | 5.01917745128913 |
| H | -0.12903364609793 | 0.95679295808739 | 5.74522763247752 |
| C | 1.52622250053925 | 2.65083781082225 | 3.37024448873164 |
| H | 2.30897061428716 | 2.81302441097175 | 2.64455741631238 |
| C | 1.89507328910250 | 2.43011636369945 | 7.53246254180095 |
| C | 3.08138179485256 | 4.31140106405748 | 5.79883388012297 |
| B | 3.77629942401641 | 2.39387452147193 | 5.35161313491637 |
| O | 4.53207614457679 | 2.44634714957793 | 4.20547275482922 |
| O | 4.36521061314255 | 1.62930063169087 | 6.33089046661121 |
| C | 5.83165059962604 | 1.87188771496996 | 4.51984355686779 |
| C | 5.49471822073943 | 0.94154865488883 | 5.72473608433391 |
| C | 6.36041171341102 | 1.15641996477177 | 3.28983443262716 |
| C | 6.74679528336089 | 3.03114604724273 | 4.89952591079832 |
| C | 5.00303596566830 | -0.43783776663081 | 5.29888398727568 |
| C | 6.59606457566531 | 0.81012964518138 | 6.76138937585447 |
| H | 6.56731488230543 | 1.88611597301530 | 2.50412762813423 |
| H | 7.29197633367088 | 0.63540386832904 | 3.52630754994959 |
| H | 5.64090034594697 | 0.43277336186123 | 2.90720179308235 |
| H | 7.76329999880491 | 2.68192372103291 | 5.09258548339852 |
| H | 6.77543999370770 | 3.74203945755646 | 4.07126227075606 |
| H | 6.37868526943259 | 3.55354786332877 | 5.78451277918567 |
| H | 5.82220004324053 | -1.04088005119244 | 4.90177683574925 |
| H | 4.58825236911150 | -0.94796729093953 | 6.17056242283931 |
| H | 4.22378902270249 | -0.36503547742229 | 4.53759818004855 |
| H | 6.26567689928812 | 0.14343283494721 | 7.56072033739807 |
| H | 7.49336421382079 | 0.38164008164350 | 6.30684702505815 |
| H | 6.84993361961713 | 1.77475936501675 | 7.19986125850054 |
| B | 1.18883270689230 | 4.94907731754421 | 6.36346495751206 |
| O | 0.98225427025211 | 5.14440798005645 | 7.70851305525255 |
| O | 0.76205630820626 | 6.01695566564103 | 5.61105502654040 |
| C | 0.59914272207878 | 6.53681009240171 | 7.89031086157205 |
| C | 0.00784026847882 | 6.89284077695871 | 6.49344347862188 |
| C | -0.38167941278010 | 6.63073237848237 | 9.04502548642739 |
| C | 1.87687990478487 | 7.30482547557762 | 8.21161526334222 |
| C | -1.46153598957746 | 6.50778157585381 | 6.35352325068717 |
| C | 0.22143829125479 | 8.33023093440878 | 6.05552814156533 |
| H | 0.12399730509091 | 6.36223541858987 | 9.97491617189647 |
| H | -0.75382145349272 | 7.65427685909194 | 9.14120819949855 |
| H | -1.22968641662050 | 5.96003697160263 | 8.90800920385003 |
| H | 1.66119918952380 | 8.35524786389092 | 8.41856316272723 |
| H | 2.33736445093266 | 6.86420820497357 | 9.09804662766726 |
| H | 2.59202849372185 | 7.24719206198821 | 7.38856027044269 |
| H | -2.10049363907569 | 7.17838464214947 | 6.93194849718915 |
| H | -1.74621574777510 | 6.57989622632304 | 5.30177084258353 |
| H | -1.63641432975721 | 5.48386816429048 | 6.69035657166972 |
| H | -0.23066774732119 | 8.48301243249847 | 5.07311104700178 |
| H | -0.25522767873620 | 9.01519769375411 | 6.76187517624528 |
| H | 1.28153174960777 | 8.57343411953762 | 5.98948609389669 |

**ST4Be**

| | | | |
|---|---|---|---|
| Mn | 1.75379579468904 | 3.09479721917969 | 5.24000071590632 |
| O | 1.98901497916568 | 2.33680369524204 | 8.12508565806702 |
| O | 3.74340404813101 | 5.19698405087134 | 5.65045682570673 |
| C | 0.58268308676401 | 3.72005136233348 | 3.37021395310842 |
| H | 0.53004950629282 | 4.75205395731857 | 3.05328821895589 |
| C | 1.27750844469362 | 1.56039458365815 | 3.66924854880987 |
| H | 1.82579377993825 | 0.63669118613270 | 3.55448667917616 |
| C | -0.28780973210892 | 3.08836540577467 | 4.31582320501696 |
| H | -1.15862421441710 | 3.53860875753158 | 4.76843726950072 |
| C | 0.10077949042467 | 1.73452523599001 | 4.43078224348999 |
| H | -0.35533564582427 | 0.99468148163521 | 5.07198304684949 |
| C | 1.53530169728849 | 2.78010471424997 | 2.96566508375350 |
| H | 2.35822261692300 | 2.95691733049067 | 2.28753492475428 |
| C | 1.69147047998695 | 2.45892829068474 | 7.01022958610232 |
| C | 2.95106338400188 | 4.33652540081788 | 5.45678363272201 |
| C | 4.74387825768464 | 1.29706192262966 | 3.76358085500893 |
| H | 4.30907964215817 | 0.86943874501392 | 2.87189159081029 |
| C | 5.42872697363184 | 2.53703063404397 | 3.84120512970097 |
| H | 5.61220973394516 | 3.21264459128450 | 3.01907602052666 |
| C | 5.75312280393822 | 2.77407606329276 | 5.20149194407969 |
| H | 6.20390857807423 | 3.67210389761223 | 5.59577318496769 |
| C | 4.62925899779511 | 0.77599433853219 | 5.07445975299942 |

**ST4B**

| | | | |
|---|---|---|---|
| Mn | 1.74160153205388 | 3.16856213316855 | 5.32256405277369 |
| O | 2.07825247926624 | 1.70447467242658 | 8.30915773474335 |
| O | 3.74062897311918 | 5.34904916857286 | 5.72889724558455 |
| C | 1.06405159377667 | 3.68098817204733 | 3.36096827932152 |
| H | 1.30257563813434 | 4.62455117527912 | 2.89359268739628 |
| C | 1.13489794004178 | 1.48810866559664 | 4.01417308231945 |
| H | 1.45916886609587 | 0.46501189669046 | 4.14525064607058 |
| C | -0.06829547494971 | 3.42967746593738 | 4.20512743998915 |
| H | -0.83477608572254 | 4.14596721549812 | 4.45680400673925 |
| C | -0.02766658802373 | 2.06843698466829 | 4.58846992303024 |
| H | -0.72977215213534 | 1.56840592911701 | 5.24060392441406 |
| C | 1.77646301238081 | 2.46707264220981 | 3.21478299123225 |
| H | 2.68653952632134 | 2.33054379155889 | 2.65037543187332 |
| C | 1.99981321217097 | 2.18211262784492 | 7.29390312309568 |
| C | 3.13567249160456 | 4.32200664219738 | 5.54128073571782 |
| B | 4.14865008316842 | 3.04036957490577 | 5.11555708518810 |
| O | 4.84908957459668 | 3.08589984475177 | 3.94211778342601 |
| O | 4.63892501645775 | 2.13104229728866 | 6.01353471723455 |
| C | 6.06975524337987 | 2.32231494644414 | 4.15905235719333 |
| C | 5.65119737552078 | 1.35212450913950 | 5.30817870740545 |
| C | 6.45837895418763 | 1.63974403389567 | 2.86017851363831 |
| C | 7.14096473475385 | 3.32289831729305 | 4.57898229170001 |

| | | | |
|---|---|---|---|
| H | 4.09106860683904 | -0.11587699232073 | 5.35965187285006 |
| C | 5.25600220895529 | 1.69005825525501 | 5.96419473267079 |
| H | 5.27483595136336 | 1.61324770181106 | 7.04149394849019 |
| Be | 3.87991468472553 | 2.53076413148250 | 4.94342577485675 |
| C | -0.68138774395093 | 5.40580429090472 | 7.00070554454227 |
| H | -1.55782680370952 | 4.77849708495744 | 7.04749484681557 |
| C | 1.36201296167711 | 6.32367594642692 | 7.48743781601375 |
| H | 2.31504058295319 | 6.50729275881247 | 7.95867106443824 |
| C | -0.30805797013758 | 6.24306436562799 | 5.91998405143348 |
| H | -0.84895103796418 | 6.36349849560129 | 4.99320159331615 |
| C | 0.95481727886253 | 6.81092081796197 | 6.22187461357999 |
| H | 1.54486023617347 | 7.43013517673232 | 5.56378445530738 |
| C | 0.35315890834152 | 5.45458732574672 | 7.96793866890410 |
| H | 0.39416469253192 | 4.87326719390622 | 8.87701901288162 |
| Be | 1.00877474016044 | 4.90262058277054 | 6.26012395388072 |

| | | | |
|---|---|---|---|
| C | 4.95817169508691 | 0.09118106114616 | 4.80352466402153 |
| C | 6.76043366059807 | 1.00065948912745 | 6.28198206129847 |
| H | 6.73429908688207 | 2.39461569947908 | 2.12111819016800 |
| H | 7.32073091813096 | 0.98728555170072 | 3.02042770680424 |
| H | 5.63832352565221 | 1.04573595154238 | 2.45707079737020 |
| H | 8.10814628808617 | 2.83275704888269 | 4.70810025569006 |
| H | 7.23649862793147 | 4.08042479544815 | 3.79892439812391 |
| H | 6.87204786030122 | 3.82425864676803 | 5.51122186624795 |
| H | 5.66930816190814 | -0.58014423463686 | 4.31813550274184 |
| H | 4.51418294268161 | -0.43084626117075 | 5.65364686181326 |
| H | 4.16448897673778 | 0.33439841518946 | 4.09397466859947 |
| H | 6.37612279744674 | 0.32017025169250 | 7.04477348242942 |
| H | 7.57743711302682 | 0.49905841244031 | 5.75668451711485 |
| H | 7.15303617209536 | 1.88830940315457 | 6.77688122794735 |
| B | 1.19887914485462 | 4.88686311782125 | 6.20904087575833 |
| O | 1.03664092076949 | 4.98500478922450 | 7.58233978070969 |
| O | 0.81408902609851 | 6.05097892244065 | 5.57216975388766 |
| C | 0.74794278066235 | 6.37370143635590 | 7.89461404447057 |
| C | 0.15960948291771 | 6.89621070288328 | 6.54732120247442 |
| C | -0.21003815008183 | 6.42477370318835 | 9.07257254699285 |
| C | 2.07227666197175 | 7.03471345060220 | 8.26378961354411 |
| C | -1.34150707516162 | 6.64997309922401 | 6.41573893426742 |
| C | 0.48139308992292 | 8.34748334382278 | 6.23429122424272 |
| H | 0.28930257767160 | 6.04060939807457 | 9.96488931120176 |
| H | -0.51317052259201 | 7.45677375290563 | 9.26930240764797 |
| H | -1.10248458023568 | 5.82526260420909 | 8.89289450059444 |
| H | 1.92159733715295 | 8.07383818446521 | 8.56508725069931 |
| H | 2.51232307982967 | 6.49254198375322 | 9.10382459136415 |
| H | 2.77363563929094 | 7.00175893292528 | 7.42914939153346 |
| H | -1.91209053621157 | 7.30388759288654 | 7.07884116066262 |
| H | -1.64106951751794 | 6.85618126680513 | 5.38581197613616 |
| H | -1.59257033102762 | 5.61210622889012 | 6.64668879435763 |
| H | 0.02397882597057 | 8.62505930853456 | 5.28188802670981 |
| H | 0.07920541045686 | 9.00358506478602 | 7.01111929526263 |
| H | 1.55652698143954 | 8.50815297395491 | 6.15912281230647 |

**I3Be**

| | | | |
|---|---|---|---|
| Mn | 1.79966858136317 | 3.39863802068368 | 4.85480444623712 |
| O | 2.61171528928450 | 2.65947047475949 | 7.53920694515588 |
| C | 0.94153302037718 | 3.60182813163785 | 2.86808650249210 |
| H | 1.19678358531743 | 4.40775503513685 | 2.19202922625557 |
| C | 1.01582853301331 | 1.62714136240284 | 4.02944039665520 |
| H | 1.29859524069506 | 0.63803506056398 | 4.35878755142937 |
| C | -0.07886859548641 | 3.64843811476484 | 3.84677286686882 |
| H | -0.80238053169134 | 4.43907464089839 | 3.97293224066046 |
| C | -0.03542839586632 | 2.40777691746811 | 4.56324470401204 |
| H | -0.69258347121556 | 2.11658263493725 | 5.36957787381599 |
| C | 1.63655204908360 | 2.37435561377228 | 2.97566509584790 |
| H | 2.43269718410598 | 2.03586231531756 | 2.33061042353025 |
| C | 2.31704302833066 | 3.01112816539060 | 6.44314639741074 |
| C | 4.71788943060973 | 0.98371499542583 | 4.18491038853805 |
| H | 4.24224496812298 | 0.16944954059361 | 3.65779480305303 |
| C | 5.21417660153029 | 2.17420182654051 | 3.60142121080043 |
| H | 5.17864185921391 | 2.42902229043348 | 2.55240869453402 |
| C | 5.66263361815061 | 3.01927829014486 | 4.64672086731988 |
| H | 6.03848634677396 | 4.02543072418840 | 4.53158592150308 |
| C | 4.86672141360497 | 1.08969503749556 | 5.59103985109363 |
| H | 4.51425045112974 | 0.37829850165923 | 6.32307321449208 |
| C | 5.45180426226988 | 2.34551695171118 | 5.87538791139619 |
| H | 5.61661518984766 | 2.75430593138279 | 6.86104373847470 |
| Be | 3.81131563166046 | 2.52874274902032 | 4.88497171478848 |
| C | -0.88203806723562 | 5.19724123170032 | 6.93999765184043 |
| H | -1.80176843113378 | 4.77001668170947 | 6.56759114559429 |
| C | 1.06412416151350 | 5.48050078787816 | 8.11654437742151 |
| H | 1.89251607977504 | 5.29337306226132 | 8.78305351807511 |
| C | -0.19247236791405 | 6.30030951513888 | 6.38265087573708 |
| H | -0.49123197627523 | 6.85848062223263 | 5.50750220604017 |
| C | 1.01434242538752 | 6.47210599621765 | 7.10621674840279 |
| H | 1.78929543967224 | 7.19132540663099 | 6.88474592975873 |
| C | -0.10418387022110 | 4.69003523889213 | 8.01181529362722 |
| H | -0.32159692978336 | 3.80572015547612 | 8.59240037676213 |
| Be | 0.88466824598917 | 4.69431797553231 | 6.36749889037475 |

**I3B**

| | | | |
|---|---|---|---|
| Mn | 1.80602551715992 | 3.41274318439132 | 4.81848324570242 |
| O | 3.80493161727819 | 5.46772285771623 | 4.48258661645649 |
| C | 0.54695539483144 | 3.83145016558783 | 3.20857522435068 |
| H | 0.45336641420652 | 4.82030309097510 | 2.78596165512710 |
| C | 1.29116250857249 | 1.69822653728588 | 3.65917128470927 |
| H | 1.84059307076922 | 0.77078140293647 | 3.60653178104757 |
| C | -0.25142047849262 | 3.29430143061661 | 4.26788322043286 |
| H | -1.07765878270727 | 3.78773703358831 | 4.75682259521119 |
| C | 0.20601555591004 | 1.97999115605973 | 4.52162835664202 |
| H | -0.16037072805412 | 1.32817649140179 | 5.30336786067472 |
| C | 1.49586283947154 | 2.84954382768977 | 2.83403131826070 |
| H | 2.25528360901818 | 2.95542907128298 | 2.07420246791969 |
| C | 3.00724320010011 | 4.63986509361209 | 4.64647294530935 |
| B | 3.60510957679134 | 2.51125054649341 | 4.97404936143311 |
| O | 4.44711901733202 | 2.02154511926322 | 3.99915814509163 |
| O | 4.08485847876375 | 2.25507831274620 | 6.24625790815680 |
| C | 5.67557768795806 | 1.60714401181820 | 4.65498754428914 |
| C | 5.19249347905179 | 1.32393374903676 | 6.10987860221201 |
| C | 6.24043074254510 | 0.40041772088610 | 3.92690609182238 |
| C | 6.64361445392943 | 2.78319959986016 | 4.56694431449032 |
| C | 4.62095073591136 | -0.07959648810036 | 6.28799817760112 |
| C | 6.21812490424790 | 1.61747296703308 | 7.19023821145519 |
| H | 6.55662790771924 | 0.69386800361697 | 2.92348419339348 |
| H | 7.11213937841460 | 0.01064170471086 | 4.45955712009387 |
| H | 5.50056592390890 | -0.39482590829624 | 3.83597988967519 |
| H | 7.61838527686803 | 2.52547983812269 | 4.98676461686780 |
| H | 6.77821457532303 | 3.04865515135810 | 3.51638270134359 |
| H | 6.25278681862375 | 3.65666857585038 | 5.09302267351547 |
| H | 5.40947840339320 | -0.83479761417052 | 6.26243829236161 |
| H | 4.12063964593362 | -0.13412643082370 | 7.25706710742744 |
| H | 3.89006341889023 | -0.30958173581197 | 5.50935905758299 |
| H | 5.79399006995131 | 1.38619862484031 | 8.16979769584028 |
| H | 7.10677274374830 | 0.99679974350074 | 7.04642023511312 |
| H | 6.51634852577890 | 2.66551697829714 | 7.18255008579164 |
| B | 1.18284849623495 | 4.98523213101058 | 5.92048291618138 |
| O | 1.31048144993003 | 4.90527044115188 | 7.29555943303948 |
| O | 0.58322611678429 | 6.16291198427474 | 5.52857183887894 |
| C | 1.00023232732930 | 6.22019690841115 | 7.83394232803614 |
| C | 0.08100044770074 | 6.81465415471116 | 6.72437034695912 |
| C | 0.33973490545214 | 6.05555055032339 | 9.19084229329041 |
| C | 2.32465887023459 | 6.96482894799482 | 7.97294415322002 |
| C | -1.38024138209200 | 6.40173162636588 | 6.87674695833567 |
| C | 0.18891898538723 | 8.31884461357231 | 6.54729116554458 |
| H | 1.05884671403019 | 5.63678791689289 | 9.89819048655037 |
| H | 0.01105966255663 | 7.02626042169316 | 9.57209382866626 |
| H | -0.52079738317951 | 5.38873193448243 | 9.13937146479542 |
| H | 2.18086227127781 | 7.94484175180422 | 8.43322429539663 |
| H | 2.99126774591925 | 6.37808032185912 | 8.60825195809961 |
| H | 2.80632231782323 | 7.09953553989937 | 7.00195487990203 |
| H | -1.85034677198863 | 6.90991574654964 | 7.72142414035186 |
| H | -1.91886528831813 | 6.67420005657877 | 5.96655916187923 |
| H | -1.47171512328629 | 5.32299912103095 | 7.02273111339795 |
| H | -0.49160709037045 | 8.64354855899244 | 5.75699275516124 |
| H | -0.09178024269189 | 8.83063162311088 | 7.47187124952286 |
| H | 1.20091146811875 | 8.61633183591410 | 6.27362263538797 |

**T5Be**

| | | | |
|---|---|---|---|
| Mn | 1.75285958442823 | 3.40025429152900 | 4.95398141103855 |
| O | 2.52632101877886 | 2.63572380806481 | 7.64791577782365 |
| C | 0.92306929263616 | 3.61104464702764 | 2.96868482323023 |

**T5B**

| | | | |
|---|---|---|---|
| Mn | 1.87384372553383 | 3.42772932375429 | 4.65083954501384 |
| O | 3.86008917075559 | 5.51759699864206 | 4.52563885468164 |
| C | 0.70316049196039 | 3.94086197144286 | 3.00283839011719 |

| | | | |
|---|---|---|---|
| H | 1.17262977425553 | 4.42575938701993 | 2.30169947126943 |
| C | 1.01233143395573 | 1.62849097669837 | 4.11618300654059 |
| H | 1.30424702130468 | 0.64000700640586 | 4.43946964001504 |
| C | -0.11011151485015 | 3.63561945856140 | 3.93552457893369 |
| H | -0.84968681610383 | 4.41232647754207 | 4.05362236571469 |
| C | -0.05778492533384 | 2.38885487382181 | 4.64212456099674 |
| H | -0.72216883901877 | 2.08118062071058 | 5.43630197578709 |
| C | 1.63740076695374 | 2.39364656073854 | 3.07618185506000 |
| H | 2.44152952069725 | 2.06989922158110 | 2.43360200940793 |
| C | 2.24661392907532 | 2.99475268842568 | 6.54982160313659 |
| C | 4.62511861317793 | 0.95473721570405 | 4.23295400118730 |
| H | 4.15078802217795 | 0.19785281458090 | 3.62529794679566 |
| C | 5.17507000564478 | 2.17218535861959 | 3.76448218602396 |
| H | 5.18963057636270 | 2.50801514289662 | 2.73793779005672 |
| C | 5.60333972617220 | 2.91936092553048 | 4.88970119463397 |
| H | 6.00704587847387 | 3.92083070400735 | 4.86850627519487 |
| C | 4.71559852345945 | 0.94865191708757 | 5.64747493005546 |
| H | 4.31622758643808 | 0.19240380743219 | 6.30698601114135 |
| C | 5.32346113101600 | 2.16064331665010 | 6.05190367593297 |
| H | 5.45601904738917 | 2.48793022754041 | 7.07208487420881 |
| Be | 3.72676164849376 | 2.46915110977526 | 5.01532709170386 |
| C | -1.06206668102492 | 5.03059922074478 | 7.00121259364094 |
| H | -1.95447486336537 | 4.58759561646203 | 6.58350125113707 |
| C | 0.83261760859969 | 5.33661701690033 | 8.25326990060116 |
| H | 1.64356584133556 | 5.15345755321826 | 8.94183986554034 |
| C | -0.38983124725707 | 6.17267191304788 | 6.50362713239349 |
| H | -0.67635347643720 | 6.74954656323525 | 5.63652929728396 |
| C | 0.78380623433304 | 6.35931085525243 | 7.27546993794854 |
| H | 1.54382187665424 | 7.10719553283457 | 7.10188802534220 |
| C | -0.30496981846341 | 4.51283260761688 | 8.08208924301139 |
| H | -0.51359310005630 | 3.60393613328939 | 8.62688340197055 |
| Be | 0.74061784259830 | 4.59955704855329 | 6.47251380958431 |
| C | 3.38154110475938 | 5.84767373923743 | 4.26948035642216 |
| H | 2.58258583287706 | 5.37060542931822 | 4.87236840834678 |
| H | 4.23873230559347 | 6.01756045486012 | 4.92002545315810 |
| H | 2.97905120946039 | 6.79712897253225 | 3.91399620697257 |
| H | 3.67008576480760 | 5.22275453494428 | 3.42658352075549 |

| | | | |
|---|---|---|---|
| H | 0.63399308126501 | 4.95265333414052 | 2.63319171369714 |
| C | 1.41903748700223 | 1.78219277816741 | 3.37143356234886 |
| H | 1.97127675894069 | 0.85797199993740 | 3.29766155768150 |
| C | -0.15326692616250 | 3.34683770337042 | 3.98289722975715 |
| H | -1.00409238739543 | 3.81534772313471 | 4.45382635889680 |
| C | 0.28694437713814 | 2.01832614739378 | 4.18528056371739 |
| H | -0.12500853204464 | 1.32437231527569 | 4.90437073400989 |
| C | 1.67096091511416 | 2.97806859711833 | 2.62664355649703 |
| H | 2.47252617057882 | 3.12407025924736 | 1.91863100907330 |
| C | 3.06684048863393 | 4.67298059069996 | 4.60702880163272 |
| B | 3.68944544234062 | 2.55098429699646 | 4.81489259403050 |
| O | 4.52980033606188 | 2.09422537563118 | 3.82144493811810 |
| O | 4.18951384383753 | 2.27705097310589 | 6.07521739004417 |
| C | 5.76794374402280 | 1.67614100571372 | 4.45589512230972 |
| C | 5.30033295212061 | 1.35639015981999 | 5.90774946382436 |
| C | 6.33543624254717 | 0.49094892806948 | 3.69535630737039 |
| C | 6.72509172065555 | 2.86213088428555 | 4.38643451079017 |
| C | 4.73683675940444 | -0.05365348458914 | 6.05883898297208 |
| C | 6.33393242034816 | 1.63049217988829 | 6.98553537427375 |
| H | 6.63942196489281 | 0.80939129023087 | 2.69577117734884 |
| H | 7.21540427113173 | 0.09619936220160 | 4.21052070030739 |
| H | 5.60099545667612 | -0.30801428822161 | 3.59359085489350 |
| H | 7.70584038628251 | 2.60337628969898 | 4.79143786871279 |
| H | 6.84795826885592 | 3.15303990601246 | 3.34116136336934 |
| H | 6.33131148351252 | 3.71940631829557 | 4.93639095693197 |
| H | 5.52818196209076 | -0.80479530518912 | 6.01032019581837 |
| H | 4.24351638978824 | -0.13155781414671 | 7.02980191100064 |
| H | 4.00045858875808 | -0.26879529422439 | 5.28094387292105 |
| H | 5.91915183329681 | 1.37523295816332 | 7.96316700387252 |
| H | 7.22414846165232 | 1.01742412531391 | 6.82034563985565 |
| H | 6.62725444920775 | 2.67987150186474 | 6.99894231328323 |
| B | 1.18974589363002 | 4.94147626672529 | 5.80393469349166 |
| O | 1.26126809022750 | 4.81540458778202 | 7.17954655005089 |
| O | 0.59234850804898 | 6.12661796693283 | 5.42984021967490 |
| C | 0.90547562337915 | 6.10331741704391 | 7.75217524919690 |
| C | 0.02919128210601 | 6.72811145154728 | 6.62440088028489 |
| C | 0.18505691797206 | 5.87509619159100 | 9.06889852595632 |
| C | 2.20975295140936 | 6.86047913121577 | 7.98256312213887 |
| C | -1.43215112401839 | 6.29403341875032 | 6.69504647382484 |
| C | 0.12599588967109 | 8.23891160422352 | 6.50736376012987 |
| H | 0.87657171358125 | 5.43690756520298 | 9.79184393445991 |
| H | -0.17556805706555 | 6.82495032000376 | 9.47310378529453 |
| H | -0.66196035501148 | 5.19961486644691 | 8.95017925180673 |
| H | 2.02885833905135 | 7.82060858233443 | 8.47073659586645 |
| H | 2.85421773235526 | 6.25985615964890 | 8.62771826168427 |
| H | 2.73545004391291 | 7.03748497398868 | 7.04164998373504 |
| H | -1.94573665937431 | 6.76421998158996 | 7.53641142278501 |
| H | -1.93282937219917 | 6.59561977485056 | 5.77247287731729 |
| H | -1.51657665438246 | 5.20954203988333 | 6.79562916444239 |
| H | -0.52276945571868 | 8.58550087521960 | 5.69984202055660 |
| H | -0.20143951356864 | 8.71311593905921 | 7.43661341365851 |
| H | 1.14539663144549 | 8.55707812743035 | 6.29030941419224 |
| C | 1.14370162451704 | 1.23216383804766 | 7.62870179214900 |
| H | 1.39770322303169 | 0.87310747974960 | 8.62747870835754 |
| H | 0.07611291721858 | 1.45256290162853 | 7.58246229318347 |
| H | 1.39475456860599 | 0.46544294233804 | 6.89440017934845 |
| H | 1.71395031241275 | 2.13870643551662 | 7.42330015723530 |

**I4Be**

| | | | |
|---|---|---|---|
| Mn | 1.80714294164997 | 3.50168531904704 | 4.93184227935066 |
| O | 2.59315538464078 | 2.73876803261256 | 7.62700347062283 |
| C | 0.95471307636060 | 3.68416468152529 | 2.95705378095984 |
| H | 1.18257145120907 | 4.49495000309714 | 2.27829596909538 |
| C | 1.08406219413077 | 1.71359803228112 | 4.12057289459382 |
| H | 1.39046297988839 | 0.73073314435278 | 4.44758153162306 |
| C | -0.06872266425389 | 3.70272646217756 | 3.93479151809897 |
| H | -0.81839487065514 | 4.46971013606697 | 4.05260502435140 |
| C | 0.00826886708282 | 2.46390511503779 | 4.65112813328554 |
| H | -0.64516264657855 | 2.15289457245203 | 5.45309518899964 |
| C | 1.68772356615873 | 2.47764521588674 | 3.06782037896972 |
| H | 2.48867809414986 | 2.15901919857323 | 2.41853794518973 |
| C | 2.30951013883309 | 3.09339344496268 | 6.52811571874420 |
| C | 4.60979588836715 | 0.98442154589269 | 4.21753463613703 |
| H | 4.11305512696250 | 0.24572630841016 | 3.60540813454934 |
| C | 5.19491026729237 | 2.18819066735505 | 3.75583068129462 |
| H | 5.22020822194935 | 2.52890769844623 | 2.73104169486089 |
| C | 5.64665237455611 | 2.91486133868214 | 4.88510866766995 |
| H | 6.08114784694177 | 3.90356999344229 | 4.86932922697766 |
| C | 4.69952249992428 | 0.96785837866694 | 5.63142509193744 |
| H | 4.27777008854471 | 0.22021729217624 | 6.28683029324355 |
| C | 5.34343346598665 | 2.15909855609735 | 6.04266823056616 |
| H | 5.48589327886320 | 2.47638368237496 | 7.06461488039070 |
| Be | 3.75349177808608 | 2.52268218622678 | 5.00733327030498 |
| C | -1.06141426523540 | 5.02067311980912 | 6.98729546409490 |
| H | -1.93950792034649 | 4.56068023939438 | 6.55776115707137 |
| C | 0.81764374387465 | 5.35781347542016 | 8.25456489958178 |
| H | 1.62944753164504 | 5.18570857741331 | 8.94490928270615 |
| C | -0.41778727237900 | 6.18832610226633 | 6.51181552029899 |
| H | -0.71539780050066 | 6.77098857364225 | 5.65232276922972 |
| C | 0.74563701121882 | 6.39460426029089 | 7.29368754833455 |
| H | 1.48589412492385 | 7.16575536014743 | 7.13675414707825 |
| C | -0.29619975356414 | 4.50613237833442 | 8.06370951337220 |
| H | -0.48338361459706 | 3.58376359494364 | 8.59345525297157 |
| Be | 0.75573638933377 | 4.64303424519164 | 6.46022620617784 |
| C | 3.24517261536726 | 5.54395122847653 | 4.30867290470229 |
| H | 2.39393411536586 | 5.21995357923213 | 4.97640343043847 |
| H | 4.11492121454275 | 5.70682076983550 | 4.94233997375080 |
| H | 2.88762104425901 | 6.49557483261378 | 3.90997981300614 |
| H | 3.49924092600050 | 4.87947440714366 | 3.48958093536647 |

**I4B**

| | | | |
|---|---|---|---|
| Mn | 1.83460094658897 | 3.22441610071243 | 5.01999279425235 |
| O | 3.88559162231992 | 5.25375009535891 | 5.17744416743499 |
| C | 0.80053537939912 | 3.90847442853796 | 3.33980092200023 |
| H | 0.77959162715695 | 4.95078208236881 | 3.06243480666017 |
| C | 1.44665376981568 | 1.71045140968345 | 3.56709322884888 |
| H | 1.98044416980862 | 0.78052762107819 | 3.44589687811071 |
| C | -0.13561842026633 | 3.25576092710997 | 4.20435192174405 |
| H | -1.01204452357376 | 3.70143212141039 | 4.64950603900763 |
| C | 0.26115706537642 | 1.90314312242257 | 4.31235274277233 |
| H | -0.21851776427618 | 1.15689799396565 | 4.92927245567012 |
| C | 1.77191722413311 | 2.96031186173319 | 2.94894857171748 |
| H | 2.62716364812700 | 3.14646049032355 | 2.31830836901334 |
| C | 3.06507705908597 | 4.42861985613101 | 5.15131543956309 |
| B | 3.67898751533715 | 2.33311089730790 | 5.06360830941219 |
| O | 4.46130724205639 | 2.06612778141528 | 3.95242828582674 |
| O | 4.29554373785705 | 1.87998536062994 | 6.21817773145544 |
| C | 5.75929966050078 | 1.61743649633846 | 4.41372004255489 |
| C | 5.42714088715314 | 1.06249141229334 | 5.83064202873057 |
| C | 6.30207523520409 | 0.58824705099129 | 3.43728708124903 |
| C | 6.66817343316992 | 2.84268450214967 | 4.45117761208142 |
| C | 4.94351767770891 | -0.38516750384840 | 5.80757239155966 |
| C | 6.53338105903448 | 1.23085281792170 | 6.85748596562039 |
| H | 6.50934901125733 | 1.06813670358595 | 2.47819798379271 |
| H | 7.23562946428595 | 0.16184722694694 | 3.81448057087417 |
| H | 5.58967416682723 | -0.21978705067235 | 3.27080426445697 |
| H | 7.68751351645895 | 2.57079620064532 | 4.73415038650464 |
| H | 6.69328539065282 | 3.29088140596530 | 3.45567026873028 |
| H | 6.29452358517787 | 3.59089308483120 | 5.15293270006367 |
| H | 5.76053186993839 | -1.07397740482966 | 5.58214372858225 |
| H | 4.54094971157355 | -0.63622659681664 | 6.79127454511584 |
| H | 4.15308966642282 | -0.52460241417444 | 5.06627561062000 |
| H | 6.21340730315266 | 0.80923190209518 | 7.81304333025661 |
| H | 7.43436316990126 | 0.70188013941893 | 6.53491057984386 |
| H | 6.77868209617620 | 2.28168386624462 | 7.00973138191619 |
| B | 1.12080284544230 | 4.82143894917870 | 6.08405044085570 |
| O | 1.06731159533842 | 4.87329645574658 | 7.46741786706971 |
| O | 0.59353438218358 | 5.97539590783850 | 5.52887424070104 |
| C | 0.69958556270909 | 6.22248153712061 | 7.85067668189882 |
| C | -0.05478279844096 | 6.72469155145277 | 6.58405280802045 |
| C | -0.14121414802574 | 6.16587639281835 | 9.11419789891457 |
| C | 1.99804226940738 | 6.98064088249326 | 8.11106214405214 |
| C | -1.52944811217064 | 6.32970675385159 | 6.57200757423028 |

| | | | |
|---|---|---|---|
| C | 0.09416482632635 | 8.20917886627847 | 6.30015578043514 |
| H | 0.47074274546546 | 5.80704440739946 | 9.94474214140621 |
| H | -0.51158563189693 | 7.16275186355421 | 9.36847533021340 |
| H | -0.99205912597230 | 5.49455206918598 | 8.99846546797410 |
| H | 1.79983387280300 | 7.99474392169622 | 8.46507685370079 |
| H | 2.56512548614121 | 6.45078846564588 | 8.87935440782015 |
| H | 2.61138354998739 | 7.03595193576221 | 7.20920965514065 |
| H | -2.10228235563705 | 6.90775569600204 | 7.30042311196631 |
| H | -1.93698129371381 | 6.52588614842880 | 5.57783596411204 |
| H | -1.65429001111263 | 5.26712483083858 | 6.79299232199634 |
| H | -0.46925854910068 | 8.46828319252355 | 5.40084050689348 |
| H | -0.30209560611990 | 8.79679513385320 | 7.13282851817530 |
| H | 1.13758507950665 | 8.48136439930781 | 6.14279775685821 |
| C | 1.40080685625642 | 1.86276891698316 | 7.26405214396040 |
| H | 1.76153903648407 | 1.85578741771686 | 8.29372550067521 |
| H | 0.34378946309782 | 2.11044793070575 | 7.25089061060304 |
| H | 1.60164011245805 | 0.90233846593650 | 6.79831331234523 |
| H | 2.02593567241943 | 2.66978386840340 | 6.82571097393511 |

**T6Be**

| | | | |
|---|---|---|---|
| Mn | -0.27180404226989 | -0.04314076139820 | -0.47131736984737 |
| O | -0.08065589348270 | -1.27753278870602 | 2.15113470382145 |
| C | -0.66859581858645 | 0.29390790676921 | -2.55994105533291 |
| H | -0.20422596455033 | 1.08010111510386 | -3.13786552577135 |
| C | -1.04441551519465 | -1.70821329290591 | -1.50404419168645 |
| H | -0.93793643285959 | -2.72935240530493 | -1.16768059474108 |
| C | -1.86845774230932 | 0.41654226288666 | -1.82075424981909 |
| H | -2.50757545998570 | 1.28594936882662 | -1.78674945951629 |
| C | -2.10370187232884 | -0.83076340041632 | -1.16838722792434 |
| H | -2.94718242655518 | -1.06921951468270 | -0.53745872827100 |
| C | -0.13865281361944 | -1.00638982784342 | -2.35940910738390 |
| H | 0.73642406898294 | -1.41428832416289 | -2.84069707840263 |
| C | -0.09974624843815 | -0.74166292465478 | 1.09665040517352 |
| C | 2.45725864827560 | -2.70102955872066 | -0.85708666720960 |
| H | 2.00111478141550 | -3.40336495022661 | -1.53960125526606 |
| C | 3.16992093289966 | -1.53071182893410 | -1.22129618416749 |
| H | 3.35515553492429 | -1.18677459736917 | -2.22846050882555 |
| C | 3.51395788034359 | -0.84276441677316 | -0.03276337341179 |
| H | 4.00589953975905 | 0.11694719453133 | 0.02208268778964 |
| C | 2.35942202143847 | -2.73264748175889 | 0.55348633826116 |
| H | 1.80398539975173 | -3.45221938177236 | 1.13629812071766 |
| C | 3.00873820563297 | -1.58225972898129 | 1.06367937564947 |
| H | 3.03673002641300 | -1.28200595534483 | 2.10052058491361 |
| Be | 1.60400499906985 | -1.10903143600730 | -0.17142305449293 |
| C | -3.32580810625840 | 1.50057677398005 | 1.22251861724279 |
| H | -4.13753145633833 | 1.17358784166366 | 0.58904952463325 |
| C | -1.68300874394008 | 1.51569858260427 | 2.81940682545508 |
| H | -1.01689080915353 | 1.19150317427341 | 3.60481080109446 |
| C | -2.55144583634537 | 2.67275205772456 | 1.04207986802913 |
| H | -2.66448310861734 | 3.39053601594284 | 0.24274719296236 |
| C | -1.53588783024965 | 2.68111219708661 | 2.02891298200554 |
| H | -0.74096177356775 | 3.40784440050506 | 2.11338919717028 |
| C | -2.78963069084839 | 0.78595264669385 | 2.32243630590572 |
| H | -3.11148394130810 | -0.18512659967624 | 2.66848669906864 |
| Be | -1.46534357596663 | 1.06480656679892 | 0.95516112787638 |
| C | 1.25613170019941 | 1.55227468983785 | -0.61343167484491 |
| H | -0.03234355137035 | 1.42428116432139 | -0.07358715391468 |
| H | 1.91513722994619 | 1.62079213120625 | 0.25229640317083 |
| H | 0.97364154972108 | 2.57833100883959 | -0.88321529312993 |
| H | 1.82069919028245 | 1.16224695041947 | -1.45935873943859 |

**T6B**

| | | | |
|---|---|---|---|
| Mn | -0.38721892257256 | 0.23870121999339 | -1.27726060840773 |
| C | -0.38788446765898 | 1.05631212755796 | -3.29032523607544 |
| H | 0.26633964977551 | 1.85367485815399 | -3.61074566329243 |
| C | -1.66484035951162 | 1.22193528735848 | -2.70513820757306 |
| H | -2.16323885565133 | 2.15917075884602 | -2.51269202245038 |
| C | -2.16418409983509 | -0.06626233261982 | -2.38041560092248 |
| H | -3.11082566618694 | -0.27542021443726 | -1.90596075038356 |
| C | -1.18682055373517 | -1.02459066076880 | -2.76611683460675 |
| H | -1.25951041469492 | -2.09493181735305 | -2.64311824781758 |
| C | -0.08212964153615 | -0.32468466326320 | -3.32260503567215 |
| H | 0.82889568810639 | -0.77005747945288 | -3.68826669200754 |
| C | -0.77306684801801 | -0.87482896917198 | -0.00690072831034 |
| O | -1.11331472111159 | -1.66879703788208 | 0.76998446145487 |
| B | 1.15638589833448 | -1.02707583416800 | -0.78616707038329 |
| O | 1.99982608976301 | -0.95310772629328 | 0.30808022481713 |
| C | 2.81046852378373 | -2.15120728367745 | 0.34133755808181 |
| C | 2.78590827221773 | -2.59972181994411 | -1.14955820873088 |
| O | 1.50235972194960 | -2.09670966839595 | -1.59608866029265 |
| C | 4.18660063587515 | -1.79951069185445 | 0.87967930613699 |
| H | 4.10195172258136 | -1.49956090964996 | 1.92648526491249 |
| H | 4.63604124031358 | -0.97775590310961 | 0.32223206335992 |
| H | 4.85024507558678 | -2.66688769111778 | 0.82536132193611 |
| C | 2.11450045404631 | -3.13892324840928 | 1.27327722889998 |
| H | 1.98952757116422 | -2.66948666427835 | 2.25118532565061 |
| H | 1.12626283500404 | -3.40930417824794 | 0.89700226770888 |
| H | 2.70678883459711 | -4.04819490070879 | 1.39743731000782 |
| C | 3.85205950255487 | -1.91164802494820 | -1.99791960329592 |
| H | 3.83842887623376 | -0.82993496293961 | -1.84569479321072 |
| H | 3.64814964603412 | -2.11456436439381 | -3.05161752344201 |
| H | 4.85059742069019 | -2.28549744128122 | -1.76156285386450 |
| C | 2.82888629257734 | -4.10194676363645 | -1.36694740880852 |
| H | 2.80628088329594 | -4.31789692420899 | -2.43758132279548 |
| H | 3.75140965995473 | -4.51917466217951 | -0.95412363898599 |
| H | 1.97916532781908 | -4.59712557197503 | -0.89754356306710 |
| B | -1.33705584507445 | 1.26951783937422 | 0.22865416995866 |
| O | -2.62341813900135 | 1.75763079824703 | 0.07507766091775 |
| C | -3.14764481494870 | 2.03583213061285 | 1.39865518108597 |
| C | -1.84770503584036 | 2.29424819526409 | 2.21711803605465 |
| O | -0.88526627540006 | 1.46469210202651 | 1.52450666271047 |
| C | -3.89831195971450 | 0.78650091304424 | 1.85130217508330 |
| H | -3.22808873517348 | -0.07215051150589 | 1.92850315093111 |
| H | -4.67046505892939 | 0.55584348507875 | 1.11416956145125 |
| H | -4.38064618437196 | 0.94333689801453 | 2.81854464434159 |
| C | -4.09517580131224 | 3.21934463429171 | 1.31455026889826 |
| H | -4.97464531256377 | 2.94329382707202 | 0.72846414894858 |
| H | -4.42880264219878 | 3.50872030427528 | 2.31474827717054 |
| H | -3.62128351785090 | 4.07892060658846 | 0.84068667833254 |
| C | -1.91448803684780 | 1.86335271250971 | 3.67191784657415 |
| H | -2.10187201495131 | 0.79362365750852 | 3.76216280916332 |
| H | -2.70769298052759 | 2.40644479051354 | 4.19291651010232 |
| H | -0.96558856248431 | 2.08908094963591 | 4.16344389735803 |
| C | -1.35276660731288 | 3.73419841204752 | 2.11272454573165 |
| H | -0.34535570670272 | 3.78959136818948 | 2.53029545055502 |
| H | -1.99731608116783 | 4.41684523515851 | 2.67072499944881 |
| H | -1.31251901231969 | 4.06220581756244 | 1.07150335593577 |
| C | 1.38321617811682 | 1.51197996988282 | -1.00982003037031 |
| H | 1.84456963761820 | 1.46179201363896 | -0.02691728477795 |
| H | 2.06346221409074 | 1.08814852600992 | -1.74760236736373 |
| H | 1.24386034417234 | 2.56424211350079 | -1.27936858002309 |
| H | -0.03446817281057 | 1.54994327310445 | -0.58078643568137 |

**I5Be**

| | | | |
|---|---|---|---|
| Mn | 2.00840316532175 | 3.67981017300052 | 5.12000597512278 |
| O | 1.53701252521444 | 2.00746899553971 | 7.43904890277485 |
| C | 1.10942848324868 | 4.47909903817448 | 3.31676619139841 |
| H | 1.05667632387648 | 5.53112307074823 | 3.07725420301397 |
| C | 1.86461483425990 | 2.31462704716355 | 3.49058229877375 |
| H | 2.44095413255339 | 1.41690213586099 | 3.33434702690427 |
| C | 0.16233530449352 | 3.74767892039149 | 4.09789031983481 |
| H | -0.76544943298844 | 4.12739746612834 | 4.49688534996488 |
| C | 0.62676692448352 | 2.41013340818955 | 4.19036149127240 |
| H | 0.12495583888204 | 1.60017007215485 | 4.70008487522780 |
| C | 2.14093562512110 | 3.59937691776967 | 2.94604304884778 |
| H | 3.03051346703351 | 3.86834839645944 | 2.39453046897823 |
| C | 1.80272002685144 | 2.73260116017140 | 6.56099257888915 |
| C | 4.89684364205358 | 0.93561627749897 | 4.66271397473806 |
| H | 4.72460425713122 | 0.58234675792798 | 3.65557020927343 |
| C | 6.00170296148213 | 1.70782729946269 | 5.09720026818149 |
| H | 6.78166964214635 | 2.10393270164187 | 4.46119027826299 |
| C | 5.83127316574055 | 1.98522969723166 | 6.46386163476992 |
| H | 6.46363878615936 | 2.62103067955163 | 7.06830072789333 |
| C | 4.06190398642383 | 0.68692666031997 | 5.78474347271158 |

**I5B**

| | | | |
|---|---|---|---|
| Mn | 1.53896812645299 | 3.49413947206555 | 5.42008205677628 |
| O | 1.44121059272980 | 2.07094835825006 | 7.96198239813595 |
| C | 0.96403358816453 | 3.77477059046007 | 3.37906017678667 |
| H | 1.11134916678000 | 4.67828722040040 | 2.80603602213842 |
| C | 1.29795514239227 | 1.69373091787712 | 4.29700361192898 |
| H | 1.74058947162243 | 0.73941601021404 | 4.53609265178749 |
| C | -0.17043184963867 | 3.46550156015492 | 4.19240094070621 |
| H | -1.02011856614090 | 4.10656983550253 | 4.37076783496620 |
| C | 0.03124375110193 | 2.18657604808153 | 4.75596022966048 |
| H | -0.64990381649432 | 1.67128718586991 | 5.41739819370971 |
| C | 1.85287816126320 | 2.67426419600457 | 3.44631865422443 |
| H | 2.82834423790961 | 2.61612168587897 | 2.98522544655549 |
| C | 1.53806341278788 | 2.67571875290492 | 6.98291381357176 |
| C | 4.00970349717801 | 4.01568715487603 | 5.82171702198992 |
| H | 3.81366059292577 | 4.44269509112403 | 6.80282698824148 |
| H | 4.80033612023810 | 4.61094564723712 | 5.34969175107676 |
| H | 3.18519519091394 | 4.26712588434336 | 5.10237191447098 |
| H | 1.34898208847154 | 5.06320363999866 | 5.63566152519449 |
| B | 4.46113715327594 | 2.51602374795917 | 5.87084737081051 |
| O | 4.78429631357433 | 1.87101625761891 | 7.03778909186738 |

| | | | |
|---|---|---|---|
| H | 3.14018468299744 | 0.12406016244465 | 5.78802648050775 |
| C | 4.62663532007071 | 1.37046803157115 | 6.89583170485351 |
| H | 4.20972631856952 | 1.40398887575761 | 7.89142451356867 |
| Be | 3.91694142663289 | 2.56881357558120 | 5.48097863944160 |
| C | -0.43186935005615 | 4.59250420461939 | 7.91883813634599 |
| H | -0.93206444622070 | 3.63849627025941 | 7.99127757780039 |
| C | 1.13696353731873 | 6.24828803535608 | 8.15586558407982 |
| H | 2.03711293692508 | 6.77411086875635 | 8.43871405293833 |
| C | -0.77224956134948 | 5.65124493551597 | 7.04011910421789 |
| H | -1.59269674850405 | 5.65345356594519 | 6.33774390185117 |
| C | 0.19463481056080 | 6.67582863758600 | 7.19149942626065 |
| H | 0.25147069291073 | 7.58341823517124 | 6.60861599516428 |
| C | 0.75303216136969 | 4.96015499768822 | 8.60406980054405 |
| H | 1.30414196327650 | 4.33381330896269 | 9.28939736147392 |
| Be | 0.99723530128344 | 4.97497924380674 | 6.70307864106034 |
| C | 4.15614611695215 | 4.36445210390114 | 5.09486613624042 |
| H | 4.58486900809147 | 4.75044524702982 | 6.02329574330363 |
| H | 4.98928927950285 | 4.03111494469250 | 4.46426880571114 |
| H | 3.75078597895180 | 5.21610652536483 | 4.55562944434209 |
| H | 1.97146150122774 | 5.18530493460199 | 5.64669759345899 |

| | | | |
|---|---|---|---|
| O | 4.68619179873865 | 1.74052696476047 | 4.75691383885598 |
| C | 5.05884384136066 | 0.48340178512977 | 6.71080328313562 |
| C | 5.41617044799359 | 0.56315866048443 | 5.19036873332240 |
| C | 6.18339582101080 | -0.01562481825963 | 7.60158367138553 |
| C | 3.78237860037343 | -0.30251343284758 | 6.98906102994401 |
| C | 6.89398827183897 | 0.84572111673292 | 4.93626226310049 |
| C | 4.96022979018503 | -0.62856416815949 | 4.36684033350648 |
| H | 5.84251776544550 | -0.03659532795472 | 8.63884331116092 |
| H | 6.47095493753560 | -1.03084672429232 | 7.31544373028836 |
| H | 7.06046422047116 | 0.62818639071178 | 7.54048335387626 |
| H | 3.93366455978179 | -1.37024208705493 | 6.81695796026554 |
| H | 3.49663114201890 | -0.15338205247387 | 8.03158393743394 |
| H | 2.95953045627397 | 0.04331340079380 | 6.36236110133447 |
| H | 7.51169016436869 | -0.01941005015906 | 5.18566753963733 |
| H | 7.03062927991458 | 1.07493647109102 | 3.87742432985819 |
| H | 7.23806679782003 | 1.70179430540661 | 5.52084823719414 |
| H | 5.24781661855622 | -0.48237956129441 | 3.32334875363687 |
| H | 5.43706413512113 | -1.54361398436479 | 4.72790298574909 |
| H | 3.87899298840598 | -0.75659123928016 | 4.41164855966919 |
| B | 0.71378593292377 | 4.83860783557999 | 6.79501168392747 |
| O | -0.64986276347516 | 5.08958139023110 | 6.84021422309082 |
| O | 1.34978211947495 | 5.31971746609702 | 7.93341716026050 |
| C | -0.97036703599194 | 5.49876525165392 | 8.19192145668748 |
| C | 0.38523445324293 | 6.08921562011769 | 8.68819871465027 |
| C | -2.11848887097334 | 6.49215141304841 | 8.14508766282750 |
| C | -1.38978212453689 | 4.24550783323132 | 8.95612793609151 |
| C | 0.57552146657646 | 7.55248966985023 | 8.29736183343769 |
| C | 0.65500457052632 | 5.90079219739667 | 10.17119253316999 |
| H | -3.02179352104748 | 5.98963248755743 | 7.79191757638001 |
| H | -2.31754034518322 | 6.88893781126830 | 9.14435555075840 |
| H | -1.90256067593302 | 7.32341103923378 | 7.47409070394017 |
| H | -1.71532003524558 | 4.49080798586300 | 9.96941741977489 |
| H | -2.22464729675956 | 3.77791264139827 | 8.42952486485500 |
| H | -0.57297375077391 | 3.52415523399186 | 9.01495515443013 |
| H | -0.07851849275554 | 8.20754500785435 | 8.87706704317708 |
| H | 1.61156812252809 | 7.83609861444367 | 8.49415939563684 |
| H | 0.37254933406866 | 7.70463492761361 | 7.23495983141573 |
| H | 1.61665424924859 | 6.35099611373168 | 10.42749389013093 |
| H | -0.12139233408475 | 6.39186743947711 | 10.76423393426332 |
| H | 0.68762298544710 | 4.84486711456820 | 10.43869678313830 |

**T7Be**

| | | | |
|---|---|---|---|
| Mn | 1.83206949314095 | 3.31611787660646 | 4.76232909201586 |
| O | 1.02451889061808 | 1.60900710689888 | 6.96905500452384 |
| C | 0.96008218135099 | 4.09232807203822 | 2.92828507598522 |
| H | 0.65494233833783 | 5.12067199980090 | 2.79524143776931 |
| C | 2.25866499878021 | 2.19783769841932 | 3.02063133314446 |
| H | 3.07144146705091 | 1.50665938082397 | 2.86381355168026 |
| C | 0.16269516285679 | 3.05257622347418 | 3.49089135195407 |
| H | -0.86910337381407 | 3.13058064654806 | 3.79892429399770 |
| C | 0.96759889452707 | 1.87925199699451 | 3.53026928393003 |
| H | 0.65230796983032 | 0.90798524826075 | 3.88422583298741 |
| C | 2.23213531251434 | 3.57299290579832 | 2.63756074048593 |
| H | 3.06922690472740 | 4.13456425345309 | 2.24766898143883 |
| C | 1.41570665746123 | 2.32330217755321 | 6.13561528904310 |
| C | 5.09889748876880 | 1.06774414769291 | 4.79989710238524 |
| H | 5.20795296373203 | 0.79340922696005 | 3.76012171116689 |
| C | 5.93172800250359 | 1.96158857993802 | 5.51739251852006 |
| H | 6.74773110868690 | 2.53530776968932 | 5.09989036554034 |
| C | 5.43256545952623 | 2.06651866288168 | 6.82799641290653 |
| H | 5.80308980198875 | 2.72895711489330 | 7.59832830896634 |
| C | 4.10166943833521 | 0.58262856614157 | 5.68722369548465 |
| H | 3.31032430809141 | -0.11161812501146 | 5.44462322568521 |
| C | 4.29248185487723 | 1.22903289021664 | 6.93804150693123 |
| H | 3.67129904926575 | 1.10199311593923 | 7.81245775778814 |
| Be | 3.72513839431756 | 2.45626451333110 | 5.45541845739687 |
| C | -0.03219110314758 | 5.05099768641369 | 8.47322218123616 |
| H | -0.55025704870549 | 4.12915489263792 | 8.69253510771340 |
| C | 1.65535633366622 | 6.56930880219982 | 8.12859795883347 |
| H | 2.63941663064631 | 7.00679520763553 | 8.04848329090320 |
| C | -0.59428984691587 | 6.19636784919968 | 7.85130175244942 |
| H | -1.61955324473593 | 6.30455137960533 | 7.53007002367302 |
| C | 0.44956299427134 | 7.13615651845217 | 7.63801931221262 |
| H | 0.35522753625954 | 8.08374044420846 | 7.12883860815698 |
| C | 1.35645968036937 | 5.28150966240408 | 8.64358085129560 |
| H | 2.07428344157780 | 4.56453013020482 | 9.01366266743398 |
| Be | 0.81780958334288 | 5.47477799143481 | 6.85182653216407 |
| C | 3.77975027335563 | 4.30889372140711 | 5.20929393566869 |
| H | 4.10421747401331 | 4.56750724599288 | 6.22231841377837 |
| H | 4.67445521307126 | 4.25492816060571 | 4.58011696100202 |
| H | 3.22668047184673 | 5.17172585933906 | 4.84170502327278 |
| H | 1.10116143360798 | 4.88204597891506 | 5.61913698847596 |

**T7B**

| | | | |
|---|---|---|---|
| Mn | 1.64219003464897 | 3.33006993727242 | 5.30804994885164 |
| O | 1.39927248510950 | 1.87997903322193 | 7.83536961912846 |
| C | 1.13401842580998 | 3.69244132903119 | 3.27307398048232 |
| H | 1.26350731693882 | 4.62269434605328 | 2.73956866926586 |
| C | 1.51698682950284 | 1.57996577275209 | 4.11322564778086 |
| H | 1.98678784058767 | 0.63238701701653 | 4.32563100232012 |
| C | -0.01976704544757 | 3.29522760891569 | 4.01814721040059 |
| H | -0.90599880867501 | 3.89052422687330 | 4.18517468583332 |
| C | 0.21002833682301 | 1.99928809869733 | 4.53133259387542 |
| H | -0.47899757247207 | 1.42613992803782 | 5.13460370641088 |
| C | 2.06943599814782 | 2.62595530478933 | 3.34057776136196 |
| H | 3.06609999681180 | 2.62823645324866 | 2.92309364776370 |
| C | 1.55640542703507 | 2.49786378835856 | 6.86970346831328 |
| C | 4.02288825865571 | 4.03049526857568 | 5.82347741919867 |
| H | 3.80972837032646 | 4.45720786310844 | 6.80141321492508 |
| H | 4.81049494810088 | 4.63151638760996 | 5.35371550456725 |
| H | 3.20000879472739 | 4.28020646006727 | 5.09904441982615 |
| H | 1.26267098485615 | 4.99789819469469 | 5.72026943472832 |
| B | 4.50210546396173 | 2.54009835994917 | 5.88807459855137 |
| O | 4.77661039011891 | 1.89359660515434 | 7.06897749033149 |
| O | 4.84778375809525 | 1.79091244350274 | 4.78524409655568 |
| C | 5.13310066950628 | 0.52499690975704 | 6.74680364136230 |
| C | 5.59626563943160 | 0.64288935499480 | 5.25770606481850 |
| C | 6.21037502646569 | 0.05963777208459 | 7.71158620522757 |
| C | 3.87608162666647 | -0.32067858823754 | 6.92008652288354 |
| C | 7.07484913285184 | 0.99372736908310 | 5.11669151832919 |
| C | 5.25814788110035 | -0.55691649692661 | 4.38963251260977 |
| H | 5.79477144675472 | 0.00530862650374 | 8.72005563679716 |
| H | 6.56366617060103 | -0.93678572555106 | 7.43306714483336 |
| H | 7.05984303395810 | 0.74227646405126 | 7.72587968701071 |
| H | 4.08784140269919 | -1.37748192852237 | 6.74403335657312 |
| H | 3.50969385331728 | -0.20252327967247 | 7.94107751214409 |
| H | 3.08642560343352 | 0.00087100607342 | 6.24026553134374 |
| H | 7.71116470061170 | 0.15326703304228 | 5.40168340442185 |
| H | 7.27789119004400 | 1.24330477522442 | 4.07312303706166 |
| H | 7.33678856015288 | 1.85627658196451 | 5.73365299421357 |
| H | 5.61478115629188 | -0.38210224731786 | 3.37209893770834 |
| H | 5.74885330043161 | -1.45476968046636 | 4.77458415219683 |
| H | 4.18357394700710 | -0.73372463539410 | 4.35218013772782 |
| B | 0.67131996636273 | 5.13910006186934 | 6.81612005585591 |
| O | -0.67677865231412 | 5.32154589738129 | 6.86903322272757 |
| O | 1.33671380070557 | 5.46847956868570 | 7.95836496492829 |
| C | -0.99815889116612 | 5.55732158079065 | 8.27323439759733 |
| C | 0.35789453623161 | 6.09132475698222 | 8.84227971170200 |
| C | -2.15094747807424 | 6.54138858221479 | 8.35034998508004 |
| C | -1.40705931249028 | 4.20992449465879 | 8.85853428080673 |
| C | 0.52423659900859 | 7.59845251256519 | 8.68382966341639 |
| C | 0.65615380315421 | 5.66948897559468 | 10.26946633090419 |
| H | -3.04983278832422 | 6.08138934412382 | 7.93466743387211 |
| H | -2.35159268786039 | 6.80436038788737 | 9.39215597493343 |
| H | -1.93948713667945 | 7.45301822767596 | 7.79206535239524 |
| H | -1.72554075185181 | 4.31628028774664 | 9.89751583681781 |
| H | -2.24420936757330 | 3.81657935150372 | 8.27844732402350 |
| H | -0.58871025316605 | 3.48925147388245 | 8.81372116248007 |
| H | -0.13727565754907 | 8.13939694739716 | 9.36348324959701 |
| H | 1.55639395522245 | 7.86466835594231 | 8.91990982102037 |
| H | 0.31165220866070 | 7.91623801195513 | 7.66071581829634 |
| H | 1.61386089972379 | 6.09162332385310 | 10.58091360922052 |

| | | | |
|---|---|---|---|
| H | -0.11905401807978 | 6.04377710164104 | 10.94313744772037 |
| H | 0.71041567184251 | 4.58513648594242 | 10.36139682111834 |

### I6Be

| | | | |
|---|---|---|---|
| Mn | 2.36302998425925 | 3.70511370560284 | 4.86245779241945 |
| O | 1.72079652311921 | 3.30658196422530 | 7.66696165733881 |
| C | 0.98103991515081 | 4.36021484692734 | 3.35472025103011 |
| H | 0.77984511758527 | 5.40404287027688 | 3.15579730102324 |
| C | 1.95637051765854 | 2.28640058933178 | 3.34448801167979 |
| H | 2.59321924328951 | 1.45098150440422 | 3.09571751099524 |
| C | 0.29561098484786 | 3.56201185110674 | 4.29775427521850 |
| H | -0.53282377994839 | 3.87505980251895 | 4.91566797000252 |
| C | 0.90388090588995 | 2.26959642621797 | 4.29515968634177 |
| H | 0.60853747406698 | 1.42781174753565 | 4.90410129998987 |
| C | 2.00352393173554 | 3.57998359596890 | 2.75306710523300 |
| H | 2.69612674573428 | 3.91098994488837 | 1.99282889276924 |
| C | 2.04170014353417 | 3.51051510324340 | 6.56861931675563 |
| C | 5.04820415846730 | 0.94930813717569 | 4.68850877805269 |
| H | 5.13209569875452 | 0.72760055107449 | 3.63454745770940 |
| C | 5.91206974427597 | 1.78926371573970 | 5.43066026362000 |
| H | 6.76293440622617 | 2.32718295140834 | 5.03880323503831 |
| C | 5.39812288063907 | 1.89104205879092 | 6.74677388726070 |
| H | 5.79301739076255 | 2.51358418951628 | 7.53640441938440 |
| C | 4.00859872045365 | 0.51835716372316 | 5.55075050079611 |
| H | 3.15479094299899 | -0.07832792675750 | 5.26609040720817 |
| C | 4.22531399844260 | 1.10022820618942 | 6.82180247171710 |
| H | 3.56508439302138 | 1.02730704421997 | 7.67301919036925 |
| Be | 4.08834844445406 | 2.45180921067210 | 5.43912182729955 |
| C | 4.22299062100345 | 4.33877411719759 | 4.85243816311864 |
| H | 4.91958045585636 | 4.54502453921370 | 5.67041583339726 |
| H | 4.76224201325768 | 4.44861234732750 | 3.90812211715379 |
| H | 3.47954842446276 | 5.20532974225992 | 4.91650037707664 |

### I6B

| | | | |
|---|---|---|---|
| Mn | 2.35066718858198 | 3.62673051185674 | 5.03644181487746 |
| O | 2.10182393392012 | 3.74707059839768 | 7.93629582791692 |
| C | 1.21215866738570 | 3.92578635958104 | 3.23293586331856 |
| H | 1.42094148197528 | 4.71650901949026 | 2.52495452622074 |
| C | 1.28746657473988 | 1.92038744879465 | 4.34690516263063 |
| H | 1.52584710209518 | 0.90078552486113 | 4.60413762498237 |
| C | 0.37309630427640 | 4.03651597997171 | 4.38161293251494 |
| H | -0.20934418542806 | 4.89764414294356 | 4.67364875510653 |
| C | 0.41118656293762 | 2.78356998814201 | 5.05336100981193 |
| H | -0.11948436351669 | 2.53792719736363 | 5.96207171749423 |
| C | 1.76947424288224 | 2.63362787826572 | 3.20974270020805 |
| H | 2.48390029907829 | 2.25927182396907 | 2.49151532792039 |
| C | 2.26697066226315 | 3.73819501718902 | 6.79602605134849 |
| C | 4.28266277571497 | 3.95786181435927 | 4.62012794860247 |
| H | 5.19040626195835 | 4.09789725152636 | 5.21179261483196 |
| H | 4.55546350974112 | 3.85220077549825 | 3.57078738562155 |
| H | 3.73842096540461 | 4.93489115610548 | 4.76015133156160 |
| B | 3.97102285663376 | 2.36146288489376 | 5.38085825577012 |
| O | 4.56676688621285 | 2.14638889792888 | 6.62520786818952 |
| O | 4.21678597793464 | 1.27999985764055 | 4.53630404080823 |
| C | 4.90151534030216 | 0.74605475036465 | 6.70662414208873 |
| C | 5.12787752475676 | 0.38184071748717 | 5.20912727939066 |
| C | 6.12215794985158 | 0.57809540630762 | 7.59519964136752 |
| C | 3.70185389006444 | 0.02572695228101 | 7.31959826945290 |
| C | 6.53459296211000 | 0.72013262931933 | 4.72254141746113 |
| C | 4.77413742607515 | -1.04880303974976 | 4.84075884719167 |
| H | 5.86681872107302 | 0.85167187902423 | 8.62134140225262 |
| H | 6.45496354683029 | -0.46350105539373 | 7.59165935187146 |
| H | 6.94586707248250 | 1.21106499456175 | 7.26545946892176 |
| H | 3.91003516531864 | -1.03527102138174 | 7.47403565446261 |
| H | 3.47828363018225 | 0.48071469625112 | 8.28665141060174 |
| H | 2.81826663628653 | 0.122226944997351 | 6.68481974004213 |
| H | 7.27583458084279 | 0.03574089223426 | 5.14097582559275 |
| H | 6.56068374260375 | 0.63347216162163 | 3.63413016126114 |
| H | 6.81017929928575 | 1.74165571167030 | 4.99428015678014 |
| H | 4.97042636124834 | -1.21325374646613 | 3.77878447838216 |
| H | 5.38496259775277 | -1.75165493546164 | 5.41386136089603 |
| H | 3.72250984841868 | -1.26118057142252 | 5.03317263224574 |

### T8Be

| | | | |
|---|---|---|---|
| Mn | 2.34201373955422 | 3.78646407991472 | 4.74550720122978 |
| O | 1.68382871894288 | 3.28602243106870 | 7.53006874843830 |
| C | 1.06731754670777 | 4.32815982409333 | 3.07955764666665 |
| H | 0.92584527020922 | 5.34880641066379 | 2.75010007347869 |
| C | 1.96501459088449 | 2.23940110173977 | 3.36550650237278 |
| H | 2.56329007107349 | 1.35310128192363 | 3.22696562739831 |
| C | 0.31101720972741 | 3.67771922275520 | 4.08485135995053 |
| H | -0.53956358491700 | 4.08395843524869 | 4.61172093556319 |
| C | 0.86942970074725 | 2.37363510285668 | 4.25768936400396 |
| H | 0.51296100626211 | 1.61791474073750 | 4.94254586107439 |
| C | 2.08292558509382 | 3.45658688336082 | 2.63014749546306 |
| H | 2.82739274124156 | 3.67985614017381 | 1.87987241942068 |
| C | 2.02819721689904 | 3.52045302722432 | 6.44519499769534 |
| C | 4.92936597659661 | 0.91699166946490 | 4.70676156136280 |
| H | 4.95727854423016 | 0.60598798788153 | 3.67246755890711 |
| C | 5.87116382548905 | 1.75845355246725 | 5.34448689450841 |
| H | 6.72847804773734 | 2.21814154026231 | 4.87479037153954 |
| C | 5.42350854513353 | 1.99177745382796 | 6.66587912475757 |
| H | 5.88714760678001 | 2.64924524173141 | 7.38703898862755 |
| C | 3.91206801393633 | 0.60732224506084 | 5.64523911228025 |
| H | 3.01759271347639 | 0.03559722576931 | 5.44670357303261 |
| C | 4.21606123590860 | 1.27386443354597 | 6.85302689263913 |
| H | 3.59602103726806 | 1.29839124146845 | 7.73629222425381 |
| Be | 4.07131687411014 | 2.53967563257298 | 5.36219663706328 |
| C | 4.32747946561801 | 4.32983647782087 | 4.63800572777543 |
| H | 5.07352380073388 | 4.47683960897008 | 5.42477137053452 |
| H | 4.85817350014587 | 4.22660876778321 | 3.68841210883288 |
| H | 3.79250475093668 | 5.29884659862577 | 4.59504572829597 |
| C | 1.49611713249168 | 6.36796618632472 | 5.52946685600581 |
| O | 0.52167432746348 | 6.70329078783132 | 5.98709912356694 |

### T8B

| | | | |
|---|---|---|---|
| Mn | 1.98776711745563 | 3.84774219895717 | 5.07316122580109 |
| O | 1.96200618030135 | 2.99645869244751 | 7.87696384196331 |
| C | 1.22292933547508 | 3.84869884708502 | 3.07957893514527 |
| H | 1.37396114999252 | 4.61770366364398 | 2.33641586291454 |
| C | 1.48824710485747 | 1.92697795131833 | 4.32706010528841 |
| H | 1.87315067218665 | 0.98652105969830 | 4.68782266562192 |
| C | 0.15236932019437 | 3.78044990825003 | 4.02505514593757 |
| H | -0.64114631529110 | 4.50470738032229 | 4.14163160980148 |
| C | 0.31154067413044 | 2.60333694800420 | 4.78992352584172 |
| H | -0.34308568629223 | 2.26962537463105 | 5.58188957441377 |
| C | 2.03415205681832 | 2.70112958876632 | 3.27785795161086 |
| H | 2.95290931241541 | 2.47555523693209 | 2.75585417583891 |
| C | 2.03948826183215 | 3.37463381230554 | 6.79028279010905 |
| C | 4.52676236718575 | 4.17387183762609 | 5.08623283796340 |
| H | 4.57516130528775 | 4.82050111926758 | 5.96007612504790 |
| H | 5.29512989889851 | 4.50519761996387 | 4.37776645028641 |
| H | 3.62756931830641 | 4.42863261397161 | 4.46344545253106 |
| B | 4.78528981962656 | 2.66626744128109 | 5.43168327059880 |
| O | 5.15115177324810 | 2.24595837980017 | 6.68642702409547 |
| O | 4.82802230864495 | 1.65355717384107 | 4.50095314799923 |
| C | 5.25590820720198 | 0.79850364261045 | 6.65141370960736 |
| C | 5.47629444362307 | 0.51711876704526 | 5.12896535495938 |
| C | 6.40460298564960 | 0.37042778920489 | 7.54882316553010 |
| C | 3.94116286726799 | 0.23905742971397 | 7.18348129696222 |
| C | 6.94516106317241 | 0.56824692833232 | 4.71775739751836 |
| C | 4.83388516773950 | -0.76078192581786 | 4.61811418165068 |
| H | 6.16050017104804 | 0.60580801579900 | 8.58689949826780 |
| H | 6.56420534319317 | -0.70833009775888 | 7.47161412847384 |
| H | 7.33092556962739 | 0.88211128702732 | 7.28863211562335 |
| H | 3.97192639305958 | -0.85134050537415 | 7.23493051662057 |
| H | 3.77133105645432 | 0.63108773408565 | 8.18754334592818 |
| H | 3.10022346270383 | 0.54003792574099 | 6.55791024261888 |
| H | 7.49533726388921 | -0.29100055298821 | 5.10671250020237 |
| H | 7.00586861389144 | 0.55350329040174 | 3.62757151465260 |
| H | 7.42410454307723 | 1.48174702320044 | 5.07719030012299 |
| H | 5.03679763066058 | -0.86880697109495 | 3.55035662769589 |
| H | 5.25175165578596 | -1.62856032692616 | 5.13514572093292 |
| H | 3.75411998772944 | -0.75317539087771 | 4.76440417802454 |
| C | 1.69960815080130 | 6.00075457310236 | 5.49841225852502 |
| O | 1.44753351953123 | 7.08090275223977 | 5.71279615326545 |

### I7Be

| | | | |
|---|---|---|---|
| Mn | 2.10532513349945 | 3.70452220339764 | 4.88446182741848 |
| O | 1.52185861289016 | 2.29624835119975 | 7.35420419658621 |
| C | 1.37021274190419 | 4.39019252050497 | 2.98048788820797 |
| H | 1.45880383849210 | 5.40516811922668 | 2.62218408901325 |
| C | 1.81414334483456 | 2.17072695133690 | 3.39914819553493 |
| H | 2.27526253192194 | 1.19626490797772 | 3.37715195083984 |
| C | 0.30491602891238 | 3.86743521306282 | 3.77899119806862 |
| H | -0.56066129299598 | 4.41463367108300 | 4.12212166431469 |
| C | 0.57784533078238 | 2.50311131692543 | 4.02895525721499 |
| H | -0.04237232021002 | 1.82833759770154 | 4.60099581150762 |
| C | 2.28597114986141 | 3.34120357953357 | 2.75203576040851 |
| H | 3.22059604520582 | 3.42747287430907 | 2.21681232217500 |
| C | 1.85495604131789 | 2.85057743813423 | 6.39343005274827 |
| C | 4.79921056484884 | 0.91905918962648 | 4.78018649107415 |
| H | 4.62351908706979 | 0.48564332882780 | 3.80642012473066 |
| C | 5.84155255381266 | 1.81748115158674 | 5.11176408659953 |

### I7B

| | | | |
|---|---|---|---|
| Mn | 1.93319216140442 | 3.93572568524167 | 5.06572035934296 |
| O | 2.08651516655322 | 3.35905173947812 | 7.93573429807952 |
| C | 1.04350297738371 | 3.84472006042087 | 3.11949639334004 |
| H | 1.15458800287685 | 4.58283772511803 | 2.33939691748711 |
| C | 1.36317143591591 | 1.97163111823538 | 4.42266685794068 |
| H | 1.76268515127843 | 1.04312125449858 | 4.79686510005763 |
| C | 0.03795297480182 | 3.83340288885316 | 4.13790373177162 |
| H | -0.73786771986912 | 4.57305533222832 | 4.27169676920353 |
| C | 0.23434526330935 | 2.68534318522344 | 4.93840025065836 |
| H | -0.36769284147189 | 2.39523759531303 | 5.78704561650881 |
| C | 1.84568260811299 | 2.69251031171452 | 3.30700509683409 |
| H | 2.72416727786124 | 2.43257800011012 | 2.73401763608801 |
| C | 2.08896567616482 | 3.59057912140139 | 6.81044575476702 |
| C | 4.47577069222956 | 4.08843978580396 | 4.92948872811151 |
| H | 4.64064545399322 | 4.78137179369847 | 5.75204535750414 |
| H | 5.19796254866076 | 4.32355389584045 | 4.13863379817762 |

| | | | |
|---|---|---|---|
| H | 6.57663504705459 | 2.21321209192161 | 4.42625538085750 |
| C | 5.67415779176975 | 2.20463464915218 | 6.45816931868772 |
| H | 6.26664021298407 | 2.93683915646344 | 6.98716902277218 |
| C | 4.01056249565172 | 0.71746804578273 | 5.93986436018924 |
| H | 3.10406042774208 | 0.13362598561264 | 5.99813610363449 |
| C | 4.54143627707000 | 1.52255458545279 | 6.97221266565772 |
| H | 4.12199115418813 | 1.64355552029544 | 7.95941986943935 |
| Be | 4.04623678056849 | 2.61676121322095 | 5.42211507794635 |
| C | 4.18876214706954 | 4.42225880479498 | 4.76429479850516 |
| H | 4.69284934284050 | 4.82394488409057 | 5.64853930525669 |
| H | 4.96387947357947 | 4.00333550038719 | 4.11391970022774 |
| H | 3.82207589072213 | 5.28288325253984 | 4.20988317484337 |
| C | 1.78793342222607 | 5.22759522301367 | 5.75273211921798 |
| O | 1.54128724438551 | 6.20479777283729 | 6.30263970732119 |

| | | | |
|---|---|---|---|
| H | 3.54867052970283 | 4.39291184107620 | 4.36796833738346 |
| B | 4.67807909698429 | 2.59273992737326 | 5.35394470637051 |
| O | 5.09061312629329 | 2.23638745105256 | 6.61353508174042 |
| O | 4.60926979111737 | 1.51998666259566 | 4.49570328191517 |
| C | 5.11501116475881 | 0.78629802721165 | 6.67439609069151 |
| C | 5.23094052399747 | 0.39144086166091 | 5.16545239797083 |
| C | 6.28915814001683 | 0.35269267022371 | 7.53527967757255 |
| C | 3.80474941941631 | 0.34284353652138 | 7.31646128334736 |
| C | 6.67347065155617 | 0.33012156354693 | 4.67172532217203 |
| C | 4.49229941650568 | -0.87881596532996 | 4.78183146997769 |
| H | 6.11955889052270 | 0.66986073991475 | 8.56636471225676 |
| H | 6.38556759072425 | -0.73618214559951 | 7.52394779294367 |
| H | 7.22464894550293 | 0.79182893033476 | 7.18955322619040 |
| H | 3.77689335903000 | -0.74152982042460 | 7.44219027042158 |
| H | 3.71812565380451 | 0.80961549298543 | 8.29909854100911 |
| H | 2.94509704445737 | 0.65047653784656 | 6.71962648231485 |
| H | 7.19877772128508 | -0.53068246407445 | 5.09034369653008 |
| H | 6.66875174889773 | 0.23701348485192 | 3.58376801194164 |
| H | 7.22137584877133 | 1.23703631127079 | 4.93717827099797 |
| H | 4.62022750902888 | -1.06615591021625 | 3.71345921719117 |
| H | 4.89835836624295 | -1.73309860801221 | 5.32973879149240 |
| H | 3.42611467521526 | -0.80392872779906 | 4.99345916201719 |
| C | 1.69049481483510 | 5.67846360298164 | 5.35085922486153 |
| O | 1.44365914212714 | 6.78881650682814 | 5.51595228481673 |

## T9Be

| | | | |
|---|---|---|---|
| Mn | 1.61038997712420 | 3.90624686646904 | 4.89329861077798 |
| O | 0.77908998925187 | 2.60419882907857 | 7.37864784966878 |
| C | 1.56605099629167 | 4.20569298768214 | 2.76437725736933 |
| H | 1.92498456662835 | 5.08078361459508 | 2.24374249293754 |
| C | 1.47442160694846 | 2.10857176338437 | 3.71713341983895 |
| H | 1.75026321791959 | 1.11819516558548 | 4.04686146837383 |
| C | 0.24588162593443 | 4.00671618294699 | 3.27942624144230 |
| H | -0.56796977287282 | 4.71532054570356 | 3.22974360372189 |
| C | 0.18808816970797 | 2.72228212838316 | 3.86531549981116 |
| H | -0.67723883864183 | 2.27908590013207 | 4.33585501010362 |
| C | 2.30315355638936 | 3.02821692431868 | 3.03733227445634 |
| H | 3.34977850291734 | 2.87950872756693 | 2.81402023000612 |
| C | 1.14870728782010 | 3.14516775325307 | 6.43136695638334 |
| C | 4.89166481333432 | 0.92827815815229 | 4.74022481211976 |
| H | 4.35539856102199 | 0.64391157551170 | 3.84703404953605 |
| C | 6.19526258786384 | 1.48574985656435 | 4.78519741808209 |
| H | 6.82411572406807 | 1.69808868340382 | 3.93311777870356 |
| C | 6.49061390938192 | 1.79864373425082 | 6.13614604940379 |
| H | 7.38172723929829 | 2.29466773207203 | 6.49160383785352 |
| C | 4.38189915593049 | 0.89756217097568 | 6.06202870692568 |
| H | 3.38494936421509 | 0.59891524249212 | 6.35098102091890 |
| C | 5.36936591732636 | 1.43534121153580 | 6.92472211823156 |
| H | 5.25678399680147 | 1.60883220421714 | 7.98479931970004 |
| Be | 4.89823748332024 | 2.65405071919384 | 5.54123712579227 |
| C | 4.17868884475758 | 4.20534762552815 | 5.36331810560405 |
| H | 4.65686021600721 | 4.99565941000394 | 5.95262905822692 |
| H | 4.12405559038476 | 4.58194068264059 | 4.34201819209831 |
| H | 3.17000359758585 | 4.18371164409930 | 5.85669323750514 |
| C | 1.12250603665610 | 5.54227165319731 | 5.40690292665991 |
| O | 0.73499147415447 | 6.58825902161182 | 5.68562143049742 |

## T9B

| | | | |
|---|---|---|---|
| Mn | 1.74472482071086 | 3.94253438317690 | 5.08083721822143 |
| O | 1.86216186403877 | 3.33823750551973 | 7.94916117521235 |
| C | 0.95379102470314 | 3.82432240505414 | 3.09686153648736 |
| H | 1.10057589261425 | 4.55281634641667 | 2.31368978344572 |
| C | 1.21709012643001 | 1.97010656270658 | 4.44000901729643 |
| H | 1.60012422890276 | 1.04800182041260 | 4.84801014257956 |
| C | -0.10169129936054 | 3.82123506028828 | 4.06576217238551 |
| H | -0.88646514237165 | 4.55851430731565 | 4.15128540435923 |
| C | 0.05952887021867 | 2.68458999744687 | 4.89020142614937 |
| H | -0.58221785663341 | 2.40249682128069 | 5.71189081001971 |
| C | 1.75080440471061 | 2.67931105421692 | 3.33943990647707 |
| H | 2.66195565432418 | 2.41995985470532 | 2.81836427946479 |
| C | 1.87179837323726 | 3.59239055939118 | 6.82902110873764 |
| C | 4.73188484889204 | 4.13342431782255 | 4.96628100011798 |
| H | 4.87365386922743 | 4.81864332289470 | 5.80132446237169 |
| H | 5.43111038470380 | 4.39569747991716 | 4.16665228083493 |
| H | 3.74418310571389 | 4.35292136883591 | 4.50107794012537 |
| B | 4.90872895625260 | 2.62927601207429 | 5.37840832252161 |
| O | 5.29800882556668 | 2.23305376439162 | 6.63200370477671 |
| O | 4.75339437865448 | 1.57360825295448 | 4.51173001009250 |
| C | 5.21589274062024 | 0.78300162094267 | 6.67808028611912 |
| C | 5.30073969927101 | 0.39706667434181 | 5.16538435150947 |
| C | 6.35356397246961 | 0.25386542454116 | 7.53378881039068 |
| C | 3.87400582178428 | 0.43575364458675 | 7.31324829333309 |
| C | 6.73429631876094 | 0.24511402239165 | 4.66570285659908 |
| C | 4.47726806728128 | -0.81613715772471 | 4.77099540188536 |
| H | 6.20855604559445 | 0.57238960976263 | 8.56820115231782 |
| H | 6.36677509310884 | -0.83902866544587 | 7.51125595492794 |
| H | 7.31977057787152 | 0.62388956101870 | 7.19165165072939 |
| H | 3.76498288112433 | -0.64375392603462 | 7.43655569944382 |
| H | 3.81692645269833 | 0.90602162604304 | 8.29649695409028 |
| H | 3.04364936541183 | 0.80745197459142 | 6.71114766686035 |
| H | 7.20084756743636 | -0.65507777223052 | 5.07087719052570 |
| H | 6.72029540542110 | 0.16735959015568 | 3.57665125451910 |
| H | 7.34333151722115 | 1.10900912621294 | 4.94126132536202 |
| H | 4.59081596789669 | -1.00164665850286 | 3.70071363171795 |
| H | 4.82593310224640 | -1.70082878077642 | 5.31014052728438 |
| H | 3.41899337645799 | -0.67160515568296 | 4.98558162849693 |
| C | 1.51310673978242 | 5.69651596899074 | 5.33025405095554 |
| O | 1.27060397700385 | 6.81079807599617 | 5.46839961125397 |

## Methane

| | | | |
|---|---|---|---|
| C | 3.46911913886119 | 5.83586262044273 | 4.91238252724935 |
| H | 2.65233353006986 | 5.37046977429177 | 4.35873849565775 |
| H | 3.64689240240183 | 5.27988421831449 | 5.83407893539168 |
| H | 3.20378750490539 | 6.86603097202988 | 5.15449820412694 |
| H | 4.37346742376172 | 5.82709241492111 | 4.30219183757426 |

## Carbon Monoxide

| | | | |
|---|---|---|---|
| O | 1.51597801019372 | 2.01411037295095 | 7.38698737783116 |
| C | 1.77823198980627 | 2.71952962704904 | 6.55013262216884 |

## CpBeBeCp (3)

| | | | |
|---|---|---|---|
| C | 5.17148644647007 | 3.14819602616129 | 4.91979650357894 |
| H | 5.62507134735059 | 3.78058022127963 | 5.66858552434678 |
| C | 4.55611799432951 | 3.58785925654986 | 3.72299394094018 |
| H | 4.45881407315591 | 4.61387212869457 | 3.40024538086558 |
| C | 4.17479682191362 | 2.44384093908358 | 2.98124073351027 |
| H | 3.73599677691884 | 2.44558080939542 | 1.99443983034890 |
| Be | 6.07319877981942 | 2.44191196692607 | 3.35923020966968 |
| C | 5.17045195712305 | 1.73245969745056 | 4.91776120950214 |
| H | 5.62310931375901 | 1.09726381942691 | 5.66472962216658 |
| C | 4.55444368729473 | 1.29713861221742 | 3.71970152146592 |
| H | 4.45564390626445 | 0.27220087203944 | 3.39400533987736 |
| C | 8.70915620383589 | 2.43995494136496 | 0.65411594829923 |
| H | 8.16321492665469 | 2.43821705989645 | -0.27770923169934 |
| C | 9.08774964446993 | 3.58666275838691 | 1.39308189496165 |
| H | 8.88081252650748 | 4.61161147467271 | 1.12289073821682 |
| C | 9.70202344973392 | 3.15133607667569 | 2.59203545709225 |
| H | 10.04507599329025 | 3.78652391128202 | 3.39525745218784 |
| Be | 7.90940800337219 | 2.44192101241827 | 2.41690787184670 |
| C | 9.08940763160978 | 1.29593185338815 | 1.39638341836632 |
| H | 8.88394890925230 | 0.26991077277851 | 1.12915049836212 |
| C | 9.70304713990729 | 1.73560018301049 | 2.59407632117939 |
| H | 10.04701046696680 | 1.10322560690103 | 3.39912681518459 |

## $B_2Pin_2$

| | | | |
|---|---|---|---|
| C | -5.03128729364628 | 2.63316309274707 | 0.21922191826201 |
| C | -4.55471220300989 | 4.04663836641314 | -0.23811984347380 |
| O | -6.41800784070234 | 2.87644797600012 | 0.58013887073761 |
| O | -5.78917870364814 | 4.65037189973626 | -0.71184530465372 |
| C | -4.29898720856090 | 2.07219466257744 | 1.42559289562608 |
| H | -4.69870142817317 | 1.08504115900748 | 1.66732908531543 |
| H | -3.23336180701416 | 1.96571237132609 | 1.20548719525990 |
| H | -4.41513351863863 | 2.71378870396723 | 2.29873238378265 |
| C | -5.04611540700632 | 1.61032937707302 | -0.91312032989740 |
| H | -4.03194177529715 | 1.32687600626112 | -1.20234484151810 |
| H | -5.57176692016575 | 0.71637210812228 | -0.57151632029633 |
| H | -5.56541059038764 | 2.00097803802777 | -1.79095063982992 |
| C | -4.05677246567344 | 4.91335342979301 | 0.91469161093344 |
| H | -3.09413581496228 | 4.55817904449232 | 1.28859519512762 |
| H | -3.93275253111962 | 5.93709937494220 | 0.55585009575059 |
| H | -4.77209986143174 | 4.92189595222657 | 1.74000675242018 |
| C | -3.54179977195400 | 4.04289687687747 | -1.36958829440671 |
| H | -3.26914119064315 | 5.07086183991976 | -1.61794106695452 |
| H | -2.63576295882571 | 3.51245552816395 | -1.06442673023446 |
| H | -3.94271672364527 | 3.56746594365707 | -2.26439888331162 |
| B | -6.83010919520139 | 3.99779634625856 | -0.09759159508649 |
| B | -8.45362197611820 | 4.52054672908925 | -0.16870108994716 |
| O | -9.39879983798980 | 4.20945494644615 | 0.77813066569222 |
| O | -8.96244197293064 | 5.29738947436033 | -1.18075423123848 |
| C | -10.57897720084098 | 5.01002814246088 | 0.49734215723456 |
| C | -10.40631768503440 | 5.33560951520832 | -1.01875161238977 |

| | | | |
|---|---|---|---|
| C | -11.81702924464637 | 4.19931423817739 | 0.83849671909390 |
| C | -10.49376936865635 | 6.24517245957475 | 1.38936300074025 |
| C | -10.97892711181295 | 4.25642929027401 | -1.93308093688608 |
| C | -10.91318396367176 | 6.70398026115796 | -1.43942860539530 |
| H | -11.85172370512067 | 4.02323986908007 | 1.91576798013352 |
| H | -12.71877010598082 | 4.74688375312538 | 0.55159764040197 |
| H | -11.81586055399386 | 3.23441742456925 | 0.33207980065143 |
| H | -11.38106323932893 | 6.87236356987742 | 1.28055679284666 |
| H | -10.42315696227343 | 5.92216793642495 | 2.42997334833171 |
| H | -9.61043986427556 | 6.84278832606617 | 1.15379102417319 |
| H | -12.07078783604856 | 4.26221451704150 | -1.91455118930512 |
| H | -10.64869402496286 | 4.45159818450174 | -2.95537394841725 |
| H | -10.62805187767540 | 3.26409524186758 | -1.64141539087649 |
| H | -10.75125517140600 | 6.83845863077030 | -2.51105806471611 |
| H | -11.98524619716405 | 6.78755455143378 | -1.24191729385812 |
| H | -10.39500993036105 | 7.50325272090266 | -0.91018701982194 |

**HBeCp**

| | | | |
|---|---|---|---|
| C | 5.25902573866596 | 3.15107394819725 | 4.88401248258619 |
| H | 5.73177541851566 | 3.78498532141226 | 5.61941912981366 |
| C | 4.61495858585891 | 3.58871612744390 | 3.69926212464532 |
| H | 4.51241789888847 | 4.61378709827644 | 3.37541786796890 |
| C | 4.21685698486725 | 2.44144410226973 | 2.96769106172326 |
| H | 3.75866900946107 | 2.44105050514665 | 1.98981211371595 |
| Be | 6.08234043597246 | 2.44159203972259 | 3.32570666555839 |
| C | 5.25901783438078 | 1.73333426942612 | 4.88457907011868 |
| H | 5.73194623831036 | 1.10001768298212 | 5.62038502673929 |
| C | 4.61504995136760 | 1.29474404198008 | 3.70012725377610 |
| H | 4.51222179075292 | 0.26939569945089 | 3.37726342418735 |
| H | 7.26369002295850 | 2.44143772369195 | 2.68158532916690 |

**HBPin**

| | | | |
|---|---|---|---|
| C | -0.98409276263387 | 0.68702160336792 | 0.64144643577851 |
| C | -0.50065378080236 | 2.17109889673554 | 0.57007357813408 |
| O | -2.40370516502017 | 0.83479419248872 | 0.93078228097922 |
| O | -1.70835405806597 | 2.87308377318586 | 0.15812217056911 |
| C | -0.34232221243136 | -0.13952915115093 | 1.74159601022353 |
| H | -0.73863913664657 | -1.15663158049665 | 1.71083622191893 |
| H | 0.73973882433899 | -0.18918452511411 | 1.59426436822224 |
| H | -0.54392486978496 | 0.27927016753852 | 2.72714000276679 |
| C | -0.88422233128995 | -0.04129074039458 | -0.69505933603373 |
| H | 0.15641390777907 | -0.24609365752158 | -0.95481302166552 |
| H | -1.41550606291792 | -0.99194615473946 | -0.61784892680411 |
| H | -1.33767697605768 | 0.54252403565656 | -1.49896887009947 |
| C | -0.10729075337780 | 2.74047266222557 | 1.92936634308471 |
| H | 0.83016145346359 | 2.30582237743620 | 2.28240199218264 |
| H | 0.02615760037524 | 3.81972546779159 | 1.83264044146617 |
| H | -0.88265564529687 | 2.55488720044684 | 2.67580702704809 |
| C | 0.59306179849381 | 2.43584398054414 | -0.44923605838866 |
| H | 0.86503726438421 | 3.49318697322511 | -0.42497129894793 |
| H | 1.48299774559111 | 1.84784847990616 | -0.20980260005204 |
| H | 0.26868663715198 | 2.18677070887288 | -1.45922873291603 |
| B | -2.76727187993536 | 2.08415468077904 | 0.51161441878308 |
| H | -3.89994742731716 | 2.45061090921660 | 0.45899385375039 |

**MeBeCp**

| | | | |
|---|---|---|---|
| C | 5.26891502794241 | 3.15057529187649 | 4.87845316144045 |
| H | 5.74179396146092 | 3.78441506231580 | 5.61410030129846 |
| C | 4.62497751045721 | 3.58839749834857 | 3.69395811804703 |
| H | 4.52237462100741 | 4.61371387968331 | 3.37030080139173 |
| C | 4.22679788043438 | 2.44175898219697 | 2.96260769954627 |
| H | 3.76841940093429 | 2.44160108448818 | 1.98461356715778 |
| Be | 6.10741138215195 | 2.44151121650685 | 3.31183525064559 |
| C | 5.26840288567200 | 1.73343002679302 | 4.87887796845056 |
| H | 5.74156020897281 | 1.09983932995617 | 5.61457973095402 |
| C | 4.62502764036707 | 1.29508107680519 | 3.69448757909168 |
| H | 4.52173687586017 | 0.26960438563395 | 3.37159389745802 |
| C | 7.61814682856271 | 2.44197265896768 | 2.49174765360337 |
| H | 8.32629016285772 | 1.72618209064865 | 2.92622057662716 |
| H | 8.10654792210968 | 3.42332750310031 | 2.52309759764850 |
| H | 7.51587769120916 | 2.17707991267881 | 1.43253609663936 |

**MeBPin**

| | | | |
|---|---|---|---|
| C | -1.02633593762316 | 0.69753603081555 | 0.62655487681045 |
| C | -0.54752282105673 | 2.18251983770356 | 0.56772334597933 |
| O | -2.44179272613291 | 0.84097181077894 | 0.91207167573036 |
| O | -1.74774497696581 | 2.87623607326476 | 0.13905404235317 |
| C | -0.38271063356856 | -0.13635530895168 | 1.72064261655757 |
| H | -0.77427044015691 | -1.15512076943175 | 1.68050756983184 |
| H | 0.70008751242063 | -0.18021275370299 | 1.57628106988344 |
| H | -0.58888077143492 | 0.27287350688562 | 2.70937984472073 |
| C | -0.91704418412787 | -0.01869577637080 | -0.71635484310904 |
| H | 0.12500418206100 | -0.22036584767492 | -0.97331465134592 |
| H | -1.44791852565003 | -0.97061927673835 | -0.65106235093888 |
| H | -1.36703947344122 | 0.57255255749287 | -1.51693006829137 |
| C | -0.17778013219612 | 2.74560671786557 | 1.93695287695267 |
| H | 0.75651717320099 | 2.31510944207247 | 2.30349954892150 |
| H | -0.04982211563089 | 3.82638860970094 | 1.84897824610326 |
| H | -0.96408000415686 | 2.55034974679331 | 2.66959024666899 |
| C | 0.56457291966087 | 2.45425917854662 | -0.43021510032002 |
| H | 0.83424836413654 | 3.51207635340244 | -0.39658545742520 |
| H | 1.45168094094137 | 1.86697185076658 | -0.17846113985707 |
| H | 0.25836193123254 | 2.20947898449343 | -1.44702039510761 |
| B | -2.82367559018261 | 2.09401657167887 | 0.49150519256804 |
| C | -4.30919512766057 | 2.58728319938685 | 0.44609893293100 |
| H | -4.57301805203534 | 3.04023125985900 | 1.40842663678407 |
| H | -4.45466126645549 | 3.35210435323151 | -0.32038272090254 |
| H | -5.00780051517791 | 1.76650970813156 | 0.26980687450121 |

**References:**


(1) Hoffmann, F.; Wagler, J.; Roewer, G. Transition-Metal Compounds Containing Alkynylsilyl Groups – Cyclopentadienyl Complexes. *Eur. J. Inorg. Chem.* **2012**, *2012* (36), 6018–6026. https://doi.org/10.1002/ejic.201200707.

(2) Patton, A. T.; Patton, A. T.; Strouse, C. E.; Knobler, C. B.; Gladysz, J. A. Syntheses, Properties, and X-Ray Crystal Structures of Stable Methylidene Complexes of the Formula [(η-C5Me5)Re(NO)(L)(=CH2)]+PF6-. *J. Am. Chem. Soc.* **1983**, *105* (18), 5804–5811. https://doi.org/10.1021/ja00356a018.

(3) Boronski, J. T.; Crumpton, A. E.; Wales, L. L.; Aldridge, S. Diberyllocene, a Stable Compound of Be(I) with a Be–Be Bond. *Science (80-. ).* **2023**, *380* (6650), 1147–1149. https://doi.org/10.1126/science.adh4419.

(4) Buchner, M. R.; Müller, M. Handling Beryllium, the Safe Way. *ACS Chem. Heal. Saf.* **2023**, *30* (2), 36–43. https://doi.org/10.1021/acs.chas.3c00003.

(5) Schmidbaur, H. *Be Organoberyllium Compounds*; Kubny, A., Mirbach, M., Krüerke, U., Slawisch, A., Eds.; Springer Berlin Heidelberg: Berlin, Heidelberg, 1987; Vol. 21. https://doi.org/10.1007/978-3-662-06024-7.

(6) Bartke, T. C.; Bjorseth, A.; Haaland, A.; Marstokk, K.-M.; Møllendal, H. Microwave Spectrum, Structure and Dipole Moment of Cyclopentadienylberyllium Hydride. *J. Organomet. Chem.* **1975**, *85* (3), 271–277. https://doi.org/10.1016/S0022-328X(00)80300-9.

(7) Saulys, D. A.; Powell, D. R. Synthesis, Experimental/Theoretical Characterization, and Thermolysis Chemistry of CpBe(SiMe 3 ), a Molecule Containing an Unprecedented Beryllium−Silicon Bond. *Organometallics* **2003**, *22* (3), 407–413. https://doi.org/10.1021/om0201720.

(8) Drew, D. A.; Morgan, G. L. Synthesis and Spectroscopic Properties of Cyclopentadienyl(Methyl)Beryllium and Cyclopentadienylberyllium Halide Complexes. *Inorg. Chem.* **1977**, *16* (7), 1704–1708. https://doi.org/10.1021/ic50173a027.

(9) Cosier, J.; Glazer, A. M. A Nitrogen-Gas-Stream Cryostat for General X-Ray Diffraction Studies. *J. Appl. Crystallogr.* **1986**, *19* (2), 105–107. https://doi.org/10.1107/S0021889886089835.

(10) Agilent Technologies. CrysAlisPro.

(11) Sheldrick, G. M. SHELXT – Integrated Space-Group and Crystal-Structure Determination. *Acta Crystallogr. Sect. A Found. Adv.* **2015**, *71* (1), 3–8. https://doi.org/10.1107/S2053273314026370.

(12) Sheldrick, G. M. Crystal Structure Refinement with SHELXL. *Acta Crystallogr. Sect. C Struct. Chem.* **2015**, *71* (1), 3–8. https://doi.org/10.1107/S2053229614024218.

(13) Dolomanov, O. V.; Bourhis, L. J.; Gildea, R. J.; Howard, J. A. K.; Puschmann, H.

OLEX2 : A Complete Structure Solution, Refinement and Analysis Program. *J. Appl. Crystallogr.* **2009**, *42* (2), 339–341. https://doi.org/10.1107/S0021889808042726.

(14) Neese, F.; Wennmohs, F.; Becker, U.; Riplinger, C. The ORCA Quantum Chemistry Program Package. *J. Chem. Phys.* **2020**, *152* (22), 224108. https://doi.org/10.1063/5.0004608.

(15) Neese, F. The ORCA Program System. *WIREs Comput. Mol. Sci.* **2012**, *2* (1), 73–78. https://doi.org/10.1002/wcms.81.

(16) Lin, Y.-S.; Li, G.-D.; Mao, S.-P.; Chai, J.-D. Long-Range Corrected Hybrid Density Functionals with Improved Dispersion Corrections. *J. Chem. Theory Comput.* **2013**, *9* (1), 263–272. https://doi.org/10.1021/ct300715s.

(17) Caldeweyher, E.; Mewes, J.-M.; Ehlert, S.; Grimme, S. Extension and Evaluation of the D4 London-Dispersion Model for Periodic Systems. *Phys. Chem. Chem. Phys.* **2020**, *22* (16), 8499–8512. https://doi.org/10.1039/D0CP00502A.

(18) Canal Neto, A.; Ferreira, I. B.; Jorge, F. E.; de Oliveira, A. Z. All-Electron Triple Zeta Basis Sets for ZORA Calculations: Application in Studies of Atoms and Molecules. *Chem. Phys. Lett.* **2021**, *771* (March), 138548. https://doi.org/10.1016/j.cplett.2021.138548.

(19) Mardirossian, N.; Head-Gordon, M. ΩB97X-V: A 10-Parameter, Range-Separated Hybrid, Generalized Gradient Approximation Density Functional with Nonlocal Correlation, Designed by a Survival-of-the-Fittest Strategy. *Phys. Chem. Chem. Phys.* **2014**, *16* (21), 9904. https://doi.org/10.1039/c3cp54374a.

(20) Glendening, E. D.; Badenhoop, J. K.; Reed, A. E.; Carpenter, J. E.; A., B. J.; Morales, C. M.; Karafiloglou, P.; Landis, C. R.; Weinhold, F. NBO 7.0. Madison, WI 2018.

(21) Lu, T.; Chen, F. Multiwfn: A Multifunctional Wavefunction Analyzer. *J. Comput. Chem.* **2012**, *33* (5), 580–592. https://doi.org/10.1002/jcc.22885.

(22) Turner, J. J.; George, M. W.; Poliakoff, M.; Perutz, R. N. Photochemistry of Transition Metal Carbonyls. *Chem. Soc. Rev.* **2022**, *51* (13), 5300–5329. https://doi.org/10.1039/D1CS00826A.

(23) Wu, X.; Liu, Z.; Murphy, T. S.; Sun, X. Z.; Hanson-Heine, M. W. D.; Towrie, M.; Harvey, J. N.; George, M. W. The Effect of Coordination of Alkanes, Xe and CO 2 (η 1 -OCO) on Changes in Spin State and Reactivity in Organometallic Chemistry: A Combined Experimental and Theoretical Study of the Photochemistry of CpMn(CO) 3. *Faraday Discuss.* **2019**, *220*, 86–104. https://doi.org/10.1039/C9FD00067D.
